# Efficacy and effectiveness of hand hygiene-related practices used in community settings for removal of organisms from hands: a systematic review

**DOI:** 10.1136/bmjgh-2025-018925

**Published:** 2025-09-16

**Authors:** Stephen P Hilton, Nick H An, Lilly A O’Brien, Jedidiah S Snyder, Hannah K Rogers, Oliver Cumming, Joanna Esteves Mills, Bruce Gordon, Matthew C Freeman, Bethany A Caruso, Marlene K Wolfe

**Affiliations:** 1 Gangarosa Department of Environmental Health, Rollins School of Public Health, Emory University, Atlanta, Georgia, USA; 2 Woodruff Health Sciences Center Library, Emory University, Atlanta, Georgia, USA; 3 Department of Disease Control, London School of Hygiene and Tropical Medicine, London, UK; 4 Water, Sanitation, Hygiene and Health Unit, World Health Organization, Geneva, Switzerland; 5 Hubert Department of Global Health, Rollins School of Public Health, Emory University, Atlanta, Georgia, USA

**Keywords:** Global Health, Hygiene, Public Health, Systematic review

## Abstract

**Background:**

This systematic review collected and synthesised evidence on the efficacy and effectiveness of commonly used hand hygiene materials and methods for removing or inactivating pathogens on hands in community settings. The evidence was generated to support the development of the WHO Guidelines for Hand Hygiene in Community Settings.

**Methods:**

We searched PubMed, Web of Science, EMBASE, CINAHL, Global Health, Cochrane Library, Global Index Medicus, Scopus, PAIS Index, WHO IRIS, UN Digital Library and World Bank eLibrary, and consulted experts in March 2023 for studies published between 1 January 1980 and 29 March 2023. Eligible studies included laboratory and field studies measuring reduction in organisms on hands after washing as intended in community settings; healthcare settings were excluded. Two reviewers independently extracted data from each study; risk of bias was assessed using the Mixed Method Appraisal Tool and a laboratory-based quality assessment tool. Summary results in terms of log_10_ reduction in organisms on hands were calculated for categories with five or more data points from two or more studies.

**Results:**

Of 177 studies that met inclusion criteria, the majority focused on alcohol-based hand sanitiser (111, 63%) and handwashing with soap and water (110, 62%). Most evidence (119, 67%) assessed bacterial reductions and only 7 (4%) studies addressed enveloped viruses. Across studies, there was a >2 log_10_ reduction in bacteria after handwashing with soap and water (2.19 (95% CI 1.5 to 2.87)) or alcohol-based sanitisers (3.13 (95% CI 2.7 to 3.56)), and for viruses after handwashing with soap and water (2.03 (95% CI 1.45 to 2.62)). However, there was a <2 log_10_ reduction for viruses with alcohol-based sanitisers (1.86 (95% CI 1.37 to 2.35)).

**Conclusions:**

The ability to compare efficacy across materials and methods was limited due to the focus on bacteria and soap and water and alcohol-based sanitisers. Additional evidence quantifying the impact of handwashing on viruses (especially enveloped viruses), handwashing alternatives other than alcohol-based sanitisers, drying methods and microbial water quality, as well as the effectiveness of many of these products in the field would support more evidence-based recommendations.

**PROSPERO registration number:**

CRD42023429145.

**WHAT IS ALREADY KNOWN ON THIS TOPIC**

- Hand hygiene plays a critical role in preventing the spread of infectious disease, however there is a lack of data describing which methods are most efficacious for handwashing in community settings, particularly for handwashing alternatives and handwashing for viruses.
- Previous reviews have described the effectiveness of handwashing to prevent disease transmission; however, there has not been a thorough review describing the efficacy of handwashing for removing and inactivating organisms.

**WHAT THIS STUDY ADDS**

- This study provides an overview of the current knowledge on handwashing efficacy and demonstrates that there is substantial data to suggest that both handwashing with soap and water and use of alcohol-based hand sanitisers are efficacious for removing bacteria.
- However, there are gaps in knowledge around the efficacy of hand hygiene methods for removal and inactivation of viruses and for a wider range of handwashing methods that are commonly practiced around the world such as use of soap alternatives like ash and sand.

**HOW THIS STUDY MIGHT AFFECT RESEARCH PRACTICE OR POLICY**

- Handwashing guidelines across the globe demonstrate inconsistencies and contradictions and lack evidence to support some of the recommended practices.
- This study provides an overview of the current evidence to support handwashing guidelines and implementation of practices globally and indicates that handwashing with soap and water is typically an efficacious method.
- However, to formulate strong recommendations for handwashing methods across diverse contexts, more data are needed on a wider range of conditions.
- This review highlights these gaps, including a lack of data on viral pathogens and elements of handwashing methods such as the use of soap alternatives, drying methods, water quality and timing of washing.

## Introduction

 Hand hygiene is a critical and relatively inexpensive intervention to prevent many infectious diseases, and there are many options for materials and methods for hand hygiene including handwashing with soap or the use of other materials such as alcohol-based hand rubs (ABHRs).^1^ Handwashing interventions are particularly effective for preventing enteric and respiratory infections—caused by both bacterial and enveloped and non-enveloped viruses—which account for a large burden of disease^2^,^4^ and high healthcare costs.^5^ It is important to know about the efficacy and effectiveness of handwashing for the removal and inactivation of organisms that cause disease to establish global guidelines and recommendations for hand hygiene and protect public health.^6^

A recent scoping review identified 51 existing international hand hygiene guidelines and highlighted effective hand hygiene as one of four areas where clear recommendations are needed in community settings given a lack of consistent evidence-based recommendations.^7^ Effectiveness of hand hygiene depends on many factors, including the efficacy of hand hygiene materials and methods and how these methods are put into practice. Efficacy, or the ability of a material and method to remove organisms from hands, is a foundational aspect of effectiveness. Any method that is not efficacious in a laboratory setting will have limited effectiveness at removal of organisms and prevention of disease transmission in a real-world environment. The recent scoping review^7^ also found that guidelines for hand hygiene generally agree on what methods are presumed to be effective for hand hygiene; however, it is unclear how evidence-based these guidelines are because there are many inconsistencies and gaps in the evidence about how to practice hand hygiene to most efficaciously remove and inactivate organisms from hands, and their effectiveness in practice.

While the guidelines often recommended handwashing with soap and water, many hand hygiene options are available, including the use of materials that range from soap to hand sanitisers (both ABHRs and non-alcoholic antiseptics), antimicrobial wipes and soap substitutes such as ash and sand. Hand hygiene materials and methods are designed to act in different ways to remove and inactivate organisms. Handwashing with soap and water (or substitutes) is intended to remove organisms from hands, while handwashing with soap with added antibacterial properties is intended to both remove and inactivate organisms. Use of an antiseptic rub is intended to inactivate organisms but does not wash away organisms or other materials soiling hands. Because the mechanisms of hand hygiene are different among these approaches and may impact different pathogens differently, it is important to establish any differences in efficacy of these hand hygiene options. Among the range of hand hygiene options, the comparative efficacy of materials and methods that are commonly used in community settings for removing or inactivating pathogens is not well-established.

The Ottawa Charter defines community settings as places where ‘health is created and lived by people within the setting of their everyday life; where they learn, work, play and love’,^8^ and include domestic, public and institutional spaces.^7^ Guidelines for hand hygiene in healthcare settings are well-established,^9^,^12^ and there is an abundance of data from both laboratory and real-world settings on the efficacy and effectiveness of hand hygiene. Many guidelines emphasise investing in hand hygiene as a core public health measure.^13^,^16^ However, studies on hand hygiene materials and methods that are specifically for use in community settings are critical, as aspects of handwashing materials and methods such as soap preparations, active ingredients and washing and scrubbing practices differ greatly from healthcare settings to community settings. To help establish evidence-based recommendations for hand hygiene in community settings, it is important to draw on the literature both for efficacy of handwashing materials and methods that are used in these settings from laboratory evidence, and from studies of effectiveness that have been conducted in field settings. The hand hygiene materials and methods that are intended for use in community settings are often investigated using standardised in vivo testing methods for evaluating handwashing efficacy in a laboratory, such as methods ASTM E2755-15 in the USA and EN1500 in Europe.^17 18^ These methods typically involve inoculating a volunteer’s hands with a test organism at higher levels than found in natural settings. It is possible to make quantitative comparisons between handwashing materials and methods by enumerating the organisms on hands before and after handwashing. The results from this testing are usually expressed in terms of log_10_ reduction in organisms on hands. Similar testing may also be done in field studies and on naturally contaminated hands, but it is more difficult to enumerate change in such settings. Previous reviews have primarily focused on soap products used as recommended by the WHO,^19^,^21^ as well as ABHR.^22^ There has been relatively less work done to review efficacious alternatives for hand hygiene when soap or ABHR are not available or are used ‘imperfectly’,^22^,^25^ or for understanding which drying methods prevent recontamination.^26^

The aim of this systematic review was to assess the effectiveness and/or efficacy of hand hygiene materials and methods that are intended for use in community settings for removing or inactivating pathogens from hands. It does not aim to assess the effectiveness of handwashing for disease prevention. The priority question and subquestions for this review were generated through an extensive consultation process by the WHO with external experts^27 28^ following a scoping review of current international guidelines.^7^

## Methods

### Research questions

This systematic review addressed four questions related to effective/efficacious hand hygiene: (1) How efficacious/effective are soap products at removing or inactivating key pathogens (or organisms intended as their surrogates) and how does duration impact efficacy and effectiveness?, (2) Where soap and/or water are not available, what are appropriate alternatives for hand hygiene?, (3) Which hand-drying methods are effective at reducing risk of recontamination of washed hands? and (4) What microbial water quality is required for effective handwashing with soap? In the handwashing literature, laboratory experiments typically provide data on the efficacy of methods and field studies demonstrate their effectiveness in practice. We focused our review on the materials and methods that are intended for use in community settings to make sure that all the evidence, laboratory or field, was directly related to these settings. Thus, both types of studies were sought in this review as described further in "Selection Criteria" below.

### Search strategy

This review was preregistered with PROSPERO (registration number: CRD42023429145) and is reported in accordance with the Preferred Reporting Items for Systematic Reviews and Meta-Analyses (PRISMA) criteria^29^ (online supplemental S1 – PRISMA Checklist). This review was part of an integrated protocol for multiple related reviews to synthesise the evidence for effective hand hygiene in community settings.^28^ We adopted a two-phased approach for identifying relevant studies. Phase 1 involved a broad search to capture all studies on hand hygiene in community settings that were relevant across multiple related systematic reviews. The outcome of phase 1 was a reduced sample from which further screening, specific to this review, was performed. A full description of the procedures followed for searches (including search terms), study inclusion, outcomes data collection, analysis and reporting of the multiple related reviews is presented in the published protocol.^28^

The phase 1 database search included studies published through 29 March 2023 that were published in English or had titles and abstracts published in English. We searched 12 peer-reviewed and grey literature databases. PubMed, Web of Science, EMBASE (Elsevier), CINAHL (EBSCOhost), Global Health (CAB), Cochrane Library, Global Index Medicus, Scopus (Elsevier), Public Affairs Information Service (PAIS) Index (ProQuest) were searched on 23 March 2023 and WHO Institutional Repository for Information Sharing (IRIS), UN Digital Library and World Bank eLibrary were searched on 28 March 2023 using search terms related to hand hygiene broadly and restrictions on terms related to healthcare settings in the titles.^28^ We searched trial registries (International Clinical Trials Registry Platform and ClinicalTrials.gov) for trials related to hand hygiene in community settings on 29 March 2023.

To ensure an exhaustive search, we conducted manual searches of reference lists of eight relevant systematic reviews.^19^,^26^ For reviews that provided a list of the reviewed articles, we searched only those references. If a list was not available, we searched all references and screened for potentially relevant titles. These reviews had 221 total references, of which 120 were duplicates, 33 were already identified in our database search and 68 were added to phase 2 title and abstract screening. Lastly, we contacted 35 content experts and organisations, using snowballing methods, from April to May 2023 for information on relevant unpublished literature.

### Selection criteria

For this review, hand hygiene refers to any hand cleansing undertaken for the purpose of removing or inactivating pathogens from hands. Efficacious hand hygiene is defined as hand cleansing which effectively removes or inactivates pathogens from hands.^10^ While hand hygiene does have the potential to limit disease transmission, quantifying reductions in risk and health outcomes is beyond the scope of this review. The term community settings included domestic (eg, households), public (eg, markets, public transportation hubs, vulnerable populations (eg, people experiencing homelessness), parks, squares or other public outdoor spaces, shops, restaurants and cafes) and institutional (eg, workplace, schools and universities, places of worship, prisons and places of detention, nursing homes and long-term care facilities) settings.^7^ Studies were excluded if they were in healthcare settings or were animal research. Studies focusing on care providers’ hand hygiene in nursing homes, home-based care settings and long-term care facilities were excluded during phase 2 screening as evidence they generated was determined to be similar to that from healthcare settings. Studies published in a language other than English were excluded during phase 2 screening due to the research team’s lack of proficiency in other languages, limiting the ability to extract and interpret data from studies in languages other than English. There were no geographical restrictions.

Eligible studies were laboratory efficacy and field effectiveness studies in which hands were either experimentally inoculated or naturally contaminated. Publications not based on empirical research were excluded. Eligible interventions were handwashing with soap and water at varying durations; other handwashing materials including antiseptics, friction-generating materials and water alone for varying durations; any hand drying method after handwashing with water or soap and water; and hand washing with soap and water contaminated with micro-organisms (naturally or experimentally). To meet the eligibility criteria, interventions needed to be in the general population in community settings or laboratory-based studies on interventions used in community settings (ie, surgical scrubs and similar were excluded). Data were also excluded if it represented results after multiple rounds of handwashing, including intermediary steps from handwashing to sample recovery that might affect microbial outcome, or used gloved hands or focused on fingernails only. Studies included reported results of handwashing and drying using the measurement of an appropriate organism before and after handwashing and expressed in terms of log_10_ or per cent reduction/increase in organisms on hands. Results were standardised so that all were expressed in log_10_ reduction in concentration of organisms on hands. This is a standard unit of reduction used in the field; a 1 log_10_ reduction corresponds to a 90% reduction in organisms, 2 log_10_ reduction to 99% reduction, and so on.

For hand hygiene materials and methods, handwashing with soap and water included any soap-based product (with or without antimicrobial additives) that was used in combination with water to wash hands, alcohol-based rubs included any alcohol-based product that did not require water, non-alcoholic antiseptic included any antiseptic product with a non-alcohol-based primary ingredient that did not involve water, and soap alternatives included any non-antiseptic and non-alcoholic product or material that was used for washing with water. Results were also considered based on the type of organism used for testing: bacteria (gram-positive or gram-negative), virus (enveloped or non-enveloped) and fungi.

We used Covidence software for systematic reviews.^30^ In both phases, screening of each article (phase 1: title and abstract only and phase 2: title and abstract, then full text review) was performed independently by two reviewers (KF, NHA), with discordance between reviewers reconciled by a third reviewer (MKW). The stages of the search and screening process are described in the PRISMA flow diagram (online supplemental S2 – PRISMA Flow Diagram).

### Data analysis

Two reviewers (KF, NHA) independently extracted data using customised data extraction tools (onlinesupplemental S3  Covidence Extraction Template and S4 ) and assessed risk of bias for each article using the Mixed Method Appraisal Tool (MMAT).^31 32^ Relevant studies were also assessed using a tool to evaluate the quality of laboratory-based microbiological studies.^33^ Any conflicts between reviewers over data extraction and bias assessment were resolved by discussion and agreement with another individual (MKW). Funnel plots were created to screen for the risk of publication bias.

We extracted summary-level results (ie, log_10_ or per cent reduction/increase) of handwashing and drying using the measurement of an appropriate organism before and after handwashing and/or drying. Results were stratified by the type of micro-organism studied (eg, bacteria, enveloped viruses, non-enveloped viruses), as well as by handwashing and/or drying materials and methods, which include hand hygiene materials (eg, soap, antiseptics, material for drying), the duration of handwashing and whether rubbing of hands was part of the handwashing method. Results were synthesised for categories with five or more data points from two or more studies by performing a meta-analysis of studies that reported a log_10_ or per cent reduction within categories of handwashing material/method along with a measure of spread. Studies were also required to specify the type of organism tested and handwashing materials used. Studies that reported concentrations before and after handwashing and did not compute a reduction value, those with results shown just in a figure, and studies that did not provide useable estimates of uncertainty were unable to be included in meta-analysis. For studies with over 10 measures that were missing log_10_ reduction or spread data, we contacted the corresponding author to request that the missing items be shared for inclusion, but we did not receive any additional data from these authors.

Analysis was conducted in R (V.4.3.1), using the ‘metafor’ package. Prior to meta-analysis, results expressed in terms of per cent reduction were transformed to log_10_ reduction values using a Wilson CI approach to estimate the CI. In cases where the upper limit of the CI was 100% pathogen removal, a value of 5 log_10_ reduction was imputed. Summary log_10_ reduction values were estimated using a multilevel random-effects meta-analysis using the inverse-variance method. While there is no clear threshold for efficacy in the literature, we used a 2-log_10_ reduction in organisms on hands as a comparison standard for efficacious hand hygiene,^34^ as has been applied in clinical^35^ and food safety settings^36^ by the US Food and Drug Administration. The restricted maximum-likelihood method was used to estimate between-study variance (tau^2^), and Hartung-Knapp adjustments were used to adjust test statistics and CIs for random effects. Because studies often reported multiple effect sizes per meta-analysis (eg, results from two different antibacterial soaps), a two-level random effects structure was used to model heterogeneity between and within studies.

The ‘dmetar’ package was used to calculate the amount of heterogeneity that can be explained by differences between studies using I^2^ at the study level (heretofore listed as I^2^_study_). The higher the value, the more of the variation is described by heterogeneity between studies, with the rest being due to variation within studies and random error. Our interpretation of heterogeneity was inspired by a previous review.^37^ We considered I^2^_study_ values less than 33% to indicate low heterogeneity between studies, between 33% and 66% to indicate moderate heterogeneity, and more than 66% heterogeneity to indicate high heterogeneity.^37^ The quality of evidence for an association between hand hygiene methods and their efficacy or effectiveness at removing or inactivating pathogens was evaluated in accordance with the Grading of Recommendations Assessment, Development and Evaluation system.^38^

### Ethics and patient/public involvement statement

Because we reviewed published documents, no ethical approval was required. Patients or the public were not involved directly in the design, conduct, reporting or dissemination plans of our research. This evidence synthesis supports the forthcoming WHO Guidelines for Hand Hygiene in Community Settings; the study questions were developed in broad consultation with a network of key partners. Findings from this review will be disseminated alongside the Guidelines.

### Data availability

All data and extraction templates will be made publicly available on publication at Figshare (https://doi.org/10.6084/m9.figshare.28370069).^39^

## Results

### Characteristics of the studies included in this review

We identified 177 manuscripts that met inclusion criteria (online supplemental S5 – Bibliography of Included Studies). Of these, 160 (90%) reported on laboratory-based studies and 18 (10%) reported on field-based studies, including one manuscript that reported both study types (table 1). A large percentage of studies (62%, n=110) included data on handwashing with soap and water. A similar number of studies included data on ABHRs (63%, n=111). Fewer manuscripts reported studies on handwashing with water only (18%, n=32), non-alcoholic antiseptics (14%, n=25), handwashing with soap alternatives (8%, n=15), hand drying (7%, n=12) and handwashing with antimicrobial wipes (6%, n=10). About half of the studies (53%, n=94) studied multiple of these hand hygiene materials. Most studies occurred in the Region of Americas and European Region WHO region (table 1), predominantly in the USA, the UK and Germany (online supplemental S6 – Summary of Included Studies). Some studies occurred in multiple countries. An overview of all included manuscripts and their characteristics, including MMAT assessment results and laboratory quality assessment results, is listed in online supplemental S6 – Summary of Included Studies.

**Table 1:** Characteristics of the included studies

Descriptive characteristics of studies	Total n (%)
Total number of studies	177 (100)
Study type*	
General population in community studies	18 (10)
Laboratory-based studies on interventions used in community setting	160 (90)
Hand hygiene materials*	
Handwashing with soap and water	110 (62)
Handwashing with water only	32 (18)
Handwashing with duration	100 (59)
Handwashing with microbial water quality	2 (1)
Hand rubbing with alcohol-based hand rub	111 (63)
Hand rubbing with non-alcoholic antiseptics	25 (14)
Handwashing with soap alternatives	15 (8)
Hand rubbing with antimicrobial wipes	10 (6)
Hand drying	12 (7)
WHO region of study	
Region of Americas	87 (49)
European Region	60 (34)
Western Pacific Region	19 (11)
South-East Asian Region	11 (6)
African Region	6 (3)
Eastern Mediterranean Region	3 (2)
Study quality rating (mean)	
Mixed Method Appraisal Tool	4.2
Laboratory Quality Assessment	4.81

* Descriptive statistics for study setting and hand hygiene materials represent variables where multiple options could be applicable, so the sum of sections may be over the study total.

### Quality of the studies included in this review

Overall, the mean study quality was 4.2 by the MMAT Assessment, and 4.81 by the laboratory assessment (n=177). Full MMAT quality appraisal scores and laboratory quality scores for each included study are available in online supplemental S7 – Bias Assessments. Funnel plots of the effect size and SE of estimates across studies show asymmetric patterns (online supplemental Figure S8 – Funnel Plots).

### Handwashing with soap and water

A total of 110 (62%) studies reported results related to the efficacy or effectiveness of soap products for removing and inactivating key pathogens (table 1). From these, 32 studies yielded 119 data points on bacteria, 4 studies yielded 25 data points on viruses in the laboratory and 2 studies yielded 25 data points on bacteria from the field (table 2, onlinesupplemental S9  Expanded Tables and S10 ). From laboratory experiments with bacteria, there were 43 data points on plain soap, 41 data points on soap with antimicrobial ingredients and 10 data points on water alone. Data were insufficient to summarise by pathogen subcategory (eg, enveloped, non-enveloped) or soap type for viruses or for field studies.

**Table 2:** Meta-analysis of studies reporting results on handwashing with soap and water

Study type	Hand rubbing	Handwashing materials	Type of organism	Summary log_10_ reduction (CI)	I^2^_Study_ (%)	Total studies	Total estimates	Total subjects
Laboratory	Any response	Any soap	Bacteria	2.48 (2.12 to 2.84)	74	32	119	1388
			Virus	2.03 (1.45 to 2.62)	86	4	25	126
		Water only	Bacteria	1.54 (0.83 to 2.24)	86	8	14	150
			Virus	1.55 (0.74 to 2.35)	94	5	9	58
	Yes	Any soap	Bacteria	2.36 (1.98 to 2.74)	65	25	90	894
			Gram-positive	1.36 (0.43 to 2.29)	98	7	27	214
			Gram-negative	2.6 (2.33 to 2.86)	25	21	63	680
		Plain soap	Bacteria	2.34 (1.9 to 2.79)	90	20	43	530
		Antimicrobial soap	Bacteria	2.15 (1.4 to 2.89)	95	13	41	340
		Water only	Bacteria	1.4 (0.52 to 2.28)	82	6	10	114
		Any soap	Virus	2.3 (1.48 to 3.12)	92	3	13	90
			Non-enveloped	1.71 (1.19 to 2.23)	80	2	12	78
		Plain soap	Virus	2.42 (0.78 to 4.05)	96	3	5	66
Field	Any response	Any soap	Bacteria	0.44 (−0.08 to 0.96)	83	2	25	1412
	Yes	Any soap	Bacteria	0.48 (−0.02 to 0.97)	85	2	24	1408
			Gram-positive	0.5 (−0.08 to 1.08)	93	2	8	688

The summary log_10_ reduction includes a 95% CI. This table only shows comparisons where enough data were available to complete a meta-analysis. S9 also shows comparisons that do not have enough data to complete a meta-analysis, as well as mean MMAT and laboratory scores.

MMAT, Mixed Method Appraisal Tool.

Washing with soap and water resulted in a summary log_10_ reduction value (LRV) for bacteria of 2.48 (95% CI 2.12 to 2.84); summary LRVs for bacteria ranged from a low of 1.36 (95% CI 0.43 to 2.29) for gram-positive bacteria to a high of 2.6 (95% CI 2.33 to 2.86) for gram-negative bacteria (table 2, online supplemental S11 – Forest Plots). For bacteria, the estimated log LRV range was similar whether the soap used was antibacterial (2.15, 95% CI 1.4 to 2.89) or not (2.34, 95% CI 1.9 to 2.79) (table 2). For viruses, data revealed a 2.03 (95% CI 1.45 to 2.62) summary LRV for handwashing with soap and water; when analysis was restricted to studies using plain soap and water for viruses, we found a summary LRV of 2.42 (95% CI 0.78 to 4.05) (table 2, online supplemental S11 – Forest Plots). For handwashing with water only, there were eight studies with data that could be summarised for bacteria, revealing a summary LRV of 1.54 (95% CI 0.83 to 2.24), and five studies with data that could be summarised for viruses, resulting in a summary LRV of 1.55 (0.74 to 2.35) (table 2, online supplemental S11 – Forest Plots). There was insufficient data for meta-analysis on handwashing experiments that did not involve rubbing hands. For field studies, LRVs ranged from 0.4 (95% CI –0.08 to 0.96) for any soap and water methods for bacteria to 0.5 (95% CI –0.08 to 1.08) for soap and water with rubbing for gram-positive bacteria (table 2). Results from the field were insufficient to summarise for viruses. Heterogeneity was high among all groups, with I^2^_Study_ ranging from 0.65 to 0.98, with a median of 0.85, with the exception of any soap and water for gram-negative bacteria, which had an I^2^_Study_ of 0.25. The I^2^_Study_ values for field studies were typically lower than laboratory studies, though there was considerably less data for field studies.

#### Duration of handwashing with soap and water

A total of 100 studies reported information on the duration of handwashing with soap and water (table 1). From these, there was enough data for meta-analysis related to bacteria over time periods of 10–20 s (21 data points from 6 studies) and 30+ s of washing (60 data points from 20 studies) (table 3, onlinesupplemental S9  Expanded Tables and S10 ). The largest number of studies reported on handwashing for 30+ s. Summary LRVs ranged from a minimum of 1.89 (95% CI 0.97 to 2.81) for handwashing with antibacterial soap for 10–20 s to 2.49 (95% CI 2.01 to 2.96) for handwashing for 30+ s with any soap. However, there was very little difference among log_10_ reduction estimates of bacteria across the time categories. Heterogeneity was also high, with I^2^_Study_ typically ranging from 0.85 to 0.99, though two comparisons with few studies had I^2^_Study_ values of 0 and 0.46.

**Table 3:** Meta-analysis of studies reporting results on the duration of handwashing for removal of bacteria with soap and water

Study type	Handwashing duration	Handwashing materials	Type of organism	Summary log_10_ reduction (CI)	I^2^_Study_ (%)	Total number of studies	Total number of estimates	Total number of subjects
Laboratory	10–20 s	Any soap	Bacteria	2.19 (1.5 to 2.87)	46	6	21	184
		Plain soap		2.45 (1.75 to 3.15)	96	5	7	118
		Antibacterial soap		1.89 (0.97 to 2.81)	0	3	8	42
		Water only		0.93 (−0.9 to 2.75)	85	3	6	63
	30+ s	Any soap	Bacteria	2.49 (2.01 to 2.96)	90	20	60	637
		Plain soap		2.36 (1.78 to 2.93)	99	16	32	376
		Antibacterial soap		2.34 (1.47 to 3.22)	98	10	28	261

The summary log_10_ reduction includes a 95% CI. This table only shows comparisons where enough data were available to complete a meta-analysis. S9 also shows comparisons that do not have enough data to complete a meta-analysis, as well as mean MMAT and Laboratory scores.

MMAT, Mixed Method Appraisal Tool.

### Handwashing with antiseptics and soap alternatives

Studies reporting the efficacy and effectiveness of alternatives to soap and water assessed alcohol-based antiseptics (111 studies), non-alcoholic antiseptics such as chlorhexidine, chlorine and iodine (25 studies), soap alternatives (15 studies) and antimicrobial wipes (10 studies) for removing and inactivating key pathogens (table 1). Studies eligible for meta-analysis yielded 117 data points on bacteria and 93 for viruses (33 with rubbing and 51 without) using alcohol-based antiseptics, 52 data points on bacteria and 12 on viruses using non-alcoholic antiseptics, 11 on bacteria using soap alternatives and 12 data points on bacteria and 1 on viruses using antimicrobial towels (online supplemental S10 – Summary of Estimates). The most data were available for alcohol-based antiseptics. Much of the data on viruses was focused on non-enveloped viruses; we were not able to generate any summary LRVs for enveloped viruses only. Results of meta-analysis ranged from 1.1 (95% CI 0.3 to 1.89) summary LRV for non-enveloped viruses with alcohol-based antiseptics to 3.24 (95% CI 2.71 to 3.76) summary LRV for gram-negative bacteria with alcohol-based antiseptics (table 4, onlinesupplemental S9  Expanded Tables and S11 ). Overall, estimates of log_10_ reduction were imprecise for alcohol-based antiseptics (table 4).

**Table 4:** Meta-analysis of studies reporting results on handwashing with soap alternatives

Study type	Handwashing materials	Hand rubbing	Type of organism	Summary log_10_ reduction (CI)	I^2^_Study_ (%)	Total studies	Total estimates	Total subjects
Laboratory	Alcohol-based hand rub	All responses	Bacteria	3.13 (2.7 to 3.56)	79	31	117	1566
		Yes	Bacteria	3.12 (2.59 to 3.65)	82	24	99	1300
			Gram-positive	2.24 (−0.36 to 4.84)	99	3	7	58
			Gram-negative	3.24 (2.71 to 3.76)	78	21	92	1242
	Non-alcohol-based antiseptics	All responses	Bacteria	2.54 (2.13 to 2.96)	18	7	40	388
		Yes	Bacteria	2.49 (2.02 to 2.97)	21	5	37	367
			Gram-positive	2.37 (0.97 to 3.77)	–	2	8	52
			Gram-negative	2.50 (2.11 to 2.89)	77	5	29	315
	Soap alternatives	All responses	Bacteria	2.56 (1.31 to 3.81)	96	4	11	160
		Yes	Bacteria	2.55 (1.21 to 3.89)	91	4	7	100
			Gram-negative	2.96 (1.78 to 4.15)	84	3	6	88
	Antiseptic/antimicrobial towels	All responses	Bacteria	2.13 (0.72 to 3.55)	82	5	12	175
		Yes	Bacteria	2.20 (0.67 to 3.73)	76	5	9	127
			Gram-negative	2.47 (0.66 to 4.27)	77	4	8	121
	Alcohol-based hand rub	All responses	Virus	1.86 (1.37 to 2.35)	56	13	93	792
		Yes	Virus	1.59 (0.72 to 2.45)	78	7	33	315
			Non-enveloped	1.10 (0.30 to 1.89)	66	5	29	273
		No	Virus	2.05 (1.44 to 2.66)	45	6	51	399
			Non-enveloped	2.05 (1.44 to 2.66)	45	6	51	399
	Non-alcohol-based antiseptics	All responses	Virus	1.91 (1.05 to 2.76)	87	4	12	78
		Yes	Virus	2.36 (0.23 to 4.49)	81	2	6	54

The summary log_10_ reduction includes a 95% CI. This table only shows comparisons where enough data were available to complete a meta-analysis. S9 also shows comparisons that do not have enough data to complete a meta-analysis, as well as mean MMAT and Laboratory scores.

MMAT, Mixed Method Appraisal Tool.

While soap alternatives as a whole resulted in a 2.56 summary LRV in bacteria (95% CI 1.31 to 3.81), data eligible for meta-analysis was limited to four studies and the hygiene materials and methods studied differed widely (table 4, online supplemental S11 – Forest Plots). Laboratory studies on soap alternatives reported on the use of ozonised/ozonated water (four studies), sand (three studies), ash (three studies) and powdered *Moringa oleifera* leaves (two studies) and similar products (online supplemental S6 – Summary of Included Studies). Studies on ozonised/ozonated water found substantial reduction in bacteria on hands; however, some found a higher reduction from traditional soap or antiseptics,^40 41^ while others did not find a significant difference.^41 42^ For sand, one study^43^ found that use of sand resulted in a higher reduction of enteroviruses compared with soap and water (but no statistical comparison was applied), and another study^44^ found that both sand and ash resulted in an LRV similar to soap and water and water only when all were used for a full 20 s. Although believed to have antimicrobial properties, both studies on *M. oleifera* powder use found it ineffective for handwashing in multiple preparations, though one^45^ found that a larger volume of powder did result in reductions similar to soap and water.^46 47^

A total of 10 studies on antimicrobial towels included both single use and reusable towels with results primarily on bacteria (9 studies) with 2 studies including viruses and 1 with fungi (online supplemental S6 – Summary of Included Studies). For bacteria, data summarised from five studies demonstrated a summary LRV of 2.13 (0.72 to 3.55). Heterogeneity was high (I^2^=0.82) (table 4).

### Hand drying methods

Overall, 12 studies reported on the impact of drying methods on hand contamination; however, most lacked the data necessary for inclusion in a meta-analysis (onlinesupplemental S6  Summary of Included Studies and S10 ). Included studies investigated hand drying using paper towels, cloth towels, evaporation, hot air dryers and jet air dryers. These studies vary in which drying materials are identified as resulting in the greatest decrease or lowest rate of recontamination of hands. While studies that tested paper towels generally reported reductions in contamination (online supplemental S9 – Expanded tables), no drying material was consistently identified as showing the greatest reduction across studies. Only one study^48^ included all the information required for inclusion in meta-analysis; however, this was not carried out because they are from a single study. They performed a laboratory experiment on the effects of six hand drying materials and found that jet air dryers were the most effective in reducing microbial loads on washed hands.

### Microbial water quality

Two studies assessed microbial quality of water for handwashing, and neither investigated the use of soap (online supplemental S9 – Expanded tables).^49^ investigated the impact of water of poor microbial quality on handwashing in a field study in Nepal; they found that handwashing was performed without soap while hands were highly contaminated and concluded that handwashing with poor quality water did not impact bacterial removal.^47^ Conducted a laboratory experiment to determine the efficacy of the Supertowel (a reusable microfibre towel with antimicrobial properties) and found that there was no difference in efficacy when it was used with water contaminated with *Escherichia coli*.

## Discussion

This systematic review aimed to assess the efficacy and effectiveness of a wide range of handwashing methods used in community settings globally. We considered any hygiene material used for handwashing (eg, soap and water, alcohol-based sanitiser or non-traditional materials), and assessed differences in the removal of organisms from hands. We found that common methods used and recommended for handwashing typically meet standards for efficacious hand hygiene. However, despite the substantial body of evidence on the efficacy of handwashing with soap and water for removal of bacteria, there is a lack of data on other organisms, handwashing methods that do not meet WHO recommendations for handwashing (ie, reduced duration of handwashing, use of alternatives such as ash and sand or water only), and how much less efficacious these methods might be. The majority of studies focused on bacteria, followed by a number of studies on viruses—but there is little to no data on other pathogens such as fungi and protozoa. The majority of studies included in the review were studies of laboratory efficacy; data from field studies describing effectiveness for removal and inactivation of organisms was limited. Laboratory studies investigating methods less commonly used or practiced imperfectly were also rare.

We found that the most commonly recommended hand hygiene materials—handwashing with soap and rubbing alcohol-based antiseptics—resulted in a >2 log_10_ reduction in organisms on hands after handwashing in laboratory-based experiments for bacteria, but that handwashing with soap resulted in a >2 log_10_ reduction in viruses while alcohol-based antiseptics did not. Although there are no standard criteria for handwashing in community settings,^34^ we adopted for comparison a threshold of 2 log describing efficacious handwashing as has been applied in both clinical settings^35^ and food safety settings^36^ by the US Food and Drug Administration, since these are both settings with greater regulations. Standard test methods for evaluating antiseptics often look at a comparison of the performance of a reference antiseptic wash to establish efficacy; however, these are challenging to compare across studies and do not apply to studies on soap or soap alternatives. Thus, using a 2-LRV threshold, we found that the majority of materials commonly used for handwashing meet standards for efficacious hand hygiene. However, study results were highly heterogeneous and the data on handwashing with soap and water for viruses was especially limited. There was generally more data available for bacteria than for viruses, and there is a particular lack of data for enveloped viruses. This is of concern as enveloped viruses, such as SARS-CoV-2, have caused significant outbreaks in recent years, and there are potential differences in the efficacy of hand hygiene for different classes of organisms.

### Handwashing with soap

Handwashing with soap and water resulted in a >2 summary LRV for experiments focused on bacteria, and in some cases for viruses. This data for viruses, however, was limited to only four studies and was highly heterogenous. When only non-enveloped viruses were included, the summary LRV was <2. This limited data suggests viruses, especially non-enveloped viruses, may be removed and inactivated by handwashing less efficaciously than bacteria but is not robust enough to be conclusive. This means it is especially important that further research is done on viruses to determine efficacy across methods more robustly. Few studies investigated the efficacy of handwashing with water alone, but for both bacteria and viruses summary LRVs were <2. It is critical to generate more data on viruses and handwashing with and without soap to provide better information on what aspects of these methods most impact the efficacy of handwashing.

Meta-analyses found plain soap to be similarly effective to antimicrobial soap for removal and inactivation of bacteria. Summary LRVs were similar across both plain (2.34, 95% CI 1.9 to 2.79) and antimicrobial (2.15, 95% CI 1.4 to 2.89) soap, and the lack of apparent advantage from antimicrobial soap is not surprising. Previous studies have failed to show any advantage conferred by antibacterial soap compared with plain soap; as such, many antibacterial additives have subsequently been banned in the USA.^50 51^ The summary LRV was below 2; however, for gram-positive bacteria with the use of any soap (1.36, 95% CI 0.43 to 2.29). This emphasises the importance of considering the type of pathogen in efficacy studies. Results comparing duration of handwashing for bacteria were inconclusive; there were no apparent differences in the magnitude of the summary LRVs between the time categories with data which both typically showed a >2 log_10_ reduction; there was no summary data available for the middle category, 20–30 s of handwashing.

### Soap alternatives

Overall, soap alternatives resulted in a >2 LRV for bacteria, but performed less well for viruses. For laboratory-based studies, the results of our meta-analysis showed that hand-rubbing with alcohol-based sanitisers resulted in a very high summary LRV (3.13, 95% CI 2.7 to 3.56) across all studies of bacteria, but a much lower summary LRV for viruses (1.86, 95% CI 1.37 to 2.35) and an even lower value when only non-enveloped viruses were examined (1.1, 95% CI 0.3 to 1.89). Alcohol-based hand sanitiser is the most commonly recommended alternative to soap and water for handwashing, but other options exist, including non-alcohol-based antiseptics, soap alternatives like ash and sand, and antimicrobial towels and wipes. Non-alcohol based antiseptics, soap alternatives and antiseptic/antimicrobial towels also resulted in a >2 LRV in bacteria, but did not generally achieve a >2 LRV for viruses (there was insufficient data to summarise for antiseptic towels and soap alternatives for viruses). Depending on the active ingredients and application, these products may be more or less effective at inactivating different organisms. For example, non-enveloped viruses (such as norovirus) have been shown to be more difficult to inactivate using alcohol-based sanitisers. More work is needed to investigate the efficacy of alcohol-based sanitisers against enveloped viruses, which are expected to be more susceptible to inactivation but did not have enough data in this review for us to generate a summary LRV. Summary results for non-alcohol-based antiseptics resulted in a >2 LRV for both bacteria and viruses, suggesting that a number of antiseptic formulations are efficacious.

Data on soap alternatives and wipes also resulted in a >2 summary LRV for bacteria, but with few data points and highly heterogeneous hygiene materials, these summary results should be treated with scepticism. More research investigating the efficacy of alternatives to soap and water on a range of organisms is needed to make confident recommendations. Soap alternatives studied included hygiene materials around which there are discordant recommendations among handwashing guidelines such as the use of ash and sand, which are recommended for use when soap is not available in 22% of handwashing guidelines but advised against in 6% of guidelines globally.^7^ Results from the studies included in this review showed that ash and sand were both efficacious for handwashing in some conditions; however, there were only two studies identified each for ash and sand. While they may not leave hands looking or feeling cleaner, more research on sand and ash may be particularly useful to inform whether these are effective against organisms in resource-constrained areas.

### Hand drying

There was little consistency in results for hand drying, and most studies lacked the information required to generate a summary log_10_ reduction value describing the impact of different drying materials. Overall, studies tended to report that paper towels were efficacious for reducing contamination and preventing recontamination, which is consistent with findings from a previous systematic review of hand drying materials.^52^ However, both that review and this one found that there is no clear advantage to paper towels over other hand drying materials, and that there was substantial disagreement among studies. Part of this may be a result of different levels of moisture remaining on hands during sampling, as wet hands are more likely to retain bacteria.

### Field-based studies

Field-based studies showed substantially lower log_10_ reduction values than laboratory studies, which is expected when hands are naturally contaminated and have a smaller range of possible measurements. It is challenging to produce studies on handwashing effectiveness in the field because a high level of contamination beyond what is normally present is needed to quantify organisms on hands both before and after washing, especially if researchers are hoping to demonstrate a >2 log_10_ reduction in these organisms. These data are also difficult to draw direct comparisons from, as there are many factors that vary when handwashing in a real-world setting. Nonetheless, more handwashing data from field settings is needed to demonstrate which factors most impact effectiveness when handwashing is practiced in context.

### Strengths and limitations

This review was part of an integrated protocol for multiple related reviews which included an exhaustive search strategy encompassing multiple databases and grey literature sources and a two-phased approach to identify relevant literature of hand hygiene in community settings. Yet despite this rigorous approach and the substantial amount of data included in this review, there are also some key limitations. Data in this review is primarily from laboratory-based studies that approximate real world conditions but may not perform the same in reality, limiting conclusions we could make related to field studies. More work to assess the likely effectiveness of these methods when used in real-world situations can help provide more evidence for recommendations. Given the sparseness of this data, the inclusion of laboratory studies as an intended focus of the paper provides quantitative comparisons in efficacy across methods, and thus the most robust data on handwashing methods that are used in community settings and is a strength of the approach. Studies published in languages other than English were excluded to ensure that the study team was able to extract and interpret data correctly; however, we note that potentially relevant research from studies published in other languages was therefore not able to be considered, which could impact the strength of the evidence or result in potential bias in this review.

The results are highly heterogeneous within all categories, and conclusions for categories based on relatively few studies and data points should be considered carefully. Results from different studies, and even the same study, may belong in the same meta-analysis, but could use different laboratory methods, evaluate different hand hygiene materials (eg, different antiseptics) or organisms (eg, gram-positive and gram-negative bacteria), and report substantially different effect sizes. More data is needed to make comparisons of efficacy across different handwashing materials, given the heterogeneity within each category. This is especially true for some of the less commonly studied types of pathogens, such as enveloped viruses, and less commonly studied handwashing hand hygiene materials, such as soap alternatives. Data on handwashing with water only, handwashing for shorter than recommended durations and with water of poor microbial quality was limited. Funnel plots suggested evidence of potential publication bias. However, it is worth noting that funnel plot asymmetry is common when between-study heterogeneity is high. In addition, small-study effects for these data may not be as severe compared with other meta-analyses, as studies in this field often report results from multiple methods, and authors may feel less pressure not to publish when interventions are not impactful against certain pathogens as this is often an interesting result.

## Conclusion

This systematic review describes current evidence on handwashing efficacy for the removal and inactivation of organisms in community settings, and it demonstrates that while recommended handwashing methods with water and soap are well described for the removal of bacteria, there are significant gaps in the data on efficacy of alternative hygiene materials and the effectiveness of handwashing conditions that are common in real-world community settings. In general, handwashing with soap and water was efficacious against bacteria and viruses, and alcohol-based sanitisers were efficacious against bacteria, but the data for efficacy against viruses and using other hand hygiene materials and methods is limited and less certain. To formulate strong recommendations for handwashing methods, particularly considering viral pandemic illnesses and community resource restrictions, further research that describes the efficacy and effectiveness of a wider range of methods is critical.

## Supplementary material

10.1136/bmjgh-2025-018925 online supplemental file 1

10.1136/bmjgh-2025-018925 online supplemental file 2

10.1136/bmjgh-2025-018925 online supplemental file 3

10.1136/bmjgh-2025-018925 online supplemental file 4

10.1136/bmjgh-2025-018925 online supplemental file 5

10.1136/bmjgh-2025-018925 online supplemental file 6

10.1136/bmjgh-2025-018925 online supplemental file 7

10.1136/bmjgh-2025-018925 online supplemental file 8

10.1136/bmjgh-2025-018925 online supplemental file 9

10.1136/bmjgh-2025-018925 online supplemental file 10

10.1136/bmjgh-2025-018925 online supplemental file 11

## Data Availability

Data are available in a public, open access repository.
