# Supplementary figures and images for: Efficacy and effectiveness of hand hygiene-related practices used in community settings for removal of organisms from hands: a systematic review
Source: BMJ Glob Health. 2025 Sep 16;10(Suppl 7):e018925. doi: 10.1136/bmjgh-2025-018925 (PMC12443168; doi:10.1136/bmjgh-2025-018925)

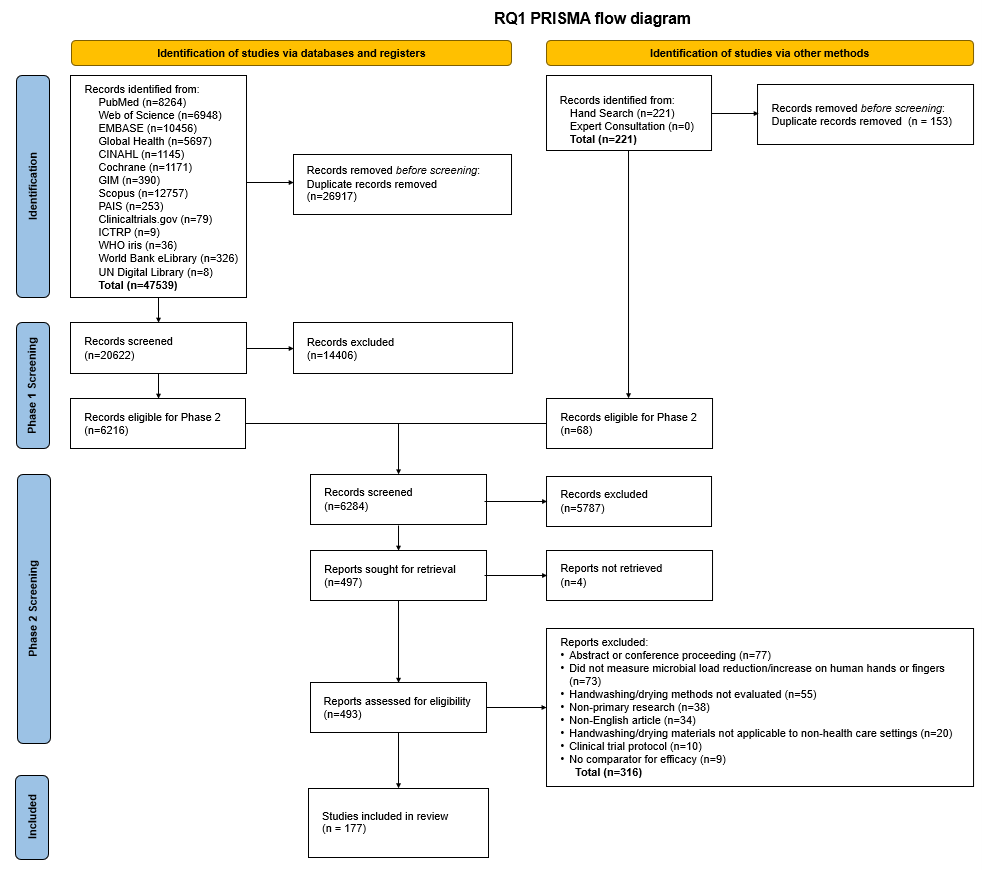

Supplement: online supplemental file 2 [file bmjgh-10-Suppl_7-s002.png]
