# Supplementary material for: Efficacy and effectiveness of hand hygiene-related practices used in community settings for removal of organisms from hands: a systematic review
Source: BMJ Glob Health. 2025 Sep 16;10(Suppl 7):e018925. doi: 10.1136/bmjgh-2025-018925 (PMC12443168; doi:10.1136/bmjgh-2025-018925)
Supplement: online supplemental file 3 [file bmjgh-10-Suppl_7-s003.docx]

# Extraction fields for RQ1

| **1. Information about the study** | | | | |
| --- | --- | --- | --- | --- |
| 1.1 | Publication type |  | *Select one*  (1) Journal article  (2) Grey literature (e.g., unpublished academic papers [e.g., thesis], non-peer reviewed papers, research and committee reports, government reports, conference papers/abstract, ongoing research)  (777) Other - specify |  |
| 1.3 | Registered trial | Does the study report that it is linked to a registered trial? (e.g., clinicaltrials.gov, ICTRP) | *Select one*  (1) Yes  (0) No |  |
| 1.4 | Trial # | If linked to a registered trial, paste trial registration number | Text  (999) Not applicable |  |
| **2. Eligibility** | | | | |
| 2.1 | Confirm participants | Check each of the following participants that the study includes.  DO NOT PROCEED IF NONE ARE CHECKED | *Check multiple*  (1) General populations in community settings  (2) Laboratory-based studies on interventions used in community settings | [Phase 2 criteria](https://emory-my.sharepoint.com/:w:/r/personal/bcaruso_emory_edu/Documents/Research/WHO/WHO-HW%20Systematic%20Reviews/1.%20WHO%20HW%20Systematic%20Reviews%20-%20project%20folder/3.%20Research%20activities/(3)%20Training%20documents/Phase%202%20training%20materials/Phase%202%20eligibility%20criteria.docx?d=w93c15eb4a94847adbb31c7248d059553&csf=1&web=1&e=Qc1U2s) |
| 2.2 | Confirm Intervention | Check each of the following interventions that the study includes.  DO NOT PROCEED IF NONE ARE CHECKED | *Check multiple*  (1) Handwashing with soap and water methods for varying durations  (2) Other handwashing materials including antiseptics, friction-generating materials, and water alone for varying durations  (3) Any hand drying method after handwashing with water or soap and water  (4) Hand washing with soap and water contaminated with microorganisms (naturally or experimentally) |  |
| 2.3 | Confirm Comparison | Check each of the following comparisons that the study includes.  DO NOT PROCEED IF NONE ARE CHECKED | *Check multiple*  (1) Handwashing with water alone for varying duration  (2) Handwashing with soap and water  (3) Air drying without assistance (4) Hand washing with soap and clean water |  |
| 2.4 | Confirm Outcome | Check each of the following outcomes that the study includes.  DO NOT PROCEED IF NONE ARE CHECKED | *Check multiple*  (1) Microbial load reduction in key pathogens and their surrogates on human hands or fingers from before to after washing  (2) Microbial load increase in key pathogens and their surrogates on human hands or fingers after washing but before drying and after drying |  |
| 2.5 | Confirm study design | Check each of the following outcomes that the study includes.  DO NOT PROCEED IF NONE ARE CHECKED | *Select one*  (1) Field efficacy study  (2) Laboratory efficacy study |  |
| **3. Setting** | | | | |
| 3.1 | Country | Which country is represented in the study? (List all countries separated by a comma, if study is from multiple sites) | *Text*  999 = Not applicable |  |
| 3.2 | WHO region | Which WHO region is represented in the study?  Select all that apply  Reference list of WHO regions [here.](https://en.wikipedia.org/wiki/List_of_WHO_regions) | *Check multiple*  (1) African Region (AFR)  (2) Region of Americas (AMR)  (3) South-East Asian Region (SEAR)  (4) European Region (EUR)  (5) Eastern Mediterranean Region (EMR)  (6) Western Pacific Region (WPR)  (888) Not reported | WHO reporting requirement |
| **4. Methods** | | | | |
| 4.1 | Aim of study | Paste the aim/objective/purpose/goal as stated in the full text article for the study | *Text*  (888) Unclear |  |
| 4.2 | Start date | What is the study start date? Month, Year | *Text*  (888) Not reported |  |
| **5. Participants** | | | | |
| 5.1 | Study population | What is the group of people that researchers are examining in the study? Select all that apply | *Check multiple*  (1) General population  (2) Adults (Women and Men)  (3) Adults (Women only)  (4) Adults (Men only)  (5) Children (Girls and Boys)  (6) Children (Girls only)  (7) Children (Boys only)  (8) Mother-child dyads  (9) Food workers  (10) Non-food occupational workers  (777) Other – specify  (888) Not reported |  |
| 5.2 | Vulnerable/specific populations | Does study concern any of the following vulnerable populations?  Select all that apply | *Check multiple*  (1) Individuals with specific illness or risk factors  (2) Specific ethnic or religious groups  (3) Persons experiencing homelessness  (4) Persons with disabilities  (5) Immigrants and migrants  (6) Refugees and displaced persons  (7) Elderly  (8) Pregnant women  (777) Other – specify  (999) None of the above |  |
| 5.3 | Number of participants | What is the total number of participants/sample size?    If the study does not stratify participants by age or gender, leave those fields blank | *Table*  2 x 3 table for Adult/Child vs Female/Male/Total |  |
| 5.4 | Age group | What is the age range of study participants? If not reported, put "888" in the "Adult participants x Lower age" cell only | *Table*  2x2 table for Adult/child vs Lower age/Upper age |  |
| **6. Hand Hygiene** | | | | |
| 6.1 | Practice | What is the hand hygiene practice that is being assessed? Select all that apply | *Check multiple*  (1) Handwashing with soap and water  (2) Handwashing with water  (3) Hand rubbing with alcohol-based hand rub  (4) Handwashing with non-alcoholic antiseptics  (5) Handwashing with soap alternatives (e.g., ash)  (6) Hand rubbing with towel (or other friction-generating)  (7) Hand drying (passive air dry)  (8) Hand drying (towel dry)  (9) Hand drying (blow dry)  (777) Other – specify  (888) Not reported |  |
| **7. Handwashing/Drying Conditions (RQ1)** | | | | |
| 7.1 | Method | What microbial sampling method(s) were used? | *Check multiple*  (1) Whole hand rinse with rubbing (ex: Glove juice)  (2) Whole hand rinse without rubbing  (3) Fingertip rinse with rubbing  (4) Fingertip rinse without rubbing  (5) Imprint whole hand  (6) Imprint fingers  (7) Swab whole hand  (8) Swab fingertip  (9) Vial inversion  (10) Glass Bead  (11) Other  (888) Not reported | Montville & Schaffner (Hand search list) |
| **8. Key Outcomes (RQ1)** | | | | |
| 8.1 | Key Findings | Briefly describe the key findings from the study, focusing on microbial load reductions or increases and comparisons between conditions. (Can copy and paste sentences/phrases from the paper) | *Text*  (888) Not reported |  |
| 8.2 | Comments | Please note here any deviations from the data extraction form. | *Text*  (999) Not Applicable |  |
| **9. Outcome Table (**See Excel table that we will use for populating Outcome Table instead of in Covidence (more readable) : [Outcome Table Template](https://emory-my.sharepoint.com/:x:/r/personal/bcaruso_emory_edu/Documents/Research/WHO/WHO-HW%20Systematic%20Reviews/1.%20WHO%20HW%20Systematic%20Reviews%20-%20project%20folder/4.%20Deliverables/(5)%20Data%20extraction/(1)%20Work%20Package%201%20-%20Micro/RQ1%20Data%20Extraction/Outcome%20Table%20Template.xlsx?d=wa03dfe511e6b48a5b0b3ae45eed1bf6a&csf=1&web=1&e=RS7Dyl)) | | | | |

# Bias assessment

| **Bias Assessment** | | | | |
| --- | --- | --- | --- | --- |
| MMAT (All articles) | | | | |
| S1 | Screening question 1 (for all types) | Are there clear research questions? | (0) No  (1) Yes  (888) Can’t tell | [MMAT User Guide](http://mixedmethodsappraisaltoolpublic.pbworks.com/w/file/fetch/127916259/MMAT_2018_criteria-manual_2018-08-01_ENG.pdf) |
| S2 | Screening question 2 (for all types) | Do the collected data allow to address the research questions? | (0) No  (1) Yes  (888) Can’t tell | [MMAT User Guide](http://mixedmethodsappraisaltoolpublic.pbworks.com/w/file/fetch/127916259/MMAT_2018_criteria-manual_2018-08-01_ENG.pdf) |
| Quantitative randomized controlled trials | | | | |
| 1.1 | Is randomization appropriately performed? | | (0) No  (1) Yes  (888) Can’t tell  (999) Not applicable | [MMAT User Guide](http://mixedmethodsappraisaltoolpublic.pbworks.com/w/file/fetch/127916259/MMAT_2018_criteria-manual_2018-08-01_ENG.pdf) |
| 1.2 | Are the groups comparable at baseline? | | (0) No  (1) Yes  (888) Can’t tell  (999) Not applicable | [MMAT User Guide](http://mixedmethodsappraisaltoolpublic.pbworks.com/w/file/fetch/127916259/MMAT_2018_criteria-manual_2018-08-01_ENG.pdf) |
| 1.3 | Are there complete outcome data? (complete is defined as data for >90% of participants) | | (0) No  (1) Yes  (888) Can’t tell  (999) Not applicable | [MMAT User Guide](http://mixedmethodsappraisaltoolpublic.pbworks.com/w/file/fetch/127916259/MMAT_2018_criteria-manual_2018-08-01_ENG.pdf) |
| 1.4 | Are outcome assessors blinded to the intervention provided? | | (0) No  (1) Yes  (888) Can’t tell  (999) Not applicable | [MMAT User Guide](http://mixedmethodsappraisaltoolpublic.pbworks.com/w/file/fetch/127916259/MMAT_2018_criteria-manual_2018-08-01_ENG.pdf) |
| 1.5 | Did the participants adhere to the assigned intervention? | | (0) No  (1) Yes  (888) Can’t tell  (999) Not applicable | [MMAT User Guide](http://mixedmethodsappraisaltoolpublic.pbworks.com/w/file/fetch/127916259/MMAT_2018_criteria-manual_2018-08-01_ENG.pdf) |
| Quantitative non-randomized | | | | |
| 2.1 | Are the participants representative of the target population? | | (0) No  (1) Yes  (888) Can’t tell  (999) Not applicable | [MMAT User Guide](http://mixedmethodsappraisaltoolpublic.pbworks.com/w/file/fetch/127916259/MMAT_2018_criteria-manual_2018-08-01_ENG.pdf) |
| 2.2 | Are measurements appropriate regarding both the outcome and intervention (or exposure)? | | (0) No  (1) Yes  (888) Can’t tell  (999) Not applicable | [MMAT User Guide](http://mixedmethodsappraisaltoolpublic.pbworks.com/w/file/fetch/127916259/MMAT_2018_criteria-manual_2018-08-01_ENG.pdf) |
| 2.3 | Are there complete outcome data? | | (0) No  (1) Yes  (888) Can’t tell  (999) Not applicable | [MMAT User Guide](http://mixedmethodsappraisaltoolpublic.pbworks.com/w/file/fetch/127916259/MMAT_2018_criteria-manual_2018-08-01_ENG.pdf) |
| 2.4 | Are the confounders accounted for in the design and analysis? | | (0) No  (1) Yes  (888) Can’t tell  (999) Not applicable | [MMAT User Guide](http://mixedmethodsappraisaltoolpublic.pbworks.com/w/file/fetch/127916259/MMAT_2018_criteria-manual_2018-08-01_ENG.pdf) |
| 2.5 | During the study period, is the intervention administered (or exposure occurred) as intended? | | (0) No  (1) Yes  (888) Can’t tell  (999) Not applicable | [MMAT User Guide](http://mixedmethodsappraisaltoolpublic.pbworks.com/w/file/fetch/127916259/MMAT_2018_criteria-manual_2018-08-01_ENG.pdf) |
| **Laboratory Study Bias Assessment** | | | | |
| 3.1 | Adequate controls | Are there adequate controls? | (0) Fail  (1) Pass | [Yeargin et al. (2016)](https://www-sciencedirect-com.proxy.library.emory.edu/science/article/pii/S0196655316002765#s0010) |
| 3.2 | Methods clearly described | Are the methods clearly described? | (0) Fail  (1) Pass | [Yeargin et al. (2016)](https://www-sciencedirect-com.proxy.library.emory.edu/science/article/pii/S0196655316002765#s0010) |
| 3.3 | Appropriate detection method | Was an appropriate detection method used? | (0) Fail  (1) Pass | [Yeargin et al. (2016)](https://www-sciencedirect-com.proxy.library.emory.edu/science/article/pii/S0196655316002765#s0010) |
| 3.4 | Studies performed at least in duplicate | Was the study performed at least in duplicate? | (0) Fail  (1) Pass | [Yeargin et al. (2016)](https://www-sciencedirect-com.proxy.library.emory.edu/science/article/pii/S0196655316002765#s0010) |
| 3.5 | Statistical analysis of data | Was statistical analysis of data performed? | (0) Fail  (1) Pass | [Yeargin et al. (2016)](https://www-sciencedirect-com.proxy.library.emory.edu/science/article/pii/S0196655316002765#s0010) |
