# Supplementary material for: Efficacy and effectiveness of hand hygiene-related practices used in community settings for removal of organisms from hands: a systematic review
Source: BMJ Glob Health. 2025 Sep 16;10(Suppl 7):e018925. doi: 10.1136/bmjgh-2025-018925 (PMC12443168; doi:10.1136/bmjgh-2025-018925)
Supplement: online supplemental file 5 [file bmjgh-10-Suppl_7-s005.docx]

S5: Bibliography of the 177 studies evaluated in the meta-analysis.

1. Aihara Y, Sakamoto I, Kondo N, Shrestha S, Kazama F. Handwashing and microbial contamination on the palms of preschool children in Kathmandu, Nepal. Kokusai Hoken Iryo (Journal of International Health). 2014;29(2):69–74.

2. Alsagher MR, Soudah SA, Khsheba AE, Fadel SM, Dadiesh MA, Houme MA, et al. Hand washing before and after applying different hand hygiene techniques in places of public concern in Tripoli-Libya. Open Microbiology Journal. 2018;12(1):364–75.

3. Ameri S, Nasrollahi SA, Samadi A, Amiri F, Ahmadvand S, Yadangi S, et al. Assessment of skin microbiota and biometric parameters: a comprehensive comparison of four types of hand cleansers. Iranian Journal of Dermatology. 2021;24(4):306–14.

4. Amin N, Pickering AJ, Ram PK, Unicomb L, Najnin N, Homaira N, et al. Microbiological evaluation of the efficacy of soapy water to clean hands: A randomized, non-inferiority field trial. American Journal of Tropical Medicine and Hygiene. 2014;91(2):415–23.

5. Anderson CE, Tong J, Zambrana W, Boehm AB, Wolfe MK. Investigating the Efficacy of Various Handwashing Methods against Enveloped and Non-Enveloped Viruses. American Journal of Tropical Medicine and Hygiene. 2023;108(4):820–8.

6. Ansari SA, Sattar SA, Springthorpe VS, Wells GA, Tostowaryk W. In vivo protocol for testing efficacy of hand-washing agents against viruses and bacteria: Experiments with rotavirus and Escherichia coli. Applied and Environmental Microbiology. 1989;55(12):3113–8.

7. Ansari SA, Springthorpe VS, Sattar SA, Tostowaryk W, Wells GA. Comparison of cloth, paper, and warm air drying in eliminating viruses and bacteria from washed hands. AJIC: American Journal of Infection Control. 1991;19(5):243–9.

8. Appelgrein C, Hosgood G, Dunn AL, Schaaf O. Ozonated water is inferior to propanol-based hand rubs for disinfecting hands. Journal of Hospital Infection. 2016;92(4):340–3.

9. Arbogast JW, Bowersock L, Parker AJ, Macinga DR. Randomized controlled trial evaluating the antimicrobial efficacy of chlorhexidine gluconate and para-chloro-meta-xylenol handwash formulations in real-world doses. American Journal of Infection Control. 2019;47(6):726–8.

10. Ayliffe GAJ, Babb JR, Davies JG, Lilly HA. Hand disinfection: a comparison of various agents in laboratory and ward studies. Journal of Hospital Infection. 1988;11(3):226–43.

11. Ayliffe GAJ, Babb JR, Davies JG, Newsom SWB, Rowland C, Platt JH, et al. Hygienic hand disinfection tests in three laboratories. Journal of Hospital Infection. 1990;16(2):141–9.

12. Babeluk R, Jutz S, Mertlitz S, Matiasek J, Klaus C. Hand hygiene - Evaluation of three disinfectant hand sanitizers in a community setting. PLoS ONE. 2014;9(11).

13. Bartzokas CA, Corkill JE, Makin T. Evaluation of the skin disinfecting activity and cumulative effect of chlorhexidine and triclosan handwash preparations on hands artificially contaminated with Serratia marcescens. Infection Control. 1987;8(4):163–7.

14. Bartzokas CA, Gibson MF, Graham R, Pinder DC. A comparison of triclosan and chlorhexidine preparations with 60 per cent isopropyl alcohol for hygienic hand disinfection. Journal of Hospital Infection. 1983;4(3):245–55.

15. Bellamy K, Alcock R, Babb JR, Davies JG, Ayliffe GAJ. A test for the assessment of “hygienic” hand disinfection using rotavirus. Journal of Hospital Infection. 1993;24(3):201–10.

16. Bettin K, Clabots C, Mathie P, Willard K, Gerding DN. Effectiveness of liquid soap vs. Chlorhexidine gluconate for the removal of clostridium difficile from bare hands and gloved hands. Infection Control &amp; Hospital Epidemiology. 1994;15(11):697–702.

17. Blackmore MA. A comparison of hand drying methods. Catering and Health. 1989;1:189–98.

18. Borges LF d A e, Silva BL, Gontijo Filho PP. Hand washing: Changes in the skin flora. American Journal of Infection Control. 2007;35(6):417–20.

19. Breidablik HJ, Johannessen L, Andersen JR, Søreide H, Kleiven OT. Effect of Optimal Alcohol-Based Hand Rub among Nurse Students Compared with Everyday Practice among Random Adults; Can Water-Based Hand Rub Combined with a Hand Dryer Machine Be an Alternative to Remove E. coli Contamination from Hands? Microorganisms. 2023;11(2).

20. Breidablik HJ, Lysebo DE, Johannessen L, Skare Å, Andersen JR, Kleiven OT. Ozonized water as an alternative to alcohol-based hand disinfection. Journal of Hospital Infection. 2019;102(4):419–24.

21. Breidablik HJ, Lysebo DE, Johannessen L, Skare Å, Andersen JR, Kleiven O. Effects of hand disinfection with alcohol hand rub, ozonized water, or soap and water: time for reconsideration? Journal of Hospital Infection. 2020;105(2):213–5.

22. Brown JM; A. Survey of consumer attitudes and the effectiveness of hand cleansers in the home. Food Protection Trends. 2007;27(8):603–11.

23. Cardoso CL, Pereira HH, Zequim JC, Guilhermetti M. Effectiveness of hand-cleansing agents for removing Acinetobacter baumannii strain from contaminated hands. American Journal of Infection Control. 1999;27(4):327–31.

24. Casewell MW, Law MM, Desai N. A laboratory model for testing agents for hygienic hand disinfection: handwashing and chlorhexidine for the removal of klebsiella. Journal of Hospital Infection. 1988;12(3):163–75.

25. Chang SC, Li WC, Huang KY, Huang YC, Chiu CH, Chen CJ, et al. Efficacy of alcohols and alcohol-based hand disinfectants against human enterovirus 71. Journal of Hospital Infection. 2013;83(4):288–93.

26. Clark YNB, Jimenez M, Raso E, Antwi L, Ofosu-Appiah LH, Opare D, et al. Evaluation of key antimicrobial properties of Moringa oleifera in relation to its use as a hand-washing product. Water (Switzerland). 2018;10(9).

27. Conover DM, Gibson KE. Comparison of two plain soap types for removal of bacteria and viruses from hands with specific focus on food service environments. Food Control. 2016;69:141–6.

28. Courtenay M, Ramirez L, Cox B, Han I, Jiang X, Dawson P. Effects of various hand hygiene regimes on removal and/or destruction of Escherichia coli on hands. Food Service Technology. 2005;5(2–4):77–84.

29. D’Antonio NN, Rihs JD, Stout JE, Yu VL. Revisiting the hand wipe versus gel rub debate: Is a higher-ethanol content hand wipe more effective than an ethanol gel rub? American Journal of Infection Control. 2010;38(9):678–82.

30. Dan K, Katoh N, Matsuoka T, Fujinami K. In vitro Antimicrobial Effects of Virus Block, Which Contains Multiple Polyoxometalate Compounds, and Hygienic Effects of Virus Block-Supplemented Moist Hand Towels. Pharmacology. 2019;104(1–2):98–112.

31. Davies JG, Babb JR, Bradley CR, Ayliffe GAJ. Preliminary study of test methods to assess the virucidal activity of skin disinfectants using poliovirus and bacteriophages. Journal of Hospital Infection. 1993;25(2):125–31.

32. Davis MA, Sheng H, Newman J, Hancock DD, Hovde CJ. Comparison of a waterless hand-hygiene preparation and soap-and-water hand washing to reduce coliforms on hands in animal exhibit settings. Epidemiology and Infection. 2006;134(5):1024–8.

33. De Aceituno AF, Bartz FE, Hodge DW, Shumaker DJ, Grubb JE, Arbogast JW, et al. Ability of hand hygiene interventions using alcohol-based hand sanitizers and soap to reduce microbial load on farmworker hands soiled during harvest. Journal of Food Protection. 2015;78(11):2024–32.

34. De Wit JC, Kampelmacher EH. Some Aspects of bacterial contamination of hands of workers in food service establishments. Zentralblatt fur Bakteriologie Mikrobiologie und Hygiene - Abt 1 Orig B Hygiene. 1988;186(1):45–54.

35. Deschênes P, Chano F, Dionne LL, Pittet D, Longtin Y. Efficacy of the World Health Organization–recommended handwashing technique and a modified washing technique to remove Clostridium difficile from hands. American Journal of Infection Control. 2017;45(8):844–8.

36. Devamani C, Schmidt WP. A simple microbiological tool to evaluate the effect of environmental health interventions on hand contamination. International Journal of Environmental Research and Public Health. 2014;11(11):11846–59.

37. Dharan S, Hugonnet S, Sax H, Pittet D. Evaluation of interference of a hand care cream with alcohol-based hand disinfection. Dermatologie in Beruf und Umwelt. 2001;49(1 A):81–4.

38. Dixon N, Morgan M, Equils O. Foam soap is not as effective as liquid soap in eliminating hand microbial flora. American Journal of Infection Control. 2017;45(7):813–4.

39. do Prado MF, Coelho ACC, de Brito JPB, Ferreira DO, Junior AW, da Silva Menecucci C, et al. Antimicrobial efficacy of alcohol-based hand gels with a 30-s application. Letters in Applied Microbiology. 2012;54(6):564–7.

40. Edmonds SL, MacInga DR, Mays-Suko P, Duley C, Rutter J, Jarvis WR, et al. Comparative efficacy of commercially available alcohol-based hand rubs and World Health Organization-recommended hand rubs: Formulation matters. American Journal of Infection Control. 2012;40(6):521–5.

41. Edmonds SL, Mann J, McCormack RR, MacInga DR, Fricker CM, Arbogast JW, et al. SaniTwice: A novel approach to hand hygiene for reducing bacterial contamination on hands when soap and water are unavailable. Journal of Food Protection. 2010;73(12):2296–300.

42. Edmonds SL, McCormack RR, Zhou SS, Macinga DR, Fricker CM. Hand hygiene regimens for the reduction of risk in food service environments. Journal of Food Protection. 2012;75(7):1303–9.

43. Edmonds SL, Zapka C, Kasper D, Gerber R, McCormack R, Macinga D, et al. Effectiveness of hand hygiene for removal of Clostridium difficile spores from hands. Infection Control and Hospital Epidemiology. 2013;34(3):302–5.

44. Eggers HJ. Experiments on Antiviral Activity of Hand Disinfectants. Some Theoretical and Practical Considerations. Zentralblatt fur Bakteriologie. 1990;273(1):36–51.

45. Eggers M, Benzinger C, Suchomel M, Hjorth E. Virucidal activity of three ethanol-based hand rubs against murine norovirus in a hand hygiene clinical simulation study. Future Microbiology. 2020;15(14):1335–41.

46. Eggers M, Koburger-Janssen T, Ward LS, Newby C, Müller S. Bactericidal and Virucidal Activity of Povidone-Iodine and Chlorhexidine Gluconate Cleansers in an In Vivo Hand Hygiene Clinical Simulation Study. Infectious Diseases and Therapy. 2018;7(2):235–47.

47. Escudero-Abarca BI, Goulter RM, Manuel CS, Leslie RA, Green K, Arbogast JW, et al. Comparative Assessment of the Efficacy of Commercial Hand Sanitizers Against Human Norovirus Evaluated by an in vivo Fingerpad Method. Frontiers in Microbiology. 2022;13.

48. Fischler GE, Fuls JL, Dail EW, Duran MH, Rodgers ND, Waggoner AL. Effect of hand wash agents on controlling the transmission of pathogenic bacteria from hands to food. Journal of Food Protection. 2007;70(12):2873–7.

49. Friedrich MND, Julian TR, Kappler A, Nhiwatiwa T, Mosler HJ. Handwashing, but how? Microbial effectiveness of existing handwashing practices in high-density suburbs of Harare, Zimbabwe. American Journal of Infection Control. 2017;45(3):228–33.

50. Fuls JL, Rodgers ND, Fischler GE, Howard JM, Patel M, Weidner PL, et al. Alternative hand contamination technique to compare the activities of antimicrobial and nonantimicrobial soaps under different test conditions. Applied and Environmental Microbiology. 2008;74(12):3739–44.

51. Geraldo IM, Gilman A, Shintre MS, Modak SM. Rapid antibacterial activity of 2 novel hand soaps: Evaluation of the risk of development of bacterial resistance to the antibacterial agents. Infection Control and Hospital Epidemiology. 2008;29(8):736–41.

52. Gill CO, McGinnis JC. Microbiological effects of hand washing at a beef carcass-breaking facility. Journal of Food Protection. 2003;66(3):493–6.

53. Gizaw Z, Yalew AW, Bitew BD, Lee J, Bisesi M. Effects of local handwashing agents on microbial contamination of the hands in a rural setting in Northwest Ethiopia: a cluster randomised controlled trial. BMJ open. 2022;12(5):e056411.

54. Gnatta JR, de Brito Poveda V, Padoveze MC, Graziano KU, Turrini RNT, da Silva MJP. Melaleuca alternifolia essential oil soap: a potential alternative for hand hygiene. European Journal of Clinical Microbiology and Infectious Diseases. 2021;40(7):1517–20.

55. Gnatta JR, Pinto FMG, Bruna CQM, de Souza RQ, Graziano KU, da Silva MJP. Comparison of hand hygiene antimicrobial efficacy: Melaleuca alternifolia essential oil versus triclosan. Revista Latino-Americana de Enfermagem. 2013;21(6):1212–9.

56. Goroncy-Bermes P. Hand disinfection according to the European Standard EN 1500 (hygienic handrub): A study with Gram-negative and Gram-positive test organisms. International Journal of Hygiene and Environmental Health. 2001;204(2–3):123–6.

57. Goroncy-Bermes P, Koburger T, Meyer B. Impact of the amount of hand rub applied in hygienic hand disinfection on the reduction of microbial counts on hands. Journal of Hospital Infection. 2010;74(3):212–8.

58. Grove SF, Suriyanarayanan A, Puli B, Zhao H, Li M, Li D, et al. Norovirus cross-contamination during preparation of fresh produce. International Journal of Food Microbiology. 2015;198:43–9.

59. Guilhermetti M, Hernandes SED, Fukushigue Y, Garcia LB, Cardoso CL. Effectiveness of hand-cleansing agents for removing methicillin-resistant Staphylococcus aureus from contaminated hands. Infection Control and Hospital Epidemiology. 2001;22(2):105–8.

60. Guilhermetti M, Marques Wiirzler LA, Castanheira Facio B, da Silva Furlan M, Campo Meschial W, Bronharo Tognim MC, et al. Antimicrobial efficacy of alcohol-based hand gels. Journal of Hospital Infection. 2010;74(3):219–24.

61. Gustafson DR, Vetter EA, Arson DRL, Ilstrup DM, Maker MD, Thompson RL, et al. Effects of 4 hand-drying methods for removing bacteria from washed hands: A randomized trial. Mayo Clinic Proceedings. 2000;75(7):705–8.

62. Heeg P. Does hand care ruin hand disinfection? Journal of Hospital Infection. 2001;48(SUPPL. A):S37–9.

63. Hitomi S, Baba S, Yano H, Morisawa Y, Kimura S. Antimicrobial effects of electrolytic products of sodium chloride--comparative evaluation with sodium hypochlorite solution and efficacy in handwashing. Kansenshogaku zasshi The Journal of the Japanese Association for Infectious Diseases. 1998;72(11):1176–81.

64. Huang Y, Oie S, Kamiya A. Comparative effectiveness of hand-cleansing agents for removing methicillin-resistant Staphylococcus aureus from experimentally contaminated fingertips. American Journal of Infection Control. 1994;22(4):224–7.

65. Jabbar U, Leischner J, Kasper D, Gerber R, Sambol SP, Parada JP, et al. Effectiveness of alcohol-based hand rubs for removal of Clostridium difficile spores from hands. Infection Control and Hospital Epidemiology. 2010;31(6):565–70.

66. Jensen DA, Danyluk MD, Harris LJ, Schaffner DW. Quantifying the effect of hand wash duration, soap use, ground beef debris, and drying methods on the removal of Enterobacter aerogenes on hands. Journal of Food Protection. 2015;78(4):685–90.

67. Jensen DA, MacInga DR, Shumaker DJ, Bellino R, Arbogast JW, Schaffner DW. Quantifying the effects of water temperature, soap volume, lather time, and antimicrobial soap as variables in the removal of Escherichia coli atcc 11229 from hands. Journal of Food Protection. 2017;80(6):1022–31.

68. Jimenez M, Siller JH, Valdez JB, Carrillo A, Chaidez C. Bidirectional Salmonella enterica serovar Typhimurium transfer between bare/glove hands and green bell pepper and its interruption. International Journal of Environmental Health Research. 2007;17(5):381–8.

69. Kampf G. How effective are hand antiseptics for the postcontamination treatment of hands when used as recommended? American Journal of Infection Control. 2008;36(5):356–60.

70. Kampf G, Grotheer D, Steinmann J. Efficacy of three ethanol-based hand rubs against feline calicivirus, a surrogate virus for norovirus. Journal of Hospital Infection. 2005;60(2):144–9.

71. Kampf G, Meyer B, Goroncy-Bermes P. Comparison of two test methods for the determination of sufficient antimicrobial activity of three commonly used alcohol-based hand rubs for hygienic hand disinfection. Journal of Hospital Infection. 2003;55(3):220–5.

72. Kampf G, Ostermeyer C. Intra-laboratory reproducibility of the hand hygiene reference procedures of EN 1499 (hygienic handwash) and EN 1500 (hygienic hand disinfection). Journal of Hospital Infection. 2002;52(3):219–24.

73. Kampf G, Ruselack S, Eggerstedt S, Nowak N, Bashir M. Less and less-influence of volume on hand coverage and bactericidal efficacy in hand disinfection. BMC Infectious Diseases. 2013;13(1).

74. Kampf G, Shaffer M, Hunte C. Insufficient neutralization in testing a chlorhexidine-containing ethanol-based hand rub can result in a false positive efficacy assessment. BMC Infectious Diseases. 2005;5.

75. Kasapoğlu S, Parlak-Yetişen L, Özdemir A, Dikmen D. Assessment of the effect of hand dryers used in shopping malls on hand hygiene. American Journal of Infection Control. 2022;50(10):1098–102.

76. Kawagoe JY, Graziano KU, Valle Martino MD, Siqueira I, Correa L. Bacterial reduction of alcohol-based liquid and gel products on hands soiled with blood. American Journal of Infection Control. 2011;39(9):785–7.

77. Kim SA, Moon H, Lee K, Rhee MS. Bactericidal effects of triclosan in soap both in vitro and in vivo. The Journal of antimicrobial chemotherapy. 2015;70(12):3345–52.

78. Koller W, Rotter ML, Gottardi W. Do “chlorine covers” exert a sustained bactericidal effect on the bacterial hand flora? Journal of Hospital Infection. 1995;31(3):169–76.

79. Kramer A, Galabov AS, Sattar SA, Döhner L, Pivert A, Payan C, et al. Virucidal activity of a new hand disinfectant with reduced ethanol content: Comparison with other alcohol-based formulations. Journal of Hospital Infection. 2006;62(1):98–106.

80. Kuraeiad S, Prueksatrakun P, Chuajeen Y, Kooltheat N, Sookbampen O, Mitsuwan W, et al. Evaluation of moisturizing property and antimicrobial activity of alcohol-based hand sanitizer formulations using coconut oil as a moisturizing agent against Staphylococcus aureus and Escherichia coli. Veterinary Integrative Sciences. 2022;20(2):419–30.

81. Lages SLS, Ramakrishnan MA, Goyal SM. In-vivo efficacy of hand sanitisers against feline calicivirus: a surrogate for norovirus. Journal of Hospital Infection. 2008;68(2):159–63.

82. Larson E, Aiello A, Lee LV, Della-Latta P, Gomez-Duarte C, Lin S. Short- and long-term effects of handwashing with antimicrobial or plain soap in the community. Journal of Community Health. 2003;28(2):139–50.

83. Larson E, Bobo L. Effective hand degerming in the presence of blood. Journal of Emergency Medicine. 1992;10(1):7–11.

84. Larson EL, Cohen B, Baxter KA. Analysis of alcohol-based hand sanitizer delivery systems: Efficacy of foam, gel, and wipes against influenza A (H1N1) virus on hands. American Journal of Infection Control. 2012;40(9):806–9.

85. Larson EL, Eke PI, Laughon BE. Efficacy of alcohol-based hand rinses under frequent-use conditions. Antimicrobial Agents and Chemotherapy. 1986;30(4):542–4.

86. Larson EL, Laughon BE. Comparison of four antiseptic products containing chlorhexidine gluconate. Antimicrobial Agents and Chemotherapy. 1987;31(10):1572–4.

87. Lee MG, Hunt P, Felix D. A comparison of two bactericidal handwashing agents containing chlorhexidine. Journal of Hospital Infection. 1988;12(1):59–63.

88. Liu P, Macinga DR, Fernandez ML, Zapka C, Hsiao HM, Berger B, et al. Comparison of the Activity of Alcohol-Based Handrubs Against Human Noroviruses Using the Fingerpad Method and Quantitative Real-Time PCR. Food and Environmental Virology. 2011;3(1):35–42.

89. Macinga DR, Edmonds SL, Campbell E, Shumaker DJ, Arbogast JW. Efficacy of novel alcohol-based hand rub products at typical in-use volumes. Infection Control and Hospital Epidemiology. 2013;34(3):299–301.

90. Macinga DR, Sattar SA, Jaykus LA, Arbogast JW. Improved inactivation of nonenveloped enteric viruses and their surrogates by a novel alcohol-based hand sanitizer. Applied and Environmental Microbiology. 2008;74(16):5047–52.

91. Macinga DR, Shumaker DJ, Werner HP, Edmonds SL, Leslie RA, Parker AE, et al. The relative influences of product volume, delivery format and alcohol concentration on dry-time and efficacy of alcohol-based hand rubs. BMC Infectious Diseases. 2014;14(1).

92. Mackintosh CA, Hoffman PN. An extended model for transfer of micro-organisms via the hands: Differences between organisms and the effect of alcohol disinfection. Journal of Hygiene. 1984;92(3):345–55.

93. Mbithi JN, Springthorpe VS, Sattar SA. Comparative in vivo efficiencies of hand-washing agents against hepatitis A virus (HM-175) and poliovirus type 1 (Sabin). Applied and Environmental Microbiology. 1993;59(10):3463–9.

94. Messager S, Goddard PA, Dettmar PW, Maillard JY. Comparison of two in vivo and two ex vivo tests to assess the antibacterial activity of several antiseptics. Journal of Hospital Infection. 2004;58(2):115–21.

95. Messager S, Hammer KA, Carson CF, Riley TV. Effectiveness of hand-cleansing formulations containing tea tree oil assessed ex vivo on human skin and in vivo with volunteers using European standard EN 1499. Journal of Hospital Infection. 2005;59(3):220–8.

96. Michaels B, Gangar V, Lin CM, Doyle M. Use limitations of alcoholic instant hand sanitizer as part of a food service hand hygiene program. Food Service Technology. 2003;3(2):71–80.

97. Michaels B, Gangar V, Schultz A, Arenas M, Curiale M, Ayers T, et al. Water temperature as a factor in handwashing efficacy. Food Service Technology. 2002;2(3):139–49.

98. Miller ML; JD. A field study evaluating the effectiveness of different hand soaps and sanitizers. Dairy, food and environmental sanitation. 1994;14(3):155–60.

99. Morrison Jr AJ, Gratz J, Cabezudo I, Wenzel RP. The efficacy of several new handwashing agents for removing non-transient bacterial flora from hands. Infection Control. 1986;7(5):268–72.

100. Munyendo WLL, Kiprop AK. Design, preparation and evaluation of germicidal Toddalia asiatica herbal antiseptic detergent. Journal of Applied Pharmaceutical Science. 2016;6(11):100–4.

101. Myklebust S. Comparative antibacterial effectiveness of seven hand antiseptics. European Journal of Oral Sciences. 1985;93(6):546–54.

102. Myklebust S. Soap pH and the effectiveness of alcoholic hand antiseptics. European Journal of Oral Sciences. 1989;97(5):451–5.

103. Nakamura K, Hara Y, Harada R, Tanno D, Kashiwazaki J, Kobari S, et al. Evaluation of the antimicrobial effectiveness of ozonated water for hand washing in the presence of organic material contamination using the ASTM E2946-13 standard test method. Journal of Food Protection. 2021;84(11):1922–4.

104. Namura S, Nishijima S, Asada Y. An evaluation of the residual activity of antiseptic handrub lotions: An “in use” setting study. Journal of Dermatology. 1994;21(7):481–5.

105. Namura S, Nishijima S, McGinley KJ, Leyden JJ. A study of the efficacy of antimicrobial detergents for hand washing: Using the full-hand touch plates method. Journal of Dermatology. 1993;20(2):88–93.

106. Namura S, Nishijima S, Mitsuya K, Asada Y. Study of the efficacy of antiseptic handrub lotions with hand washing machines. Journal of Dermatology. 1994;21(6):405–10.

107. Nerandzic MM, Rackaityte E, Jury LA, Eckart K, Donskey CJ. Novel Strategies for Enhanced Removal of Persistent Bacillus anthracis Surrogates and Clostridium difficile Spores from Skin. PLoS ONE. 2013;8(7).

108. Nerandzic MM, Sunkesula VCK, Sankar C T, Setlow P, Donskey CJ. Unlocking the sporicidal potential of ethanol: Induced sporicidal activity of ethanol against clostridium difficile and bacillus spores under altered physical and chemical conditions. PLoS ONE. 2015;10(7).

109. Nhung DTT, Freydiere AM, Constant H, Falson F, Pirot F. Sustained antibacterial effect of a hand rub gel incorporating chlorhexdine-loaded nanocapsules (Nanochlorex®). International Journal of Pharmaceutics. 2007;334(1–2):166–72.

110. Nicoletti G, Boghossian V, Borland R. Hygienic hand disinfection: a comparative study with chlorhexidine detergents and soap. Journal of Hospital Infection. 1990;15(4):323–37.

111. Noskin GA, Stosor V Fau - Cooper I, Cooper I Fau - Peterson LR, Peterson LR. Recovery of vancomycin-resistant enterococci on fingertips and environmental surfaces. (0899-823X (Print)).

112. Ochwoto M, Muita L, Talaam K, Wanjala C, Ogeto F, Wachira F, et al. Anti-bacterial efficacy of alcoholic hand rubs in the kenyan market, 2015. Antimicrobial Resistance and Infection Control. 2017;6(1).

113. Oo KN, Win CY, Thida M. Effect of various cleansing materials on decontamination of hands. Myanmar Health Sciences Research Journal. 1997;9(1):33–6.

114. Ooi JP, Zarim NA, Lim V. Citrus aurontifolia and Cymbopogan flexuosus against Staphylococcus aureus and Escherichia coli. Malaysian Journal of Medicine and Health Sciences. 2019;15:37–42.

115. Ory J, Zingg W, De Kraker MEA, Soule H, Pittet D. Wiping is inferior to rubbing: A note of caution for hand hygiene with alcohol-based solutions. Infection Control and Hospital Epidemiology. 2018;39(3):332–5.

116. Oughton MT, Loo VG, Dendukuri N, Fenn S, Libman MD. Hand hygiene with soap and water is superior to alcohol rub and antiseptic wipes for removal of Clostridium difficile. Infection Control and Hospital Epidemiology. 2009;30(10):939–44.

117. Patnayak DP, Prasad M, Malik YS, Ramakrishnan MA, Goyal SM. Efficacy of disinfectants and hand sanitizers against avian respiratory viruses. Avian Diseases. 2008;52(2):199–202.

118. Paula H, Hübner NO, Assadian O, Bransmöller K, Baguhl R, Löffler H, et al. Effect of hand lotion on the effectiveness of hygienic hand antisepsis: Implications for practicing hand hygiene. American Journal of Infection Control. 2017;45(8):835–8.

119. Paulson DS, Riccardi C, Beausoleil CM, Fendler EJ, Dolan MJ, Dunkerton LV, et al. EFFICACY EVALUATION OF FOUR HAND CLEANSING REGIMENS FOR FOOD HANDLERS. Dairy, food and environmental sanitation. 1999;19:680–4.

120. Perez-Garza J, Garcia S, Heredia N. Removal of Escherichia coli and enterococcus faecalis after hand washing with antimicrobial and nonantimicrobial soap and persistence of these bacteria in rinsates. Journal of Food Protection. 2017;80(10):1670–5.

121. Pickering AJ, Boehm AB, Mwanjali M, Davis J. Efficacy of waterless hand hygiene compared with handwashing with soap: A field study in Dar es Salaam, Tanzania. American Journal of Tropical Medicine and Hygiene. 2010;82(2):270–8.

122. Pickering AJ, Davis J, Boehm AB. Efficacy of alcohol-based hand sanitizer on hands soiled with dirt and cooking oil. Journal of Water and Health. 2011;9(3):429–33.

123. Pires D, Soule H, Bellissimo-Rodrigues F, Gayet-Ageron A, Pittet D. Hand hygiene with alcohol-based hand rub: How long is long enough? Infection Control and Hospital Epidemiology. 2017;38(5):547–52.

124. Pires D, Soule H, Bellissimo-Rodrigues F, de Kraker MEA, Pittet D. Antibacterial efficacy of handrubbing for 15 versus 30 seconds: EN 1500-based randomized experimental study with different loads of Staphylococcus aureus and Escherichia coli. Clinical Microbiology and Infection. 2019;25(7):851–6.

125. Pitt SJ, Crockett SL, Andreou GM. The contribution of hand drying in prevention of transmission of microorganisms: Comparison of the efficacy of three hand drying methods in the removal and distribution of microorganisms. Journal of Infection Prevention. 2018;19(6):310–7.

126. Pitt SJ, Crockett SL, Andreou GM. The contribution of hand drying in prevention of transmission of microorganisms: Comparison of the efficacy of three hand drying methods in the removal and distribution of microorganisms. J Infect Prev. 2018 Nov;19(6):310–7.

127. Puthucheary SD, Thong ML, Parasakthi N. Evaluation of some hand washing and disinfection methods in the removal of transient bacterial flora. The Malaysian journal of pathology. 1981;4:49–55.

128. Racicot M, Kocher A, Beauchamp G, Letellier A, Vaillancourt JP. Assessing most practical and effective protocols to sanitize hands of poultry catching crew members. Preventive Veterinary Medicine. 2013;111(1–2):92–9.

129. Reynolds WO, Neas B, Kabara JJ, Flournoy DJ. Evaluation of a new germicidal hand creme. Methods and Findings in Experimental and Clinical Pharmacology. 1985;7(1):49–54.

130. Robinson AL, Lee HJ, Kwon J, Todd E, Rodriguez FP, Ryu D. Adequate hand washing and glove use are necessary to reduce cross-contamination from hands with high bacterial loads. Journal of Food Protection. 2016;79(2):304–8.

131. Rotter M, Koller W, Wewalka G. Povidone-iodine and chlorhexidine gluconate-containing detergents for disinfection of hands. Journal of Hospital Infection. 1980;1(2):149–58.

132. Rotter ML. Hygienic hand disinfection. Infection Control. 1984;5(1):18–22.

133. Rotter ML, Koller W. Test models for hygienic handrub and hygienic handwash: the effects of two different contamination and sampling techniques. Journal of Hospital Infection. 1992;20(3):163–71.

134. Rotter ML, Koller W, Neumann R. The influence of cosmetic additives on the acceptability of alcohol-based hand disinfectants. Journal of Hospital Infection. 1991;18(SUPPL. B):57–63.

135. Saad AH, Nag S, Bazigha R, Samour R, Rasool BKA. FORMULATION AND EVALUATION OF HERBAL HAND WASH FROM MATRICARIA CHAMOMILLA FLOWERS EXTRACTS. In 2011.

136. Sasahara T, Hayashi S, Hosoda K, Morisawa Y, Hirai Y. Comparison of hand hygiene procedures for removing Bacillus cereus spores. Biocontrol Science. 2014;19(3):129–34.

137. Sattar SA, Abebe M, Bueti AJ, Jampani H, Newman J, Hua S. Activity of an alcohol-based hand gel against human adeno-, rhino-, and rotaviruses using the fingerpad method. Infection Control and Hospital Epidemiology. 2000;21(8):516–9.

138. Sattar SA, Ali M, Tetro JA. In Vivo comparison of two human norovirus surrogates for testing ethanol-based handrubs: The mouse chasing the cat! PLoS ONE. 2011;6(2).

139. Schaffner DW, Bowman JP, English DJ, Fischler GE, Fuls JL, Krowka JF, et al. Quantitative microbial risk assessment of antibacterial hand hygiene products on risk of shigellosis. Journal of Food Protection. 2014;77(4):574–82.

140. Schaffner DW, Schaffner KM. Management of risk of microbial cross-contamination from uncooked frozen hamburgers by alcohol-based hand sanitizer. Journal of Food Protection. 2007;70(1):109–13.

141. Schlicher M. RCT OF THE EFFICACY OF SILVER NANOPARTICLE GEL AGAINST BACTERIAL HAND FLORA. 2009.

142. Schürmann W, Eggers HJ. An experimental study on the epidemiology of enteroviruses: water and soap washing of poliovirus 1 - contaminated hands, its effectiveness and kinetics. Medical Microbiology and Immunology. 1985;174(5):221–36.

143. Selk SH, Pogany SA, Higuchi T. Comparative antimicrobial activity, in vitro and in vivo, of soft N-chloramine systems and chlorhexidine. Applied and Environmental Microbiology. 1982;43(4):899–904.

144. Sharp K, Haysom I, Parkinson R. Anti-microbial hand washes for domestic use - Their effectiveness in vitro and in normal use K. Sharp et al. Anti-microbial hand washes for domestic use. International Journal of Consumer Studies. 2001;25(3):200–7.

145. Sheena AZ, Stiles ME. Immediate and Residual (Substantive) Efficacy of Germicidal Hand Wash Agents. (1944-9097 (Electronic)).

146. Sheena AZ, Stiles ME. Efficacy of Germicidal Hand Wash Agents in Hygienic Hand Disinfection1. Journal of Food Protection. 1982 Jun 1;45(8):713–20.

147. Sheena AZ, Stiles ME. Efficacy of Germicidal Hand Wash Agents Against Transient Bacteria Inoculated onto Hands. Journal of Food Protection. 1983 Aug 1;46(8):722–7.

148. Sickbert-Bennett EE, Weber DJ, Gergen-Teague MF, Sobsey MD, Samsa GP, Rutala WA. Comparative efficacy of hand hygiene agents in the reduction of bacteria and viruses. American Journal of Infection Control. 2005;33(2):67–77.

149. Snelling AM, Saville T, Stevens D, Beggs CB. Comparative evaluation of the hygienic efficacy of an ultra-rapid hand dryer vs conventional warm air hand dryers. Journal of Applied Microbiology. 2011;110(1):19–26.

150. Steinmann J, Nehrkorn R, Meyer A, Becker K. Two in-vivo protocols for testing virucidal efficacy of handwashing and hand disinfection. Zentralblatt für Hygiene und Umweltmedizin = International journal of hygiene and environmental medicine. 1995;196(5):425–36.

151. Steinmann J, Paulmann D, Becker B, Bischoff B, Steinmann E, Steinmann J. Comparison of virucidal activity of alcohol-based hand sanitizers versus antimicrobial hand soaps in vitro and in vivo. Journal of Hospital Infection. 2012;82(4):277–80.

152. Stiles ME, Sheena AZ. Efficacy of low-concentration iodophors for germicidal hand washing. Journal of Hygiene. 1985;94(3):269–77.

153. Stiles ME, Sheena AZ. Efficacy of Germicidal Hand Wash Agents in Use in a Meat Processing Plant. Journal of Food Protection. 1987 Apr 1;50(4):289–95.

154. Suchomel M, Fritsch F, Kampf G. Bactericidal efficacy of two modified WHO-recommended alcohol-based hand rubs using two types of rub-in techniques for 15 s. Journal of Hospital Infection. 2021;111:47–9.

155. Suchomel M, Gebel J, Kampf G. Failure of sodium hypochlorite to meet the EN 1500 efficacy requirement for hygienic hand disinfection. Journal of Hospital Infection. 2023;133:46–8.

156. Suen LKP, Lung VYT, Boost MV, Au-Yeung CH, Siu GKH. Microbiological evaluation of different hand drying methods for removing bacteria from washed hands. Scientific Reports. 2019;9(1).

157. Tambekar DH; S. Role of hand washing and factors for reducing transmission of enteric infections among students of Amravati district. Science Research Reporter. 2013;3(2):175–82.

158. Tamimi AH, Edmonds-Wilson SL, Gerba CP. Use of a Hand Sanitizing Wipe for Reducing Risk of Viral Illness in the Home. Food and Environmental Virology. 2015;7(4):354–8.

159. Tan JBX, de Kraker MEA, Pires D, Soule H, Pittet D. Handrubbing with sprayed alcohol-based hand rub: an alternative method for effective hand hygiene. Journal of Hospital Infection. 2020;104(4):430–4.

160. Taylor JH, Brown KL, Toivenen J, Holah JT. A microbiological evaluation of warm air hand driers with respect to hand hygiene and the washroom environment. J Appl Microbiol. 2000 Dec;89(6):910–9.

161. Torondel B, Khan R, Larsen TH, White S. Efficacy of the Supertowel®: An Alternative Hand-washing Product for Humanitarian Emergencies. American Journal of Tropical Medicine and Hygiene. 2019;100(5):1278–84.

162. Torondel B, Khan R, Larsen TH, White S. Evaluating the efficacy of the supertowel™ as a handwashing product: A simulation of real-world use conditions. American Journal of Tropical Medicine and Hygiene. 2021;104(4):1554–61.

163. Torondel B, Opare D, Brandberg B, Cobb E, Cairncross S. Efficacy of Moringa oleifera leaf powder as a hand- washing product: A crossover controlled study among healthy volunteers. BMC Complementary and Alternative Medicine. 2014;14.

164. Uttlová P, Urban J. HAND DISINFECTANTS AND THEIR ACTIVITY AGAINST CLINICAL ISOLATES OF BORDETELLA PERTUSSIS. Central European Journal of Public Health. 2022;30(4):230–4.

165. Vesley D, Lillquist DR, Le CT. Evaluation of nongermicidal handwashing protocols for removal of transient microbial flora. Applied and Environmental Microbiology. 1985;49(5):1067–71.

166. Weber DJ, Sickbert-Bennett E, Gergen MF, Rutala WA. Efficacy of Selected Hand Hygiene Agents Used to Remove Bacillus atrophaeus (a Surrogate of Bacillus anthracis) from Contaminated Hands. JAMA. 2003;289(10):1274–7.

167. Wilkinson MAC, Ormandy K, Bradley CR, Fraise AP, Hines J. Dose considerations for alcohol-based hand rubs. Journal of Hospital Infection. 2017;95(2):175–82.

168. Wilkinson MAC, Ormandy K, Bradley CR, Hines J. Comparison of the efficacy and drying times of liquid, gel and foam formats of alcohol-based hand rubs. Journal of Hospital Infection. 2018;98(4):359–64.

169. Wilson AM, Reynolds KA, Jaykus LA, Escudero-Abarca B, Gerba CP. Comparison of estimated norovirus infection risk reductions for a single fomite contact scenario with residual and nonresidual hand sanitizers. American Journal of Infection Control. 2020;48(5):538–44.

170. Wolfe MK, Gallandat K, Daniels K, Desmarais AM, Scheinman P, Lantagne D. Handwashing and Ebola virus disease outbreaks: A randomized comparison of soap, hand sanitizer, and 0.05% chlorine solutions on the inactivation and removal of model organisms Phi6 and E. coli from hands and persistence in rinse water. PLoS ONE. 2017;12(2).

171. Woolwine JD, Gerberding JL. Effect of testing method on apparent activities of antiviral disinfectants and antiseptics. Antimicrobial Agents and Chemotherapy. 1995;39(4):921–3.

172. Yamamoto Y, Ugai K, Takahashi Y. Efficiency of hand drying for removing bacteria from washed hands: Comparison of paper towel drying with warm air drying. Infection Control and Hospital Epidemiology. 2005;26(3):316–20.

173. Youn BH, Kim YS, Yoo S, Hur MH. Antimicrobial and hand hygiene effects of Tea Tree Essential Oil disinfectant: A randomised control trial. International Journal of Clinical Practice. 2021;75(8).

174. Zambrana W, Tong J, Anderson CE, Boehm AB, Wolfe MK. Quantifying the Viral Reduction Achieved Using Ash and Sand as Handwashing Agents. American Journal of Tropical Medicine and Hygiene. 2023;108(2):441–8.

175. Zapka CA, Campbell EJ, Maxwell SL, Gerba CP, Dolan MJ, Arbogast JW, et al. Bacterial hand contamination and transfer after use of contaminated bulk-soap-refillable dispensers. Applied and Environmental Microbiology. 2011;77(9):2898–904.

176. Zapka C, Leff J, Henley J, Tittl J, De Nardo E, Butler M, et al. Comparison of standard culture-based method to culture-independent method for evaluation of hygiene effects on the hand microbiome. mBio. 2017;8(2).

177. Zarpellon MN, Soares VS, Albrecht NR, Bergamasco DRDS, Garcia LB, Cardoso CL. Comparison of 3 alcohol gels and 70% ethyl alcohol for hand hygiene. Infection Control and Hospital Epidemiology. 2008;29(10):960–2.
