# Supplementary material for: Efficacy and effectiveness of hand hygiene-related practices used in community settings for removal of organisms from hands: a systematic review
Source: BMJ Glob Health. 2025 Sep 16;10(Suppl 7):e018925. doi: 10.1136/bmjgh-2025-018925 (PMC12443168; doi:10.1136/bmjgh-2025-018925)
Supplement: online supplemental file 7 [file bmjgh-10-Suppl_7-s007.docx]

S7 – Quality Appraisal of all Included Articles Using the Mixed Methods Appraisal Tool and a Laboratory Bias Assessment

| **Study** | **MMAT Score** | **Laboratory Bias Assessment Score** | **MMAT Quantitative Randomized Score** | **MMAT Quantitative Non-Randomized Score** | **Criteria from the Mixed Methods Appraisal Tool^1^** | | | | | | | | | | **Criteria from the Laboratory Bias Assessment^2^** | | | | |
| --- | --- | --- | --- | --- | --- | --- | --- | --- | --- | --- | --- | --- | --- | --- | --- | --- | --- | --- | --- |
|  |  |  |  |  | KEY Individual criteria scores can be either 0 (did not meet criteria) or 1 (met criteria); cells that are shaded in gray indicate that a criterion was not applicable to the study type.   Studies were assessed using the five-criteria questionnaire. Possible scores are 0–5 across study types (5 is the best).   † Indicates that the MMAT was deemed inappropriate for quality appraisal of the article. | | | | | | | | | | KEY Individual criteria scores can be either 0 ("Fail") or 1 ("Pass").  Studies were assessed using the five-criteria questionnaire. Possible scores are 0–5 (5 is the best). | | | | |
|  |  |  |  |  | **2.1** | **2.2** | **2.3** | **2.4** | **2.5** | **3.1** | **3.2** | **3.3** | **3.4** | **3.5** | **1** | **2** | **3** | **4** | **5** |
| Aihara 2014 | 5 | 5 |  | 5 |  |  |  |  |  | 1 | 1 | 1 | 1 | 1 | 1 | 1 | 1 | 1 | 1 |
| Alsagher 2018 | 3 | 5 | 3 |  | 1 | 0 | 1 | 0 | 1 |  |  |  |  |  | 1 | 1 | 1 | 1 | 1 |
| Ameri 2021 | 5 | 5 |  | 5 |  |  |  |  |  | 1 | 1 | 1 | 1 | 1 | 1 | 1 | 1 | 1 | 1 |
| Amin 2012 | 3 | 5 | 3 |  | 1 | 1 | 1 | 0 | 0 |  |  |  |  |  | 1 | 1 | 1 | 1 | 1 |
| Anderson 2023 | 4 | 5 | 4 |  | 1 | 1 | 1 | 0 | 1 |  |  |  |  |  | 1 | 1 | 1 | 1 | 1 |
| Ansari 1989 | 5 | 5 |  | 5 |  |  |  |  |  | 1 | 1 | 1 | 1 | 1 | 1 | 1 | 1 | 1 | 1 |
| Ansari 1991 | 4 | 3 |  | 4 |  |  |  |  |  | 0 | 1 | 1 | 1 | 1 | 0 | 1 | 1 | 0 | 1 |
| Appelgrein 2016 | 5 | 5 |  | 5 |  |  |  |  |  | 1 | 1 | 1 | 1 | 1 | 1 | 1 | 1 | 1 | 1 |
| Arbogast 2019 | 4 | 5 | 4 |  | 1 | 1 | 1 | 0 | 1 |  |  |  |  |  | 1 | 1 | 1 | 1 | 1 |
| Ayliffe 1988 | 5 | 5 |  | 5 |  |  |  |  |  | 1 | 1 | 1 | 1 | 1 | 1 | 1 | 1 | 1 | 1 |
| Ayliffe 1990 | 4 | 5 | 4 |  | 1 | 1 | 1 | 0 | 1 |  |  |  |  |  | 1 | 1 | 1 | 1 | 1 |
| Babeluk 2014 | 4 | 4 | 4 |  | 1 | 1 | 1 | 0 | 1 |  |  |  |  |  | 0 | 1 | 1 | 1 | 1 |
| Bartzokas 1983 | 4 | 5 |  | 4 |  |  |  |  |  | 0 | 1 | 1 | 1 | 1 | 1 | 1 | 1 | 1 | 1 |
| Bartzokas 1987 | 3 | 5 | 3 |  | 0 | 1 | 1 | 0 | 1 |  |  |  |  |  | 1 | 1 | 1 | 1 | 1 |
| Bellamy 1993 | 5 | 5 |  | 5 |  |  |  |  |  | 1 | 1 | 1 | 1 | 1 | 1 | 1 | 1 | 1 | 1 |
| Bettin 1994 | 5 | 5 |  | 5 |  |  |  |  |  | 1 | 1 | 1 | 1 | 1 | 1 | 1 | 1 | 1 | 1 |
| Blackmore 1989 | 4 | 5 |  | 4 |  |  |  |  |  | 0 | 1 | 1 | 1 | 1 | 1 | 1 | 1 | 1 | 1 |
| Borges 2007 | 5 | 5 |  | 5 |  |  |  |  |  | 1 | 1 | 1 | 1 | 1 | 1 | 1 | 1 | 1 | 1 |
| Breidablik 2019 | 4 | 5 | 4 |  | 1 | 1 | 1 | 0 | 1 |  |  |  |  |  | 1 | 1 | 1 | 1 | 1 |
| Breidablik 2020 | 5 | 5 |  | 5 |  |  |  |  |  | 1 | 1 | 1 | 1 | 1 | 1 | 1 | 1 | 1 | 1 |
| Breidablik 2023 | 5 | 5 |  | 5 |  |  |  |  |  | 1 | 1 | 1 | 1 | 1 | 1 | 1 | 1 | 1 | 1 |
| Brown 2007 | 5 | 5 |  | 5 |  |  |  |  |  | 1 | 1 | 1 | 1 | 1 | 1 | 1 | 1 | 1 | 1 |
| Cardoso 1999 | 4 | 5 | 4 |  | 1 | 1 | 1 | 0 | 1 |  |  |  |  |  | 1 | 1 | 1 | 1 | 1 |
| Casewell 1988 | 5 | 4 |  | 5 |  |  |  |  |  | 1 | 1 | 1 | 1 | 1 | 1 | 1 | 1 | 1 | 0 |
| Chang 2013 | 3 | 5 | 3 |  | 0 | 1 | 1 | 0 | 1 |  |  |  |  |  | 1 | 1 | 1 | 1 | 1 |
| Clark 2018 | 5 | 5 |  | 5 |  |  |  |  |  | 1 | 1 | 1 | 1 | 1 | 1 | 1 | 1 | 1 | 1 |
| Conover 2016 | 4 | 5 | 4 |  | 1 | 1 | 1 | 0 | 1 |  |  |  |  |  | 1 | 1 | 1 | 1 | 1 |
| Courtenay 2005 | 4 | 5 |  | 4 |  |  |  |  |  | 0 | 1 | 1 | 1 | 1 | 1 | 1 | 1 | 1 | 1 |
| Dan 2019 | 5 | 5 |  | 5 |  |  |  |  |  | 1 | 1 | 1 | 1 | 1 | 1 | 1 | 1 | 1 | 1 |
| D'Antonio 2010 | 5 | 5 |  | 5 |  |  |  |  |  | 1 | 1 | 1 | 1 | 1 | 1 | 1 | 1 | 1 | 1 |
| Davies 1993 | 4 | 5 |  | 4 |  |  |  |  |  | 0 | 1 | 1 | 1 | 1 | 1 | 1 | 1 | 1 | 1 |
| Davis 2006 | 4 | 5 | 4 |  | 1 | 1 | 1 | 0 | 1 |  |  |  |  |  | 1 | 1 | 1 | 1 | 1 |
| de Aceituno 2015 | 4 | 5 | 4 |  | 1 | 1 | 1 | 0 | 1 |  |  |  |  |  | 1 | 1 | 1 | 1 | 1 |
| de Wit 1988 | 4 | 3 |  | 4 |  |  |  |  |  | 1 | 1 | 1 | 0 | 1 | 1 | 0 | 1 | 1 | 0 |
| Deschênes 2017 | 3 | 5 | 3 |  | 0 | 1 | 1 | 0 | 1 |  |  |  |  |  | 1 | 1 | 1 | 1 | 1 |
| Devamani 2014 | 4 | 5 | 4 |  | 1 | 1 | 1 | 0 | 1 |  |  |  |  |  | 1 | 1 | 1 | 1 | 1 |
| Dharan 2001 | 4 | 5 |  | 4 |  |  |  |  |  | 1 | 1 | 1 | 0 | 1 | 1 | 1 | 1 | 1 | 1 |
| Dixon 2017 | 4 | 5 | 4 |  | 1 | 1 | 1 | 0 | 1 |  |  |  |  |  | 1 | 1 | 1 | 1 | 1 |
| do Prado 2012 | 4 | 5 |  | 4 |  |  |  |  |  | 0 | 1 | 1 | 1 | 1 | 1 | 1 | 1 | 1 | 1 |
| Edmonds 2010 | 4 | 5 | 4 |  | 1 | 1 | 1 | 0 | 1 |  |  |  |  |  | 1 | 1 | 1 | 1 | 1 |
| Edmonds 2012 | 4 | 5 | 4 |  | 1 | 1 | 1 | 0 | 1 |  |  |  |  |  | 1 | 1 | 1 | 1 | 1 |
| Edmonds 2012 | 4 | 5 | 4 |  | 1 | 1 | 1 | 0 | 1 |  |  |  |  |  | 1 | 1 | 1 | 1 | 1 |
| Edmonds 2013 | 4 | 5 |  | 4 |  |  |  |  |  | 0 | 1 | 1 | 1 | 1 | 1 | 1 | 1 | 1 | 1 |
| Eggers 1990 | 3 | 4 |  | 3 |  |  |  |  |  | 0 | 1 | 1 | 0 | 1 | 1 | 1 | 1 | 1 | 0 |
| Eggers 2018 | 4 | 5 | 4 |  | 1 | 1 | 1 | 0 | 1 |  |  |  |  |  | 1 | 1 | 1 | 1 | 1 |
| Eggers 2020 | 4 | 5 | 4 |  | 1 | 1 | 1 | 0 | 1 |  |  |  |  |  | 1 | 1 | 1 | 1 | 1 |
| Escudero-Abarca 2022 | 5 | 5 |  | 5 |  |  |  |  |  | 1 | 1 | 1 | 1 | 1 | 1 | 1 | 1 | 1 | 1 |
| Fischler 2007 | 4 | 5 | 4 |  | 1 | 1 | 1 | 0 | 1 |  |  |  |  |  | 1 | 1 | 1 | 1 | 1 |
| Friedrich 2017 | 4 | 4 |  | 4 |  |  |  |  |  | 1 | 1 | 1 | 0 | 1 | 0 | 1 | 1 | 1 | 1 |
| Fuls 2008 | 4 | 5 | 4 |  | 1 | 1 | 1 | 0 | 1 |  |  |  |  |  | 1 | 1 | 1 | 1 | 1 |
| Geraldo 2008 | 4 | 3 |  | 4 |  |  |  |  |  | 1 | 1 | 1 | 0 | 1 | 1 | 0 | 0 | 1 | 1 |
| Gill 2003 | 4 | 5 |  | 4 |  |  |  |  |  | 1 | 1 | 1 | 0 | 1 | 1 | 1 | 1 | 1 | 1 |
| Gizaw 2022 | 5 | 5 | 5 |  | 1 | 1 | 1 | 1 | 1 |  |  |  |  |  | 1 | 1 | 1 | 1 | 1 |
| Gnatta 2013 | 5 | 5 | 5 |  | 1 | 1 | 1 | 1 | 1 |  |  |  |  |  | 1 | 1 | 1 | 1 | 1 |
| Gnatta 2021 | 4 | 5 | 4 |  | 1 | 1 | 1 | 0 | 1 |  |  |  |  |  | 1 | 1 | 1 | 1 | 1 |
| Goroncy-Bermes 2001 | 3 | 5 | 3 |  | 0 | 1 | 1 | 0 | 1 |  |  |  |  |  | 1 | 1 | 1 | 1 | 1 |
| Goroncy-Bermes 2010 | 5 | 5 |  | 5 |  |  |  |  |  | 1 | 1 | 1 | 1 | 1 | 1 | 1 | 1 | 1 | 1 |
| Grove 2015 | 4 | 5 |  | 4 |  |  |  |  |  | 0 | 1 | 1 | 1 | 1 | 1 | 1 | 1 | 1 | 1 |
| Guilhermetti 2001 | 4 | 5 | 4 |  | 1 | 1 | 1 | 0 | 1 |  |  |  |  |  | 1 | 1 | 1 | 1 | 1 |
| Guilhermetti 2010 | 5 | 5 |  | 5 |  |  |  |  |  | 1 | 1 | 1 | 1 | 1 | 1 | 1 | 1 | 1 | 1 |
| Gustafson 2000 | 4 | 5 | 4 |  | 1 | 1 | 1 | 0 | 1 |  |  |  |  |  | 1 | 1 | 1 | 1 | 1 |
| Hanna 1996 | 5 | 5 |  | 5 |  |  |  |  |  | 1 | 1 | 1 | 1 | 1 | 1 | 1 | 1 | 1 | 1 |
| Heeg 2001 | 3 | 4 |  | 3 |  |  |  |  |  | 0 | 1 | 1 | 0 | 1 | 1 | 0 | 1 | 1 | 1 |
| Hitomi 1998 | 4 | 5 |  | 4 |  |  |  |  |  | 0 | 1 | 1 | 1 | 1 | 1 | 1 | 1 | 1 | 1 |
| Huang 1994 | 4 | 5 | 4 |  | 1 | 1 | 1 | 0 | 1 |  |  |  |  |  | 1 | 1 | 1 | 1 | 1 |
| Jabbar 2010 | 4 | 5 |  | 4 |  |  |  |  |  | 0 | 1 | 1 | 1 | 1 | 1 | 1 | 1 | 1 | 1 |
| Jensen 2015 | 4 | 5 |  | 4 |  |  |  |  |  | 0 | 1 | 1 | 1 | 1 | 1 | 1 | 1 | 1 | 1 |
| Jensen 2017 | 4 | 5 | 4 |  | 1 | 1 | 1 | 0 | 1 |  |  |  |  |  | 1 | 1 | 1 | 1 | 1 |
| Jimenez 2007 | 5 | 5 |  | 5 |  |  |  |  |  | 1 | 1 | 1 | 1 | 1 | 1 | 1 | 1 | 1 | 1 |
| Kampf 2002 | 3 | 4 |  | 3 |  |  |  |  |  | 0 | 1 | 1 | 0 | 1 | 1 | 0 | 1 | 1 | 1 |
| Kampf 2003 | 4 | 5 |  | 4 |  |  |  |  |  | 1 | 1 | 1 | 0 | 1 | 1 | 1 | 1 | 1 | 1 |
| Kampf 2005 | 4 | 5 |  | 4 |  |  |  |  |  | 0 | 1 | 1 | 1 | 1 | 1 | 1 | 1 | 1 | 1 |
| Kampf 2005 | 3 | 5 |  | 3 |  |  |  |  |  | 0 | 1 | 1 | 0 | 1 | 1 | 1 | 1 | 1 | 1 |
| Kampf 2008 | 5 | 5 | 5 |  | 1 | 1 | 1 | 1 | 1 |  |  |  |  |  | 1 | 1 | 1 | 1 | 1 |
| Kampf 2013 | 4 | 5 | 4 |  | 1 | 1 | 1 | 0 | 1 |  |  |  |  |  | 1 | 1 | 1 | 1 | 1 |
| Kasapoğlu 2022 | 4 | 4 |  | 4 |  |  |  |  |  | 0 | 1 | 1 | 1 | 1 | 1 | 1 | 1 | 0 | 1 |
| Kawagoe 2011 | 5 | 4 | 5 |  | 1 | 1 | 1 | 1 | 1 |  |  |  |  |  | 0 | 1 | 1 | 1 | 1 |
| Kim 2015 | 5 | 5 |  | 5 |  |  |  |  |  | 1 | 1 | 1 | 1 | 1 | 1 | 1 | 1 | 1 | 1 |
| Koller 1995 | 4 | 5 |  | 4 |  |  |  |  |  | 0 | 1 | 1 | 1 | 1 | 1 | 1 | 1 | 1 | 1 |
| Kramer 2006 | 4 | 5 | 4 |  | 1 | 1 | 1 | 0 | 1 |  |  |  |  |  | 1 | 1 | 1 | 1 | 1 |
| Kuraeiad 2022 | 5 | 5 |  | 5 |  |  |  |  |  | 1 | 1 | 1 | 1 | 1 | 1 | 1 | 1 | 1 | 1 |
| Lages 2008 | 5 | 4 |  | 5 |  |  |  |  |  | 1 | 1 | 1 | 1 | 1 | 1 | 1 | 1 | 1 | 0 |
| Larson 1986 | 4 | 5 | 4 |  | 1 | 1 | 1 | 0 | 1 |  |  |  |  |  | 1 | 1 | 1 | 1 | 1 |
| Larson 1987 | 4 | 5 | 4 |  | 1 | 1 | 1 | 0 | 1 |  |  |  |  |  | 1 | 1 | 1 | 1 | 1 |
| Larson 1992 | 4 | 5 | 4 |  | 1 | 1 | 1 | 0 | 1 |  |  |  |  |  | 1 | 1 | 1 | 1 | 1 |
| Larson 2003 | 5 | 5 | 5 |  | 1 | 1 | 1 | 1 | 1 |  |  |  |  |  | 1 | 1 | 1 | 1 | 1 |
| Larson 2012 | 4 | 5 | 4 |  | 1 | 1 | 1 | 0 | 1 |  |  |  |  |  | 1 | 1 | 1 | 1 | 1 |
| Lee 1988 | 4 | 5 |  | 4 |  |  |  |  |  | 0 | 1 | 1 | 1 | 1 | 1 | 1 | 1 | 1 | 1 |
| Liu 2011 | 4 | 5 |  | 4 |  |  |  |  |  | 0 | 1 | 1 | 1 | 1 | 1 | 1 | 1 | 1 | 1 |
| Macinga 2008 | 2 | 5 | 2 |  | 0 | 1 | 0 | 0 | 1 |  |  |  |  |  | 1 | 1 | 1 | 1 | 1 |
| Macinga 2013 | 4 | 3 |  | 4 |  |  |  |  |  | 1 | 1 | 1 | 0 | 1 | 0 | 0 | 1 | 1 | 1 |
| Macinga 2014 | 4 | 5 | 4 |  | 1 | 1 | 1 | 0 | 1 |  |  |  |  |  | 1 | 1 | 1 | 1 | 1 |
| Mackintosh 1984 | 5 | 5 |  | 5 |  |  |  |  |  | 1 | 1 | 1 | 1 | 1 | 1 | 1 | 1 | 1 | 1 |
| Mbithi 1993 | 5 | 5 |  | 5 |  |  |  |  |  | 1 | 1 | 1 | 1 | 1 | 1 | 1 | 1 | 1 | 1 |
| Messager 2004 | 4 | 4 |  | 4 |  |  |  |  |  | 1 | 1 | 1 | 0 | 1 | 1 | 0 | 1 | 1 | 1 |
| Messager 2005 | 5 | 5 |  | 5 |  |  |  |  |  | 1 | 1 | 1 | 1 | 1 | 1 | 1 | 1 | 1 | 1 |
| Michaels 2002 | 5 | 5 | 5 |  | 1 | 1 | 1 | 1 | 1 |  |  |  |  |  | 1 | 1 | 1 | 1 | 1 |
| Michaels 2003 | 4 | 5 |  | 4 |  |  |  |  |  | 0 | 1 | 1 | 1 | 1 | 1 | 1 | 1 | 1 | 1 |
| Miller 1994 | 5 | 5 |  | 5 |  |  |  |  |  | 1 | 1 | 1 | 1 | 1 | 1 | 1 | 1 | 1 | 1 |
| MorrisonJr 1986 | 4 | 5 |  | 4 |  |  |  |  |  | 0 | 1 | 1 | 1 | 1 | 1 | 1 | 1 | 1 | 1 |
| Munyendo 2016 | 3 | 5 |  | 3 |  |  |  |  |  | 0 | 1 | 1 | 0 | 1 | 1 | 1 | 1 | 1 | 1 |
| Myklebust 1985 | 4 | 5 |  | 4 |  |  |  |  |  | 0 | 1 | 1 | 1 | 1 | 1 | 1 | 1 | 1 | 1 |
| Myklebust 1989 | 5 | 5 |  | 5 |  |  |  |  |  | 1 | 1 | 1 | 1 | 1 | 1 | 1 | 1 | 1 | 1 |
| Nakamura 2021 | 4 | 5 | 4 |  | 1 | 1 | 1 | 0 | 1 |  |  |  |  |  | 1 | 1 | 1 | 1 | 1 |
| Namura 1993 | 5 | 5 |  | 5 |  |  |  |  |  | 1 | 1 | 1 | 1 | 1 | 1 | 1 | 1 | 1 | 1 |
| Namura 1994 | 5 | 5 |  | 5 |  |  |  |  |  | 1 | 1 | 1 | 1 | 1 | 1 | 1 | 1 | 1 | 1 |
| Namura 1994 | 5 | 5 |  | 5 |  |  |  |  |  | 1 | 1 | 1 | 1 | 1 | 1 | 1 | 1 | 1 | 1 |
| Nerandzic 2013 | 4 | 5 | 4 |  | 1 | 1 | 1 | 0 | 1 |  |  |  |  |  | 1 | 1 | 1 | 1 | 1 |
| Nerandzic 2015 | 4 | 5 | 4 |  | 1 | 1 | 1 | 0 | 1 |  |  |  |  |  | 1 | 1 | 1 | 1 | 1 |
| Nhung 2007 | 4 | 5 |  | 4 |  |  |  |  |  | 0 | 1 | 1 | 1 | 1 | 1 | 1 | 1 | 1 | 1 |
| Nicoletti 1990 | 4 | 5 | 4 |  | 1 | 1 | 1 | 0 | 1 |  |  |  |  |  | 1 | 1 | 1 | 1 | 1 |
| Noskin 1995 | 3 | 4 |  | 3 |  |  |  |  |  | 0 | 1 | 1 | 0 | 1 | 1 | 1 | 1 | 1 | 0 |
| Ochwoto 2017 | 5 | 4 |  | 5 |  |  |  |  |  | 1 | 1 | 1 | 1 | 1 | 1 | 0 | 1 | 1 | 1 |
| Oo 1997 | 3 | 3 |  | 3 |  |  |  |  |  | 0 | 1 | 1 | 0 | 1 | 0 | 1 | 1 | 1 | 0 |
| Ooi 2019 | 4 | 5 |  | 4 |  |  |  |  |  | 0 | 1 | 1 | 1 | 1 | 1 | 1 | 1 | 1 | 1 |
| Ory 2018 | 4 | 4 | 4 |  | 1 | 1 | 1 | 0 | 1 |  |  |  |  |  | 1 | 0 | 1 | 1 | 1 |
| Oughton 2009 | 4 | 5 | 4 |  | 1 | 1 | 1 | 0 | 1 |  |  |  |  |  | 1 | 1 | 1 | 1 | 1 |
| Patnayak 2008 | 5 | 5 |  | 5 |  |  |  |  |  | 1 | 1 | 1 | 1 | 1 | 1 | 1 | 1 | 1 | 1 |
| Paula 2017 | 5 | 5 |  | 5 |  |  |  |  |  | 1 | 1 | 1 | 1 | 1 | 1 | 1 | 1 | 1 | 1 |
| Paulson 1999 | 4 | 5 |  | 4 |  |  |  |  |  | 0 | 1 | 1 | 1 | 1 | 1 | 1 | 1 | 1 | 1 |
| Perez-Garza 2017 | 3 | 5 |  | 3 |  |  |  |  |  | 0 | 1 | 0 | 1 | 1 | 1 | 1 | 1 | 1 | 1 |
| Pickering 2010 | 5 | 5 |  | 5 |  |  |  |  |  | 1 | 1 | 1 | 1 | 1 | 1 | 1 | 1 | 1 | 1 |
| Pickering 2011 | 4 | 5 | 4 |  | 1 | 1 | 1 | 0 | 1 |  |  |  |  |  | 1 | 1 | 1 | 1 | 1 |
| Pires 2017 | 4 | 5 | 4 |  | 1 | 1 | 1 | 0 | 1 |  |  |  |  |  | 1 | 1 | 1 | 1 | 1 |
| Pires 2019 | 5 | 4 | 5 |  | 1 | 1 | 1 | 1 | 1 |  |  |  |  |  | 0 | 1 | 1 | 1 | 1 |
| Pitt 2018 | 4 | 3 |  | 4 |  |  |  |  |  | 1 | 1 | 1 | 0 | 1 | 0 | 0 | 1 | 1 | 1 |
| Puthucheary 1981 | 3 | 4 |  | 3 |  |  |  |  |  | 0 | 1 | 1 | 0 | 1 | 1 | 1 | 1 | 1 | 0 |
| Racicot 2013 | 4 | 4 | 4 |  | 1 | 1 | 1 | 0 | 1 |  |  |  |  |  | 1 | 0 | 1 | 1 | 1 |
| Reynolds 1985 | 4 | 4 |  | 4 |  |  |  |  |  | 1 | 1 | 1 | 0 | 1 | 1 | 0 | 1 | 1 | 1 |
| Robinson 2016 | 5 | 5 |  | 5 |  |  |  |  |  | 1 | 1 | 1 | 1 | 1 | 1 | 1 | 1 | 1 | 1 |
| Rotter 1980 | 4 | 5 |  | 4 |  |  |  |  |  | 0 | 1 | 1 | 1 | 1 | 1 | 1 | 1 | 1 | 1 |
| Rotter 1984 | 3 | 5 |  | 3 |  |  |  |  |  | 0 | 1 | 1 | 0 | 1 | 1 | 1 | 1 | 1 | 1 |
| Rotter 1991 | 5 | 4 | 5 |  | 1 | 1 | 1 | 1 | 1 |  |  |  |  |  | 1 | 0 | 1 | 1 | 1 |
| Rotter 1992 | 4 | 5 | 4 |  | 1 | 1 | 1 | 0 | 1 |  |  |  |  |  | 1 | 1 | 1 | 1 | 1 |
| Saad 2011 | 4 | 5 |  | 4 |  |  |  |  |  | 0 | 1 | 1 | 1 | 1 | 1 | 1 | 1 | 1 | 1 |
| Sasahara 2014 | 5 | 5 |  | 5 |  |  |  |  |  | 1 | 1 | 1 | 1 | 1 | 1 | 1 | 1 | 1 | 1 |
| Sattar 2000 | 4 | 5 |  | 4 |  |  |  |  |  | 0 | 1 | 1 | 1 | 1 | 1 | 1 | 1 | 1 | 1 |
| Sattar 2011 | 4 | 5 |  | 4 |  |  |  |  |  | 0 | 1 | 1 | 1 | 1 | 1 | 1 | 1 | 1 | 1 |
| Schaffner 2007 | 5 | 5 |  | 5 |  |  |  |  |  | 1 | 1 | 1 | 1 | 1 | 1 | 1 | 1 | 1 | 1 |
| Schaffner 2014 | 4 | 5 | 4 |  | 0 | 1 | 1 | 1 | 1 |  |  |  |  |  | 1 | 1 | 1 | 1 | 1 |
| Schlicher 2009 | 5 | 5 | 5 |  | 1 | 1 | 1 | 1 | 1 |  |  |  |  |  | 1 | 1 | 1 | 1 | 1 |
| Schürmann 1985 | 5 | 5 |  | 5 |  |  |  |  |  | 1 | 1 | 1 | 1 | 1 | 1 | 1 | 1 | 1 | 1 |
| Selk 1982 | 4 | 5 |  | 4 |  |  |  |  |  | 0 | 1 | 1 | 1 | 1 | 1 | 1 | 1 | 1 | 1 |
| Sharp 2001 | 5 | 5 |  | 5 |  |  |  |  |  | 1 | 1 | 1 | 1 | 1 | 1 | 1 | 1 | 1 | 1 |
| Sheena 1982 | 4 | 5 | 4 |  | 1 | 1 | 1 | 0 | 1 |  |  |  |  |  | 1 | 1 | 1 | 1 | 1 |
| Sheena 1983 | 4 | 5 | 4 |  | 1 | 1 | 1 | 0 | 1 |  |  |  |  |  | 1 | 1 | 1 | 1 | 1 |
| Sheena 1983 | 4 | 5 | 4 |  | 1 | 1 | 1 | 0 | 1 |  |  |  |  |  | 1 | 1 | 1 | 1 | 1 |
| Sickbert-Bennett 2005 | 4 | 5 | 4 |  | 1 | 1 | 1 | 0 | 1 |  |  |  |  |  | 1 | 1 | 1 | 1 | 1 |
| Snelling 2011 | 4 | 5 | 4 |  | 1 | 1 | 1 | 0 | 1 |  |  |  |  |  | 1 | 1 | 1 | 1 | 1 |
| Steinmann 1995 | 5 | 5 |  | 5 |  |  |  |  |  | 1 | 1 | 1 | 1 | 1 | 1 | 1 | 1 | 1 | 1 |
| Steinmann 2012 | 5 | 5 |  | 5 |  |  |  |  |  | 1 | 1 | 1 | 1 | 1 | 1 | 1 | 1 | 1 | 1 |
| Stiles 1985 | 4 | 5 | 4 |  | 1 | 1 | 1 | 0 | 1 |  |  |  |  |  | 1 | 1 | 1 | 1 | 1 |
| Stiles 1987 | 5 | 4 |  | 5 |  |  |  |  |  | 1 | 1 | 1 | 1 | 1 | 1 | 0 | 1 | 1 | 1 |
| Suchomel 2021 | 4 | 5 | 4 |  | 1 | 1 | 1 | 0 | 1 |  |  |  |  |  | 1 | 1 | 1 | 1 | 1 |
| Suchomel 2023 | 3 | 5 |  | 3 |  |  |  |  |  | 0 | 1 | 1 | 0 | 1 | 1 | 1 | 1 | 1 | 1 |
| Suen 2019 | 4 | 5 | 4 |  | 1 | 1 | 1 | 0 | 1 |  |  |  |  |  | 1 | 1 | 1 | 1 | 1 |
| Tambekar 2013 | 4 | 4 |  | 4 |  |  |  |  |  | 1 | 1 | 1 | 0 | 1 | 1 | 0 | 1 | 1 | 1 |
| Tamimi 2015 | 3 | 3 |  | 3 |  |  |  |  |  | 0 | 1 | 1 | 0 | 1 | 1 | 0 | 1 | 1 | 0 |
| Tan 2020 | 4 | 5 | 4 |  | 1 | 1 | 1 | 0 | 1 |  |  |  |  |  | 1 | 1 | 1 | 1 | 1 |
| Taylor 2000 | 5 | 5 |  | 5 |  |  |  |  |  | 1 | 1 | 1 | 1 | 1 | 1 | 1 | 1 | 1 | 1 |
| Torondel 2014 | 5 | 5 |  | 5 |  |  |  |  |  | 1 | 1 | 1 | 1 | 1 | 1 | 1 | 1 | 1 | 1 |
| Torondel 2019 | 4 | 5 | 4 |  | 1 | 1 | 1 | 0 | 1 |  |  |  |  |  | 1 | 1 | 1 | 1 | 1 |
| Torondel 2021 | 4 | 5 | 4 |  | 1 | 1 | 1 | 0 | 1 |  |  |  |  |  | 1 | 1 | 1 | 1 | 1 |
| Uttlová 2022 | 5 | 5 |  | 5 |  |  |  |  |  | 1 | 1 | 1 | 1 | 1 | 1 | 1 | 1 | 1 | 1 |
| Vesley 1985 | 4 | 5 |  | 4 |  |  |  |  |  | 1 | 0 | 1 | 1 | 1 | 1 | 1 | 1 | 1 | 1 |
| Weber 2003 | 4 | 5 | 4 |  | 1 | 1 | 1 | 0 | 1 |  |  |  |  |  | 1 | 1 | 1 | 1 | 1 |
| Wilkinson 2017 | 5 | 5 |  | 5 |  |  |  |  |  | 1 | 1 | 1 | 1 | 1 | 1 | 1 | 1 | 1 | 1 |
| Wilkinson 2018 | 4 | 5 |  | 4 |  |  |  |  |  | 0 | 1 | 1 | 1 | 1 | 1 | 1 | 1 | 1 | 1 |
| Wilson 2020 | 5 | 5 |  | 5 |  |  |  |  |  | 1 | 1 | 1 | 1 | 1 | 1 | 1 | 1 | 1 | 1 |
| Wolfe 2017 | 4 | 5 | 4 |  | 1 | 1 | 1 | 0 | 1 |  |  |  |  |  | 1 | 1 | 1 | 1 | 1 |
| Woolwine 1995 | 3 | 5 |  | 3 |  |  |  |  |  | 0 | 1 | 1 | 0 | 1 | 1 | 1 | 1 | 1 | 1 |
| Yamamoto 2005 | 4 | 5 | 4 |  | 1 | 1 | 1 | 0 | 1 |  |  |  |  |  | 1 | 1 | 1 | 1 | 1 |
| Youn 2021 | 5 | 5 | 5 |  | 1 | 1 | 1 | 1 | 1 |  |  |  |  |  | 1 | 1 | 1 | 1 | 1 |
| Zambrana 2023 | 4 | 5 | 4 |  | 1 | 1 | 1 | 0 | 1 |  |  |  |  |  | 1 | 1 | 1 | 1 | 1 |
| Zapka 2011 | 4 | 5 |  | 4 |  |  |  |  |  | 1 | 1 | 1 | 0 | 1 | 1 | 1 | 1 | 1 | 1 |
| Zapka 2017 | 4 | 5 | 4 |  | 1 | 1 | 1 | 0 | 1 |  |  |  |  |  | 1 | 1 | 1 | 1 | 1 |
| Zarpellon 2008 | 3 | 5 | 3 |  | 0 | 1 | 1 | 0 | 1 |  |  |  |  |  | 1 | 1 | 1 | 1 | 1 |

^1^Hong QN, Pluye P, Fàbregues S, *et al.* Mixed methods appraisal tool (MMAT) version 2018: user guide. *Montr McGill Univ*. 2018;1.

^2^ Yeargin T, Buckley D, Fraser A, *et al.* The survival and inactivation of enteric viruses on soft surfaces: A systematic review of the literature. *Am J Infect Control*. 2016;44:1365–73. doi: 10.1016/j.ajic.2016.03.018

**Criteria from the MMAT:**

*Quantitative Randomized*

2.1 Is randomization appropriately performed?

2.2 Are the groups comparable at baseline?

2.3 Are there complete outcome data?

2.4 Are outcome assessors blinded to the intervention provided?

2.5 Did the participants adhere to the assigned intervention?

*Quantitative Non-Randomized*

3.1 Are the participants representative of the target population?

3.2 Are measurements appropriate regarding both the outcome and intervention (or exposure)?

3.3 Are there complete outcome data?

3.4 Are the confounders accounted for in the design and analysis?

3.5 During the study period, is the intervention administered (or exposure occurred) as intended?

5.5 Do the different components of the study adhere to the quality criteria of each tradition of the methods involved?

**Criteria from the Laboratory Bias Assessment:**

1. Adequate controls

2. Methods clearly described

3. Appropriate detection method

4. Studies performed at least in duplicate

5. Statistical analysis of data
