# Supplementary material for: Efficacy and effectiveness of hand hygiene-related practices used in community settings for removal of organisms from hands: a systematic review
Source: BMJ Glob Health. 2025 Sep 16;10(Suppl 7):e018925. doi: 10.1136/bmjgh-2025-018925 (PMC12443168; doi:10.1136/bmjgh-2025-018925)

Figure S8: Funnel plots for some of the meta-analyses reported to screen for possible publication bias. All meta-analyses reflected in these plots allowed any answer for hand rubbing, only included laboratory studies, and included all studies for the broad pathogen category (e.g. bacteria), instead of focusing on pathogen subcategories (e.g. gram-positive bacteria). The shape and color of points differ by study evaluated. The plots show the estimated effect size and standard error for each data point evaluated for a) handwashing with soap and water against bacteria, b) handwashing with soap and water against viruses, c) handwashing with water only against bacteria, d) handwashing with water only against viruses, e) alcohol-based hand rub against bacteria, f) alcohol-based hand rub against viruses, g) non-alcohol-based antiseptics against bacteria, h) non-alcohol-based antiseptics against viruses, i) soap alternatives against bacteria, and j) antiseptic/antimicrobial towels against bacteria.
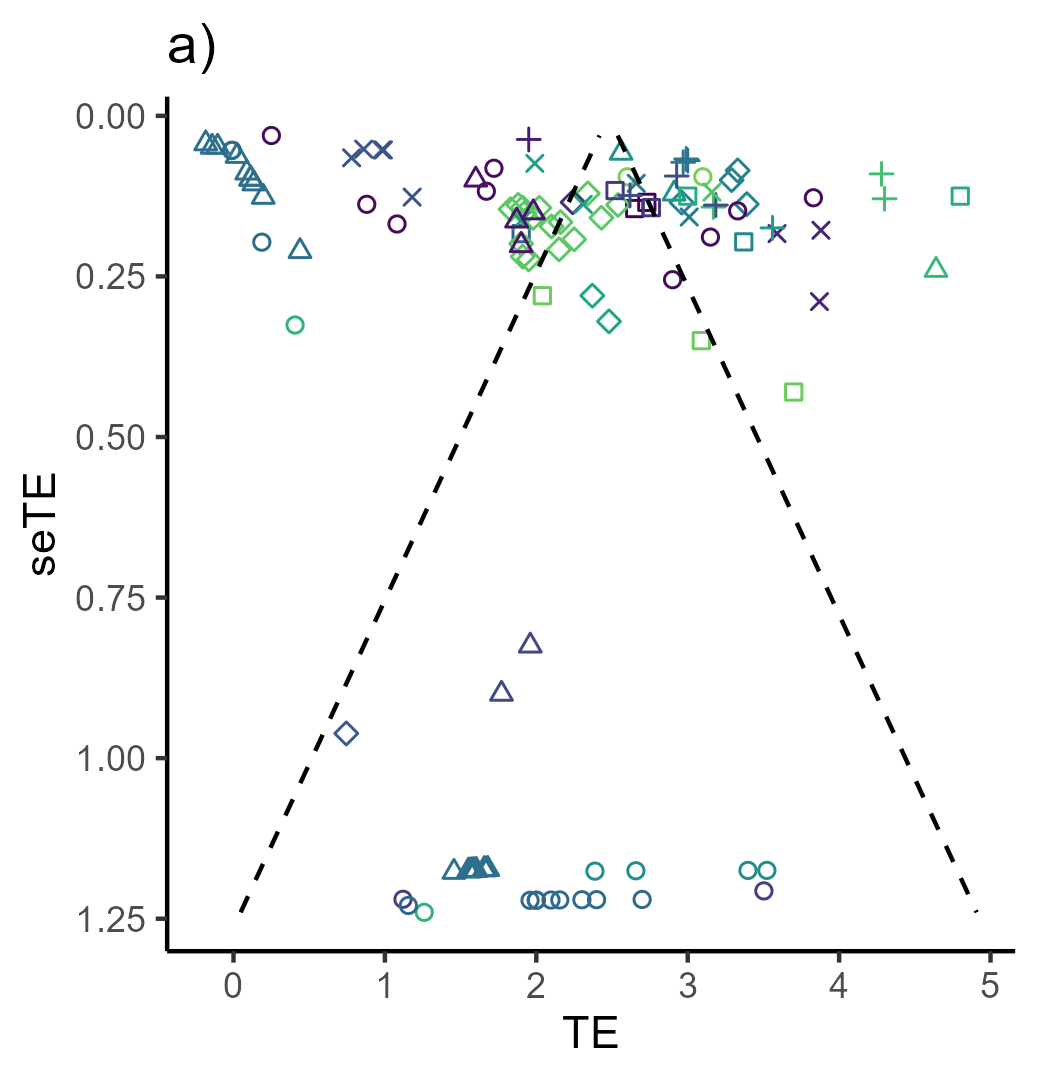

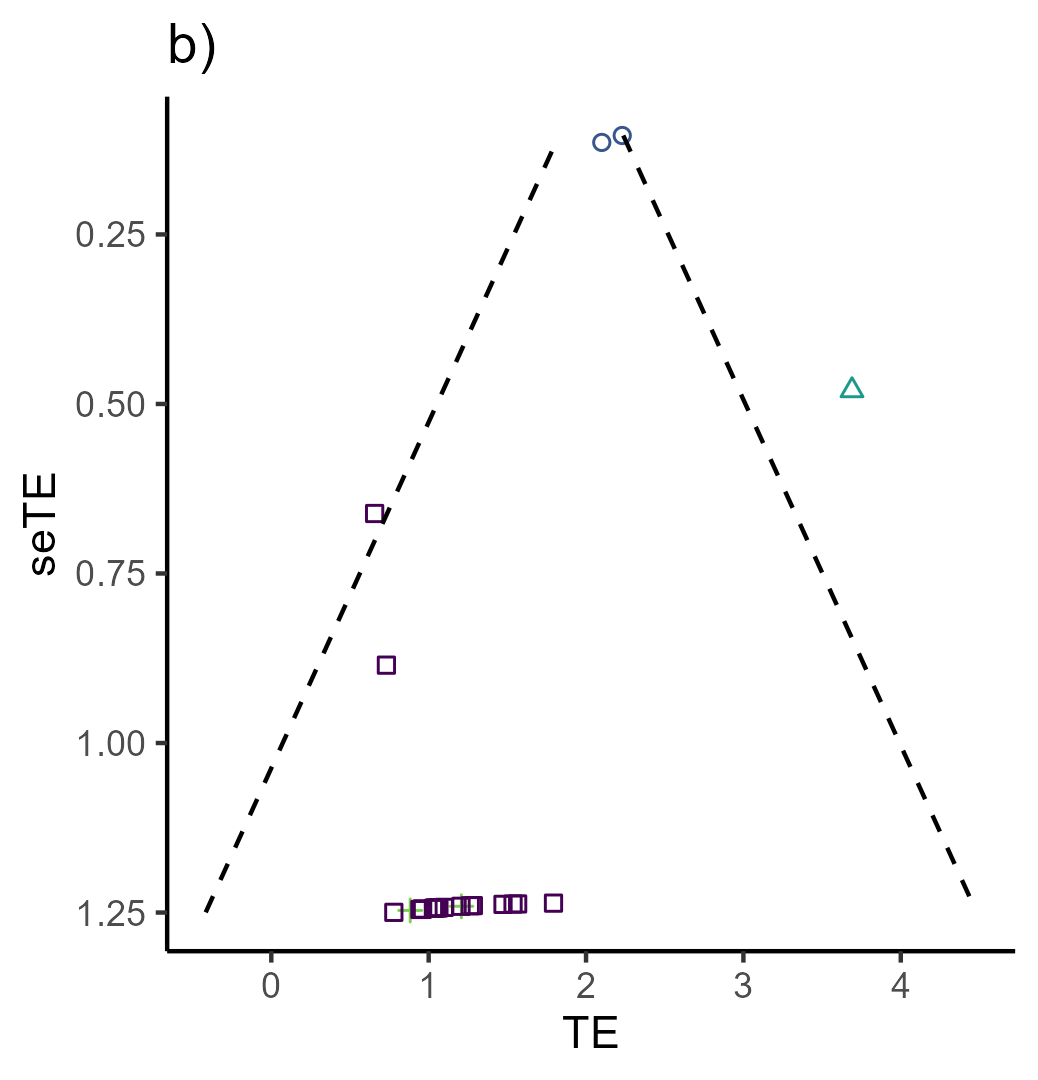

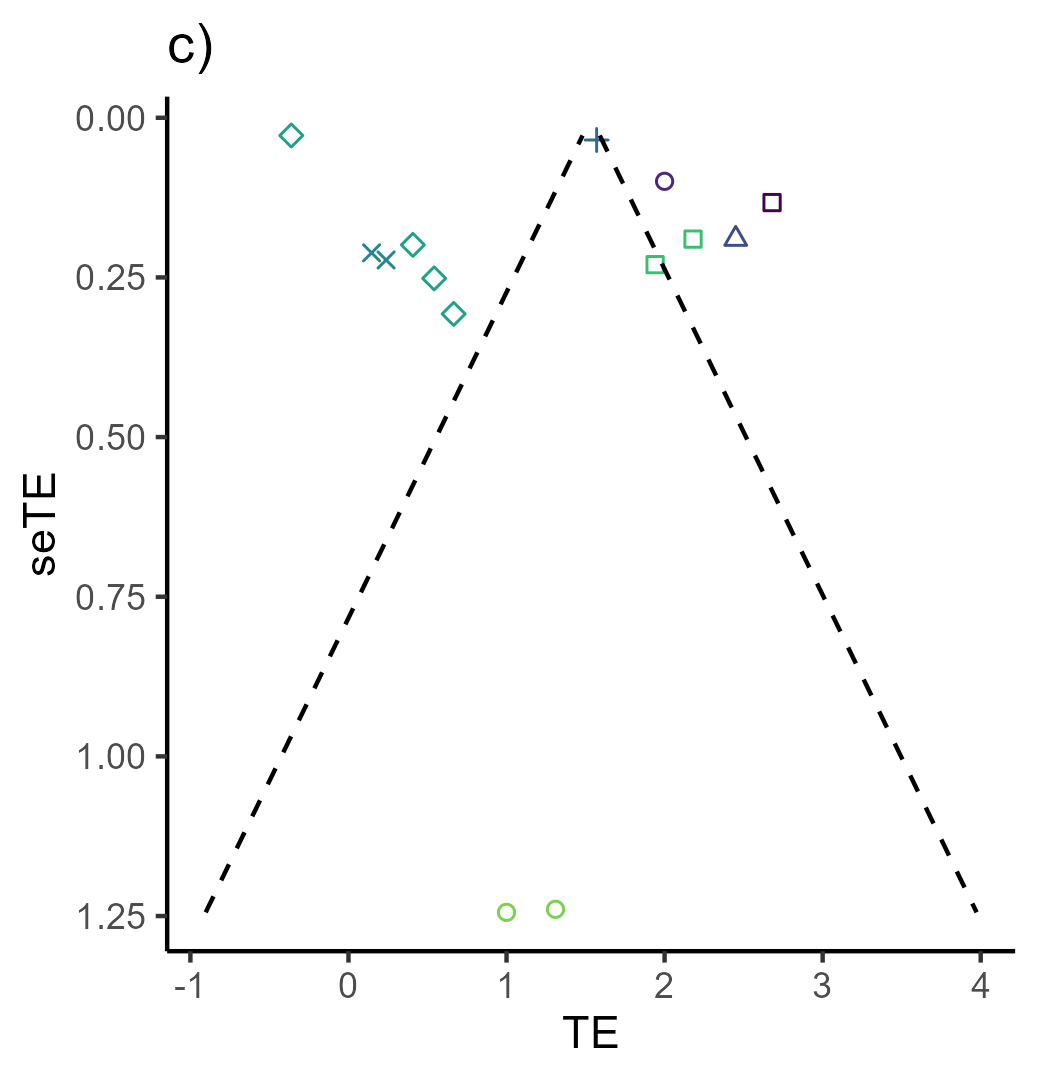

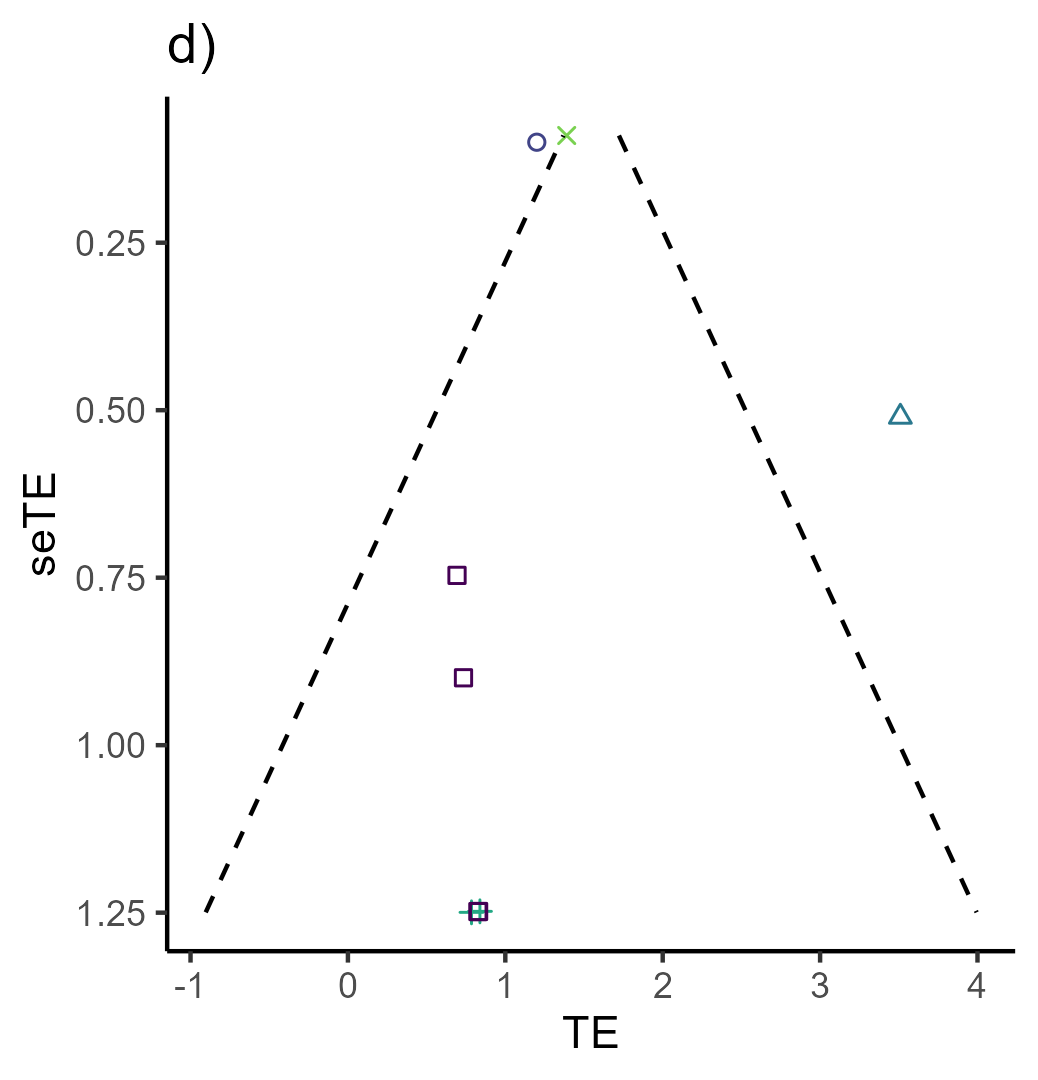

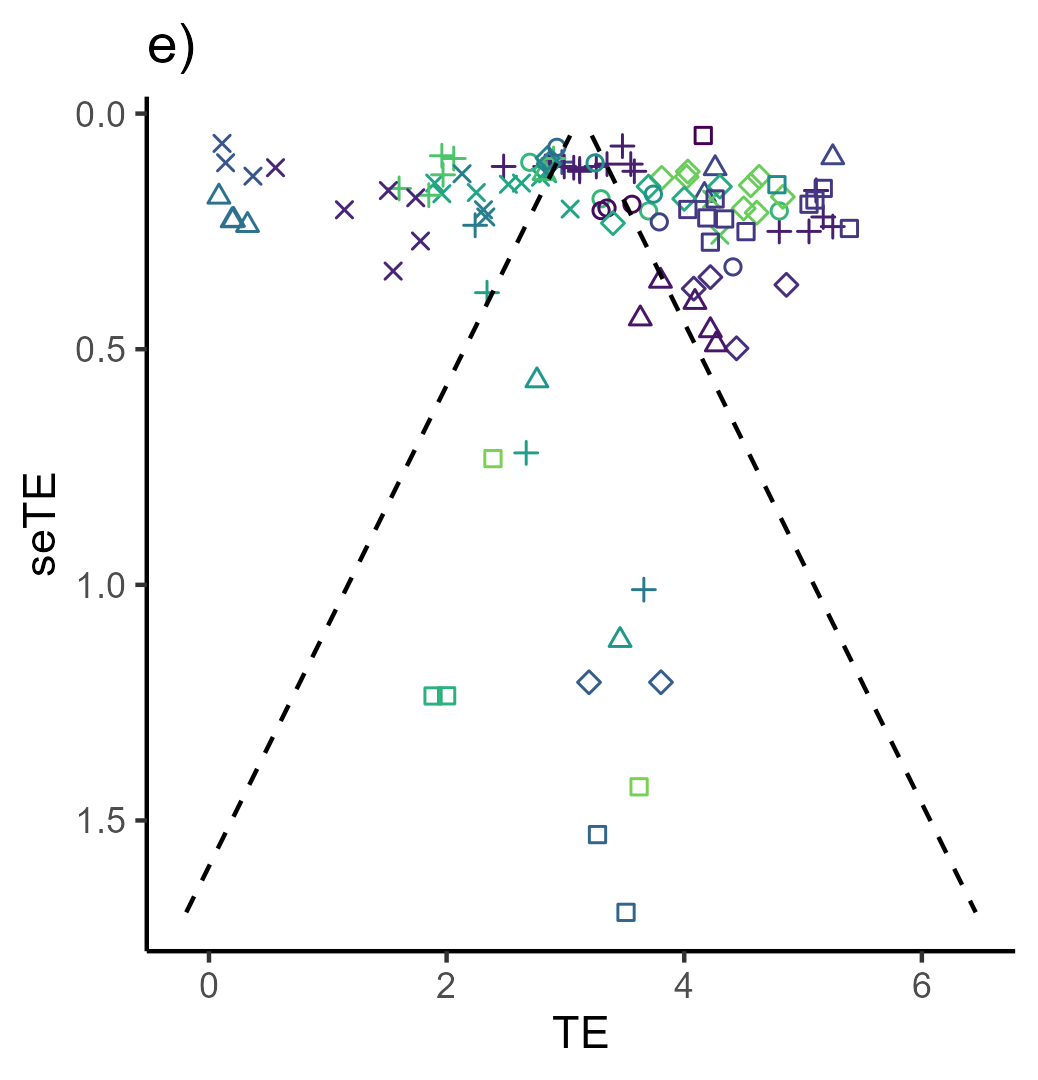

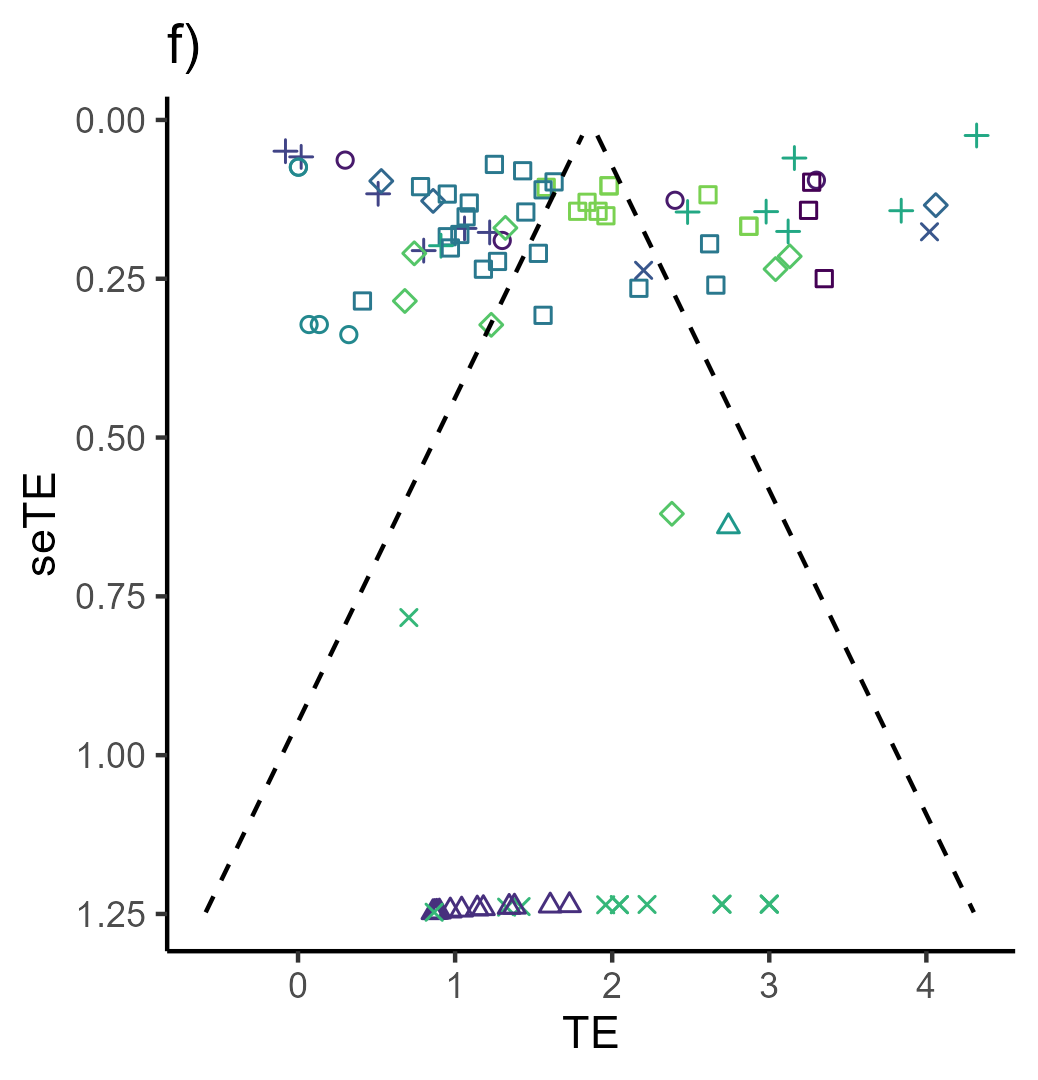

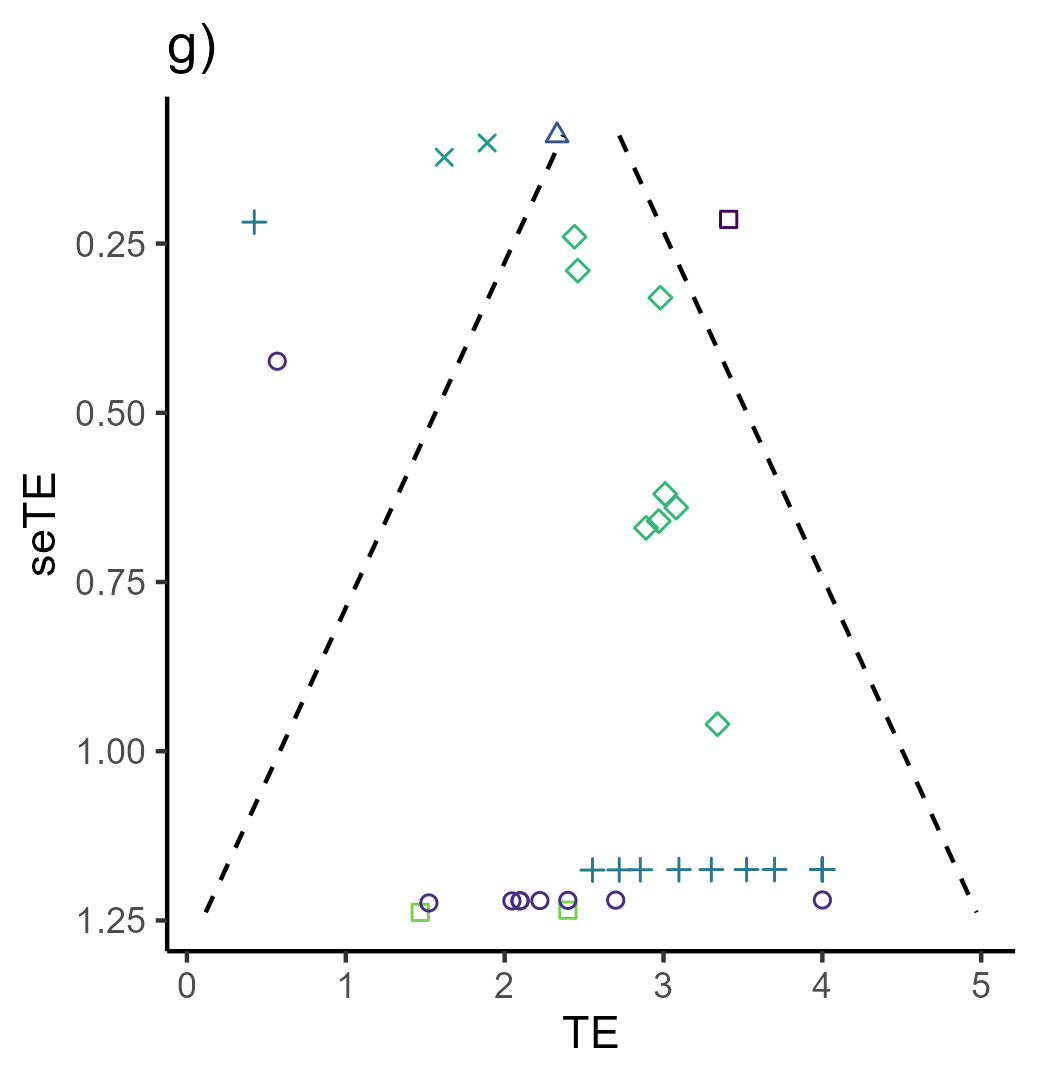

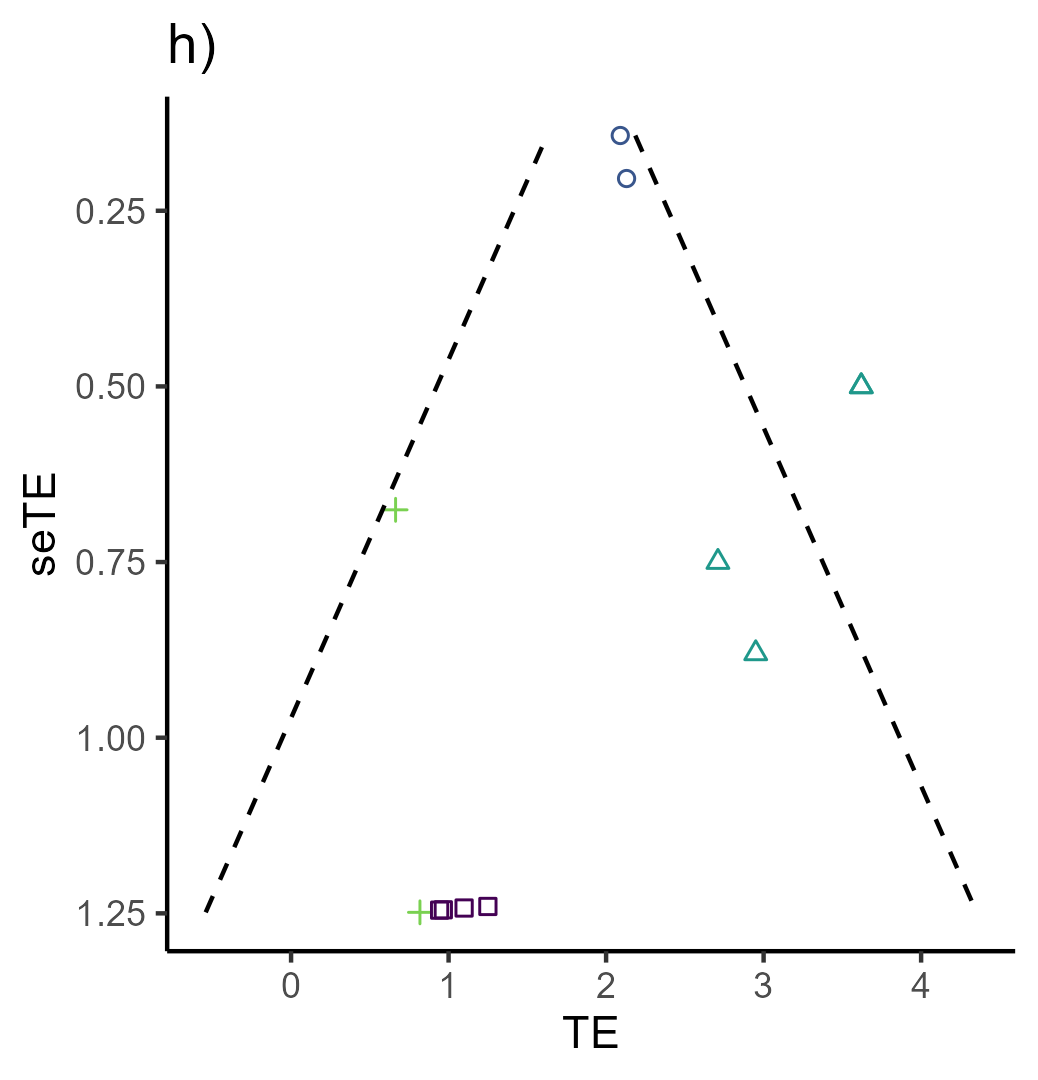

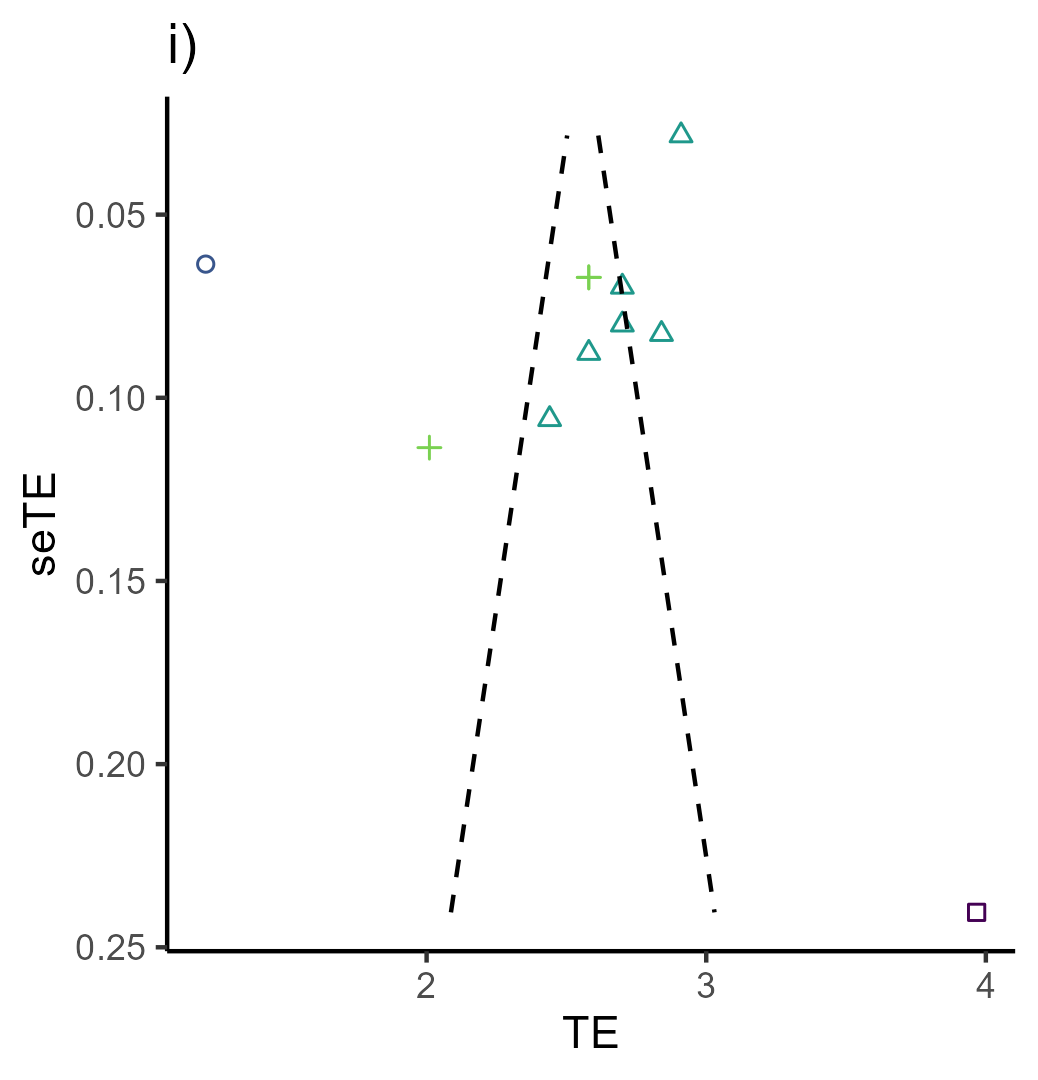

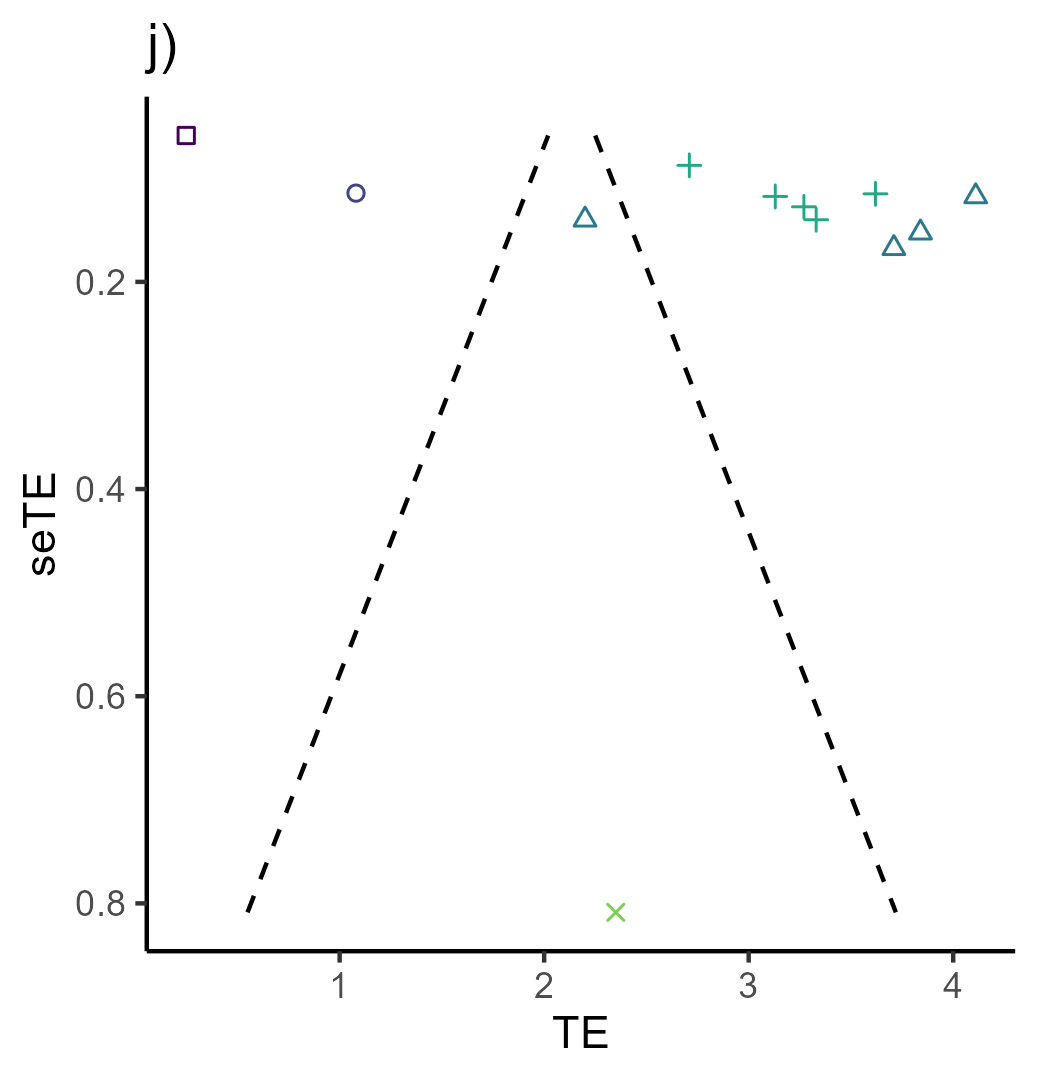

Supplement: online supplemental file 8 [file bmjgh-10-Suppl_7-s008.docx]
