# Supplementary material for: Efficacy and effectiveness of hand hygiene-related practices used in community settings for removal of organisms from hands: a systematic review
Source: BMJ Glob Health. 2025 Sep 16;10(Suppl 7):e018925. doi: 10.1136/bmjgh-2025-018925 (PMC12443168; doi:10.1136/bmjgh-2025-018925)
Supplement: online supplemental file 9 [file bmjgh-10-Suppl_7-s009.docx]

**Table S9a. Meta-analysis of studies reporting results on handwashing with soap and water. This table only shows comparisons where data were available, including those where data was insufficient for meta-analysis.**

| **Study Type** | **Hand rubbing** | **Handwashing materials** | **Type of organism** | **Summary log reduction (CI)** | **I2** | **Total number of studies** | **Total number of estimates** | **Total number of subjects** | **Mean MMAT Score** | **Mean Laboratory Score** |
| --- | --- | --- | --- | --- | --- | --- | --- | --- | --- | --- |
| Laboratory | Any Response | All soap | Bacteria | 2.48 (2.12 to 2.84) | 74 | 32 | 119 | 1388 | 4.1 | 4.8 |
|  |  |  | Virus | 2.03 (1.45 to 2.62) | 86 | 4 | 25 | 126 | 4.5 | 5 |
|  |  | Water only | Bacteria | 1.54 (0.83 to 2.24) | 86 | 8 | 14 | 150 | 4.2 | 5 |
|  |  |  | Virus | 1.55 (0.74 to 2.35) | 94 | 5 | 9 | 58 | 4.4 | 5 |
|  | Yes | All soap | Bacteria | 2.36 (1.98 to 2.74) | 65 | 25 | 90 | 894 | 4 | 4.8 |
|  |  |  | *Gram positive* | 1.36 (0.43 to 2.29) | 98 | 7 | 27 | 214 | 4 | 4.4 |
|  |  |  | *Gram negative* | 2.6 (2.33 to 2.86) | 25 | 21 | 63 | 680 | 4 | 4.9 |
|  |  | Plain soap | Bacteria | 2.34 (1.9 to 2.79) | 90 | 20 | 43 | 530 | 4 | 4.9 |
|  |  | Antimicrobial soap | Bacteria | 2.15 (1.4 to 2.89) | 95 | 13 | 41 | 340 | 4.1 | 4.8 |
|  |  | Water only | Bacteria | 1.4 (0.52 to 2.28) | 82 | 6 | 10 | 114 | 4 | 5 |
|  |  | All soap | Virus | 2.3 (1.48 to 3.12) | 92 | 3 | 13 | 90 | 4.3 | 5 |
|  |  |  | *Non-enveloped* | 1.71 (1.19 to 2.23) | 80 | 2 | 12 | 78 | 4.5 | 5 |
|  |  |  | *Enveloped* |  |  | 1 | 1 | 12 |  |  |
|  |  | Plain soap | Virus | 2.42 (0.78 to 4.05) | 96 | 3 | 5 | 66 | 4.3 | 5 |
|  |  | Antimicrobial soap | Virus |  |  | 1 | 8 | 24 |  |  |
|  |  | Water only | Virus |  |  | 3 | 4 | 42 |  |  |
|  | No | All soap | Bacteria |  |  | 1 | 2 | 6 |  |  |
|  |  |  | *Gram negative* |  |  | 1 | 2 | 6 |  |  |
|  |  | Plain soap | Bacteria |  |  | 1 | 2 | 6 |  |  |
|  |  | Water only | Bacteria |  |  | 2 | 3 | 33 |  |  |
|  |  | All soap | Virus |  |  | 1 | 2 | 6 |  |  |
|  |  |  | *Non-enveloped* |  |  | 1 | 2 | 6 |  |  |
|  |  | Plain soap | Virus |  |  | 1 | 2 | 6 |  |  |
|  |  | Water only | Virus |  |  | 2 | 3 | 10 |  |  |
| Field | Any Response | All soap | Bacteria | 0.44 (-0.08 to 0.96) | 83 | 2 | 25 | 1412 | 4 | 4.5 |
|  | Yes | All soap | Bacteria | 0.48 (-0.02 to 0.97) | 85 | 2 | 24 | 1408 | 4 | 4.5 |
|  |  |  | *Gram positive* | 0.5 (-0.08 to 1.08) | 93 | 2 | 8 | 688 | 4 | 4.5 |
|  |  |  | *Gram negative* |  |  | 1 | 4 | 16 |  |  |
|  |  | Plain soap | Bacteria |  |  | 1 | 4 | 672 |  |  |
|  |  | Antimicrobial soap | Bacteria |  |  | 1 | 8 | 32 |  |  |
|  |  | Water only | Bacteria |  |  | 1 | 2 | 168 |  |  |

**Table S9b. Meta-analysis of studies reporting results on the duration of handwashing with soap and water for removal of bacteria and viruses with soap and water. This table only shows comparisons where data were available, including those where data was insufficient for meta-analysis.**

| **Study Type** | **Handwashing Duration** | **Handwashing materials** | **Type of organism** | **Summary log reduction (CI)** | **I2** | **Total number of studies** | **Total number of estimates** | **Total number of subjects** | **Mean MMAT Score** | **Mean Laboratory Score** |
| --- | --- | --- | --- | --- | --- | --- | --- | --- | --- | --- |
| Laboratory | 10-20 seconds | Any soap | Bacteria | 2.19 (1.5 to 2.87) | 46 | 6 | 21 | 184 | 4.5 | 4.8 |
|  |  | *Plain soap* |  | 2.45 (1.75 to 3.15) | 97 | 5 | 7 | 118 | 4.4 | 5 |
|  |  | *Antibacterial soap* |  | 1.89 (0.97 to 2.81) | 0 | 3 | 8 | 42 | 4.3 | 4.7 |
|  |  | Water only |  | 0.93 (-0.9 to 2.75) | 85 | 3 | 6 | 63 | 4 | 5 |
|  |  | Any soap | Virus |  |  | 1 | 2 | 48 |  |  |
|  |  | *Plain soap* |  |  |  | 1 | 2 | 48 |  |  |
|  | 20-30 seconds | Any soap | Bacteria |  |  | 1 | 4 | 20 |  |  |
|  |  | *Plain soap* |  |  |  | 1 | 1 | 5 |  |  |
|  |  | *Antibacterial soap* |  |  |  | 1 | 3 | 15 |  |  |
|  |  | Water only |  |  |  | 2 | 3 | 41 |  |  |
|  |  | Water only |  |  |  | 1 | 1 | 12 |  |  |
|  | 30 seconds + | Any soap | Bacteria | 2.49 (2.01 to 2.96) | 90 | 20 | 60 | 637 | 4 | 4.8 |
|  |  | *Plain soap* |  | 2.36 (1.78 to 2.93) | 99 | 16 | 32 | 376 | 3.9 | 4.9 |
|  |  | *Antibacterial soap* |  | 2.34 (1.47 to 3.22) | 98 | 10 | 28 | 261 | 4 | 4.8 |
|  |  | Any soap | Virus |  |  | 1 | 1 | 12 |  |  |
|  |  | *Plain soap* |  |  |  | 1 | 1 | 12 |  |  |
| Field | 10-20 seconds | Any soap | Bacteria |  |  | 1 | 2 | 336 |  |  |
|  |  | *Plain soap* |  |  |  | 1 | 2 | 336 |  |  |
|  |  | Water only |  |  |  | 1 | 2 | 168 |  |  |
|  | 30 seconds + | Any soap | Bacteria |  |  | 1 | 2 | 336 |  |  |
|  |  | *Plain soap* |  |  |  | 1 | 2 | 336 |  |  |

**Table S9c. Meta-analysis of studies reporting results on handwashing with soap alternatives. This table only shows comparisons where data were available, including those where data was insufficient for meta-analysis.**

| **Study Type** | **Handwashing materials** | **Hand rubbing** | **Type of organism** | **Summary log reduction (CI)** | **I2** | **Total number of studies** | **Total number of estimates** | **Total number of subjects** | **Mean MMAT Score** | **Mean Laboratory Score** |
| --- | --- | --- | --- | --- | --- | --- | --- | --- | --- | --- |
| Laboratory | Alcohol-based hand rub | All Responses | Bacteria | 3.13 (2.7 to 3.56) | 79 | 31 | 117 | 1566 | 4 | 4.8 |
|  |  | Yes | Bacteria | 3.12 (2.59 to 3.65) | 82 | 24 | 99 | 1300 | 4 | 4.9 |
|  |  |  | *Gram positive* | 2.24 (-0.36 to 4.84) | 99 | 3 | 7 | 58 | 4 | 5 |
|  |  |  | *Gram negative* | 3.24 (2.71 to 3.76) | 78 | 21 | 92 | 1242 | 4 | 4.9 |
|  | Non-alcohol-based antiseptics | All Responses | Bacteria | 2.54 (2.13 to 2.96) | 18 | 7 | 40 | 388 | 4.1 | 4.6 |
|  |  | Yes | Bacteria | 2.49 (2.02 to 2.97) | 21 | 5 | 37 | 367 | 4 | 4.6 |
|  |  |  | *Gram positive* | 2.37 (0.97 to 3.77) | - | 2 | 8 | 52 | 4 | 4 |
|  |  |  | *Gram negative* | 2.5 (2.11 to 2.89) | 77 | 5 | 29 | 315 | 4 | 4.6 |
|  | Soap alternatives | All Responses | Bacteria | 2.56 (1.31 to 3.81) | 96 | 4 | 11 | 160 | 4.8 | 5 |
|  |  | Yes | Bacteria | 2.55 (1.21 to 3.89) | 91 | 4 | 7 | 100 | 4.8 | 5 |
|  |  |  | *Gram positive* |  |  | 1 | 1 | 12 |  |  |
|  |  |  | *Gram negative* | 2.96 (1.78 to 4.15) | 84 | 3 | 6 | 88 | 5 | 5 |
|  | Antiseptic/antimicrobial towels | All Responses | Bacteria | 2.13 (0.72 to 3.55) | 82 | 5 | 12 | 175 | 4 | 4.8 |
|  |  | Yes | Bacteria | 2.2 (0.67 to 3.73) | 76 | 5 | 9 | 127 | 4 | 4.8 |
|  |  |  | *Gram positive* |  |  | 1 | 1 | 6 |  |  |
|  |  |  | *Gram negative* | 2.47 (0.66 to 4.27) | 77 | 4 | 8 | 121 | 4 | 4.8 |
|  | Alcohol based hand rub | No | Bacteria |  |  | 2 | 3 | 21 |  |  |
|  |  |  | *Gram negative* |  |  | 2 | 3 | 21 |  |  |
|  | Non-alcohol-based antiseptics | No | Bacteria |  |  | 1 | 2 | 6 |  |  |
|  |  |  | *Gram negative* |  |  | 1 | 2 | 6 |  |  |
|  | Alcohol-based hand rub | All Responses | Virus | 1.86 (1.37 to 2.35) | 56 | 13 | 93 | 792 | 4 | 4.9 |
|  |  | Yes | Virus | 1.59 (0.72 to 2.45) | 78 | 7 | 33 | 315 | 4.3 | 4.9 |
|  |  |  | *Non-enveloped* | 1.1 (0.3 to 1.89) | 66 | 5 | 29 | 273 | 4.4 | 4.8 |
|  |  |  | *Enveloped* |  |  | 2 | 4 | 42 |  |  |
|  | Non-alcohol-based antiseptics | All Responses | Virus | 1.91 (1.05 to 2.76) | 87 | 4 | 12 | 78 | 4.8 | 5 |
|  |  | Yes | Virus | 2.36 (0.23 to 4.49) | 81 | 2 | 6 | 54 | 4.5 | 5 |
|  |  |  | *Non-enveloped* |  |  | 1 | 2 | 6 |  |  |
|  |  |  | *Enveloped* |  |  | 1 | 4 | 48 |  |  |
|  | Antiseptic/antimicrobial towels | All Responses | Virus |  |  | 1 | 1 | 3 |  |  |
|  | Alcohol-based hand rub | No | Virus | 2.05 (1.44 to 2.66) | 45 | 6 | 51 | 399 | 4 | 5 |
|  |  |  | *Non-enveloped* | 2.05 (1.44 to 2.66) | 45 | 6 | 51 | 399 | 4 | 5 |
|  | Non-alcohol-based antiseptics | No | Virus |  |  | 2 | 4 | 18 |  |  |
|  |  |  | *Non-enveloped* |  |  | 2 | 4 | 18 |  |  |
| Field | Non-alcohol-based antiseptics | Yes | Bacteria |  |  | 1 | 12 | 48 |  |  |
|  |  |  | *Gram positive* |  |  | 1 | 3 | 12 |  |  |
|  |  |  | *Gram negative* |  |  | 1 | 3 | 12 |  |  |

**Table S9d. Meta-analysis of studies reporting results on hand drying. This table only shows comparisons where data were available, including those where data was insufficient for meta-analysis.**

| **Study Type** | **Hand rubbing** | **Type of organism** | **Drying materials** | **Summary log reduction (CI)** | **I2** | **Total number of studies** | **Total number of estimates** | **Total number of subjects** | **Mean MMAT Score** | **Mean Laboratory Score** |
| --- | --- | --- | --- | --- | --- | --- | --- | --- | --- | --- |
| Laboratory | Yes | Bacteria | Any drying approach |  |  | 1 | 5 | 150 |  |  |
|  |  |  | Paper towels |  |  | 1 | 2 | 60 |  |  |
|  |  |  | Hot air dryer |  |  | 1 | 2 | 60 |  |  |
|  |  |  | Jet air dryer |  |  | 1 | 1 | 30 |  |  |

**Table S9e. Meta-analysis of studies reporting results on water quality. This table only shows comparisons where data were available, including those where data was insufficient for meta-analysis.**

| **Study Type** | **Type of Organism** | **Summary log reduction (CI)** | **I2** | **Total number of studies** | **Total number of estimates** | **Total number of subjects** | **Mean MMAT Score** | **Mean Laboratory Score** |
| --- | --- | --- | --- | --- | --- | --- | --- | --- |
| Laboratory | Bacteria |  |  | 1 | 2 | 32 |  |  |
|  | *Gram-negative* |  |  | 1 | 2 | 32 |  |  |
