# Supplementary material for: Efficacy and effectiveness of hand hygiene-related practices used in community settings for removal of organisms from hands: a systematic review
Source: BMJ Glob Health. 2025 Sep 16;10(Suppl 7):e018925. doi: 10.1136/bmjgh-2025-018925 (PMC12443168; doi:10.1136/bmjgh-2025-018925)
Supplement: online supplemental file 10 [file bmjgh-10-Suppl_7-s010.docx]

**S10.** Summary of the included estimates.

| **Hand Hygiene Materials** | **Pathogen type** | **Study Design (Laboratory or field efficacy study)** | **Total studies available** | **Total number of estimates** | **Number of estimates with log reduction available** | **Number of estimates with uncertainty available** | **Number of estimates with duration available** | **Number of estimates with microbial water quality** |
| --- | --- | --- | --- | --- | --- | --- | --- | --- |
| Handwashing with soap and water | **(total)** |  | **110** | **627** | **519** | **242** | **473** | **2** |
|  | Gram-negative bacteria | Field | 7 | 23 | 20 | 4 | 10 | 0 |
|  |  | Laboratory | 53 | 245 | 203 | 97 | 198 | 2 |
|  | Gram-positive bacteria | Field | 4 | 12 | 9 | 9 | 4 | 0 |
|  |  | Laboratory | 23 | 102 | 68 | 27 | 98 | 0 |
|  | Bacteria (unspecified) | Field | 4 | 30 | 30 | 12 | 18 | 0 |
|  |  | Laboratory | 3 | 16 | 16 | 0 | 15 | 0 |
|  | Fungus | Laboratory | 1 | 1 | 1 | 1 | 1 | 0 |
|  | Enveloped virus | Laboratory | 3 | 5 | 2 | 1 | 4 | 0 |
|  | Non-enveloped virus | Laboratory | 15 | 55 | 45 | 24 | 21 | 0 |
|  | Unspecified pathogens | Field | 6 | 17 | 14 | 5 | 11 | 0 |
|  |  | Laboratory | 22 | 121 | 111 | 62 | 93 | 0 |
| Handwashing with water only | **(total)** |  | **32** | **90** | **56** | **25** | **58** | **6** |
|  | Gram-negative bacteria | Field | 2 | 3 | 3 | 0 | 3 | 2 |
|  |  | Laboratory | 12 | 31 | 14 | 11 | 16 | 0 |
|  | Gram-positive bacteria | Field | 1 | 1 | 1 | 1 | 1 | 0 |
|  |  | Laboratory | 7 | 12 | 6 | 3 | 9 | 0 |
|  | Bacteria (unspecified) | Field | 2 | 5 | 5 | 1 | 5 | 4 |
|  | Enveloped virus | Laboratory | 3 | 3 | 2 | 1 | 3 | 0 |
|  | Non-enveloped virus | Laboratory | 12 | 22 | 17 | 8 | 11 | 0 |
|  | Unspecified pathogens | Laboratory | 4 | 13 | 8 | 0 | 10 | 0 |
| **Alcohol-based hand rub** | **(total)** |  | **111** | **738** | **574** | **254** |  |  |
|  | Gram-negative bacteria | Field | 1 | 2 | 0 | 0 |  |  |
|  |  | Laboratory | 60 | 380 | 330 | 116 |  |  |
|  | Gram-positive bacteria | Field | 2 | 10 | 0 | 0 |  |  |
|  |  | Laboratory | 16 | 90 | 37 | 7 |  |  |
|  | Bacteria (unspecified) | Field | 1 | 4 | 0 | 0 |  |  |
|  |  | Laboratory | 1 | 1 | 1 | 0 |  |  |
|  | Fungus | Field | 1 | 4 | 4 | 4 |  |  |
|  |  | Laboratory | 1 | 2 | 0 | 0 |  |  |
|  | Enveloped virus | Laboratory | 6 | 15 | 4 | 4 |  |  |
|  | Non-enveloped virus | Laboratory | 23 | 150 | 125 | 89 |  |  |
|  | Unspecified pathogens | Field | 3 | 3 | 1 | 0 |  |  |
|  |  | Laboratory | 16 | 77 | 72 | 34 |  |  |
| **Non-alcoholic antiseptics** | **(total)** |  | **25** | **132** | **112** | **73** |  |  |
|  | Gram-negative bacteria | Field | 1 | 3 | 3 | 3 |  |  |
|  |  | Laboratory | 13 | 43 | 42 | 32 |  |  |
|  | Gram-positive bacteria | Field | 1 | 3 | 3 | 3 |  |  |
|  |  | Laboratory | 5 | 16 | 9 | 9 |  |  |
|  | Bacteria (unspecified) | Field | 1 | 6 | 6 | 6 |  |  |
|  |  | Laboratory | 2 | 2 | 2 | 0 |  |  |
|  | Enveloped virus | Laboratory | 2 | 5 | 4 | 4 |  |  |
|  | Non-enveloped virus | Laboratory | 7 | 22 | 19 | 8 |  |  |
|  | Unspecified pathogens | Laboratory | 5 | 32 | 24 | 8 |  |  |
| **Handwashing with soap alternatives** | **(total)** |  | **15** | **42** | **25** | **12** |  |  |
|  | Gram-negative bacteria | Field | 1 | 1 | 1 | 0 |  |  |
|  |  | Laboratory | 9 | 28 | 13 | 10 |  |  |
|  | Gram-positive bacteria | Laboratory | 1 | 1 | 1 | 1 |  |  |
|  | Enveloped virus | Laboratory | 1 | 4 | 4 | 0 |  |  |
|  | Non-enveloped virus | Laboratory | 2 | 5 | 4 | 0 |  |  |
|  | Unspecified pathogens | Laboratory | 2 | 3 | 2 | 1 |  |  |
| **Handwashing with antimicrobial wipes** | **(total)** |  | **10** | **49** | **21** | **13** |  |  |
|  | Gram-negative bacteria | Laboratory | 6 | 18 | 12 | 11 |  |  |
|  | Gram-positive bacteria | Field | 1 | 7 | 0 | 0 |  |  |
|  |  | Laboratory | 3 | 5 | 2 | 1 |  |  |
|  | Bacteria (unspecified) | Field | 1 | 4 | 0 | 0 |  |  |
|  | Fungus | Field | 1 | 2 | 0 | 0 |  |  |
|  | Enveloped virus | Laboratory | 1 | 6 | 0 | 0 |  |  |
|  | Non-enveloped virus | Laboratory | 1 | 1 | 1 | 1 |  |  |
|  | Unspecified pathogens | Laboratory | 1 | 6 | 6 | 0 |  |  |
| **Hand drying** | **(total)** |  | **12** | **141** | **88** | **40** |  |  |
| Cloth towel | Gram-negative bacteria | Laboratory | 2 | 12 | 1 | 1 |  |  |
|  | Gram-positive bacteria | Laboratory | 1 | 1 | 1 | 0 |  |  |
|  | Non-enveloped virus | Laboratory | 1 | 3 | 0 | 0 |  |  |
|  | Unspecified pathogens | Laboratory | 1 | 1 | 1 | 0 |  |  |
| Evaporation | Gram-positive bacteria | Laboratory | 1 | 1 | 1 | 0 |  |  |
| Hot air dryer | Gram-negative bacteria | Laboratory | 3 | 13 | 5 | 2 |  |  |
|  | Gram-positive bacteria | Laboratory | 1 | 1 | 1 | 0 |  |  |
|  | Bacteria (unspecified) | Laboratory | 1 | 15 | 15 | 0 |  |  |
|  | Non-enveloped virus | Laboratory | 1 | 3 | 0 | 0 |  |  |
|  | Unspecified pathogens | Field | 1 | 1 | 0 | 0 |  |  |
|  |  | Laboratory | 3 | 20 | 18 | 14 |  |  |
| Jet air dryer | Gram-negative bacteria | Laboratory | 1 | 1 | 1 | 1 |  |  |
|  | Gram-positive bacteria | Field | 1 | 3 | 2 | 0 |  |  |
|  | Bacteria (unspecified) | Field | 1 | 6 | 2 | 0 |  |  |
|  | Unspecified pathogens | Field | 1 | 1 | 0 | 0 |  |  |
|  |  | Laboratory | 1 | 1 | 0 | 0 |  |  |
| Other | Gram-negative bacteria | Laboratory | 1 | 1 | 1 | 0 |  |  |
|  | Unspecified pathogens | Laboratory | 1 | 10 | 10 | 10 |  |  |
| Paper towels | Gram-negative bacteria | Laboratory | 4 | 14 | 3 | 3 |  |  |
|  | Gram-positive bacteria | Laboratory | 1 | 1 | 1 | 0 |  |  |
|  | Bacteria (unspecified) | Laboratory | 1 | 15 | 15 | 0 |  |  |
|  | Non-enveloped virus | Laboratory | 1 | 5 | 0 | 0 |  |  |
|  | Unspecified pathogens | Field | 1 | 1 | 0 | 0 |  |  |
|  |  | Laboratory | 3 | 11 | 10 | 9 |  |  |
| **Microbial Water Quality** | **(total)** |  | **2** | **10** | **10** | **4** |  |  |
|  | Gram-negative bacteria | Field | 1 | 2 | 2 | 0 |  |  |
|  | Gram-negative bacteria | Laboratory | 1 | 4 | 4 | 4 |  |  |
|  | Unspecified pathogens | Field | 1 | 4 | 4 | 0 |  |  |
