# Supplementary material for: Efficacy and effectiveness of hand hygiene-related practices used in community settings for removal of organisms from hands: a systematic review
Source: BMJ Glob Health. 2025 Sep 16;10(Suppl 7):e018925. doi: 10.1136/bmjgh-2025-018925 (PMC12443168; doi:10.1136/bmjgh-2025-018925)

Figure S11: Forest plots for some of the meta-analyses reported. All meta-analyses reflected in these plots allowed any answer for hand rubbing, only included laboratory studies, and included all studies for the broad pathogen category (e.g. bacteria), instead of focusing on pathogen subcategories (e.g. gram-positive bacteria). The bottom line labeled “RE Model” reports the summary log_10_ reduction value and its confidence interval, which is shown via the diamond in that row. The dotted line represents a log_10_ reduction value of 0 (no effect), and the dashed line shows the summary log_10_ reduction value. The plots reflect the estimated effect size of a) handwashing with soap and water against bacteria, b) handwashing with soap and water against viruses, c) handwashing with water only against bacteria, d) handwashing with water only against viruses, e) alcohol-based hand rub against bacteria, f) alcohol-based hand rub against viruses, g) non-alcohol-based antiseptics against bacteria, h) non-alcohol-based antiseptics against viruses, i) soap alternatives against bacteria, and j) antiseptic/antimicrobial towels against bacteria.


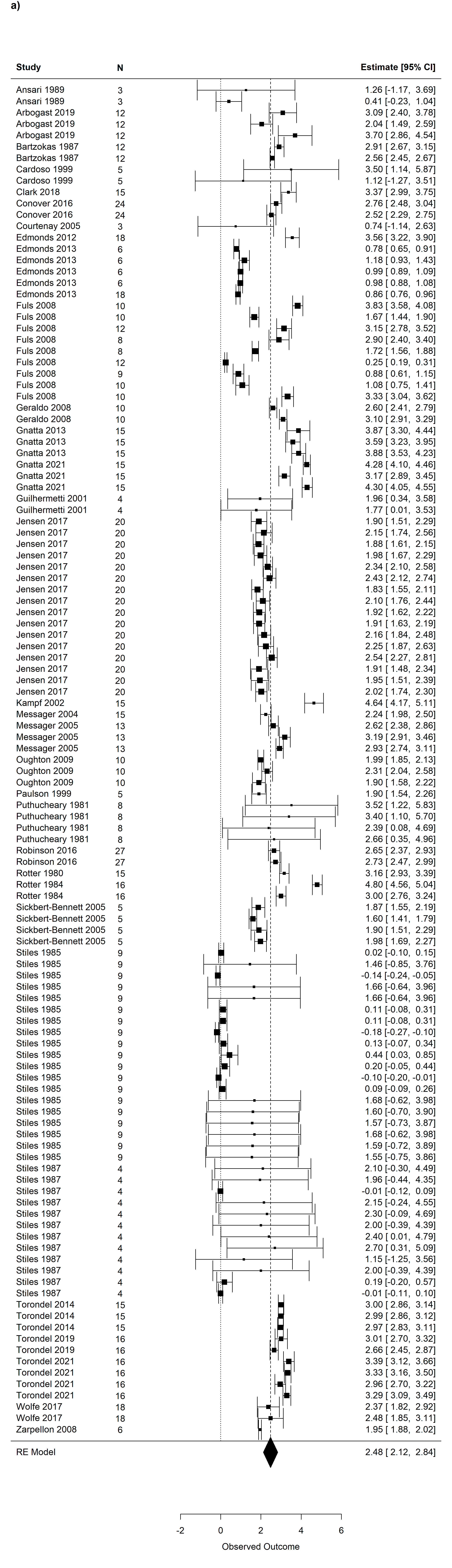


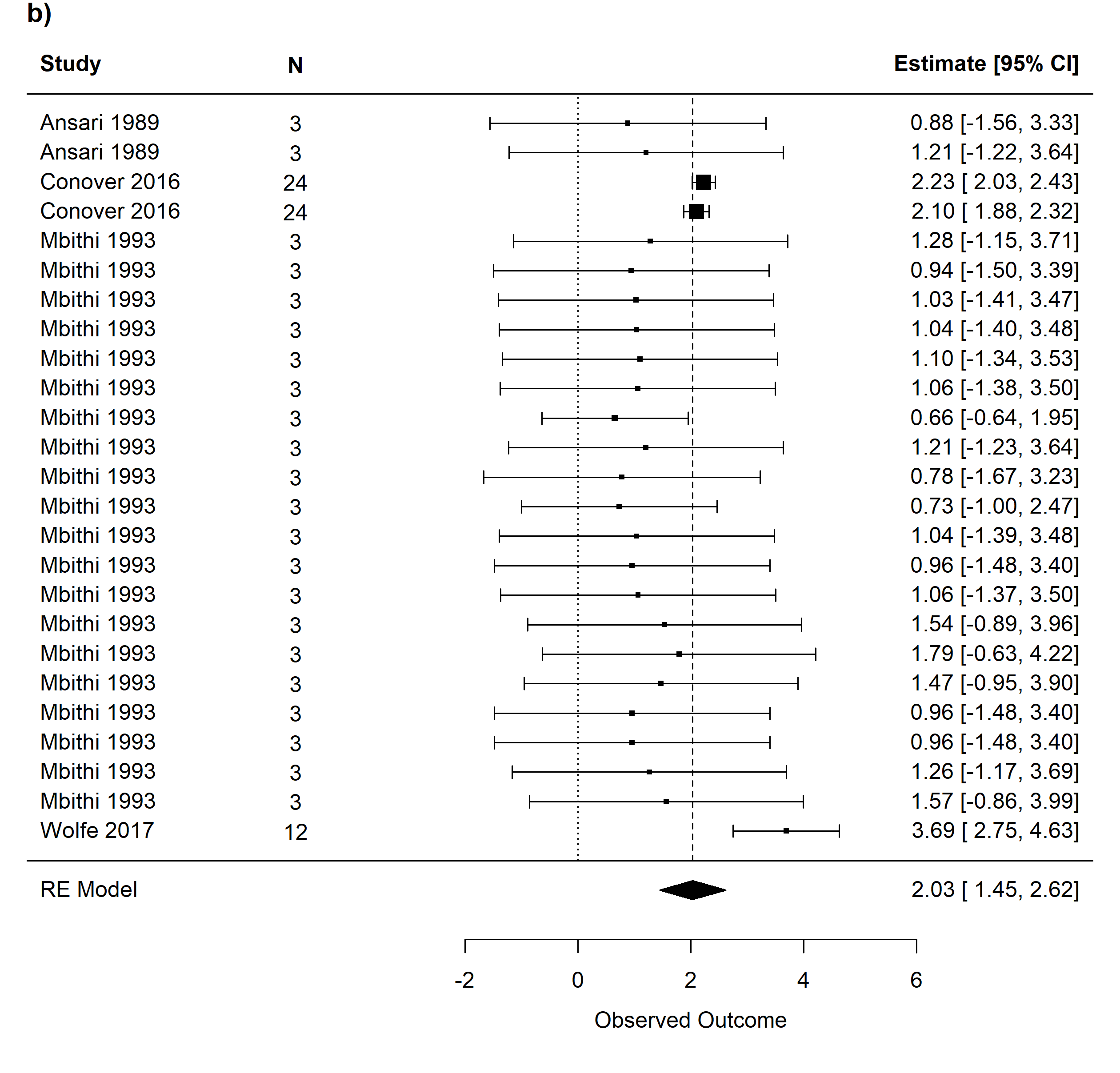


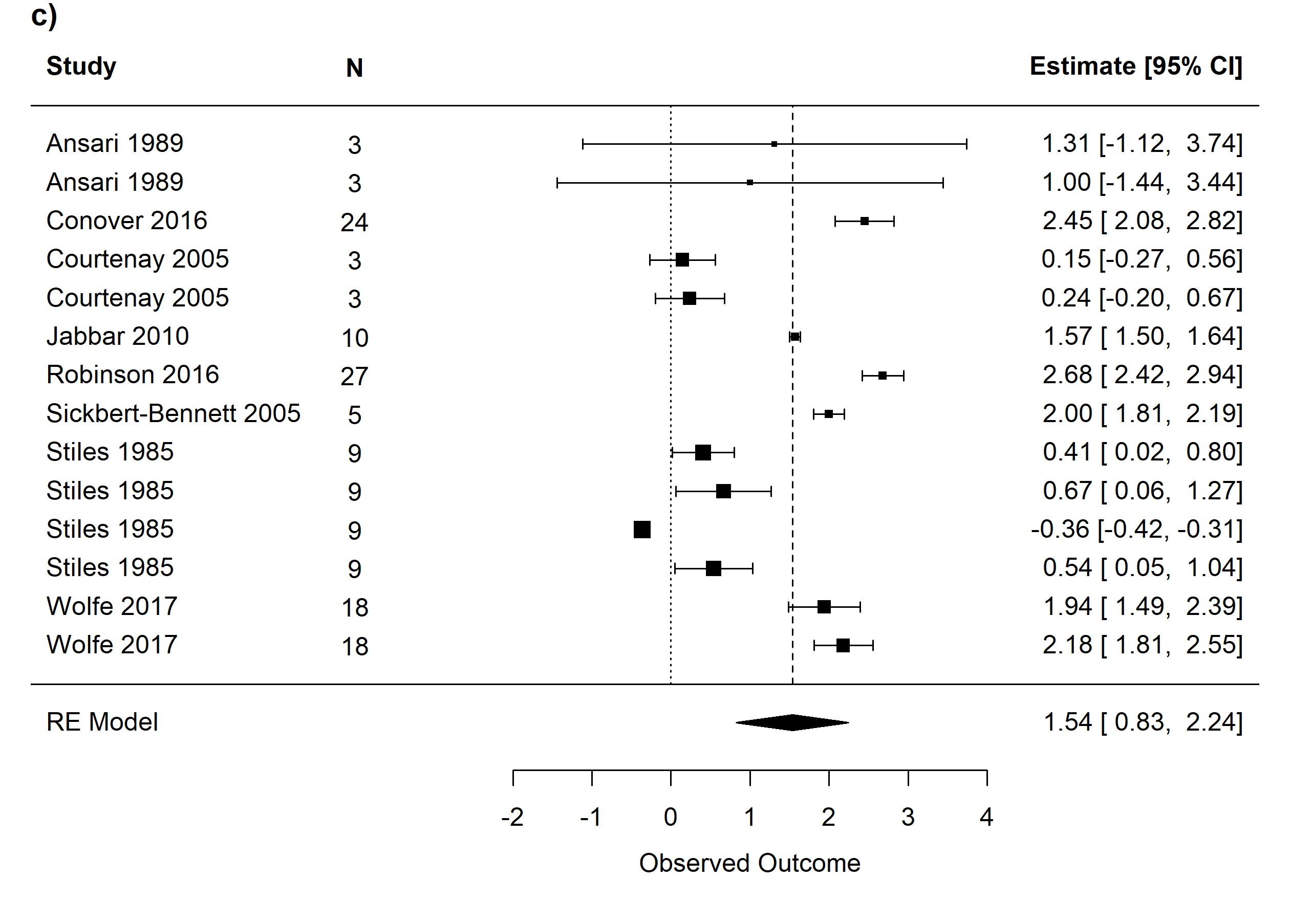


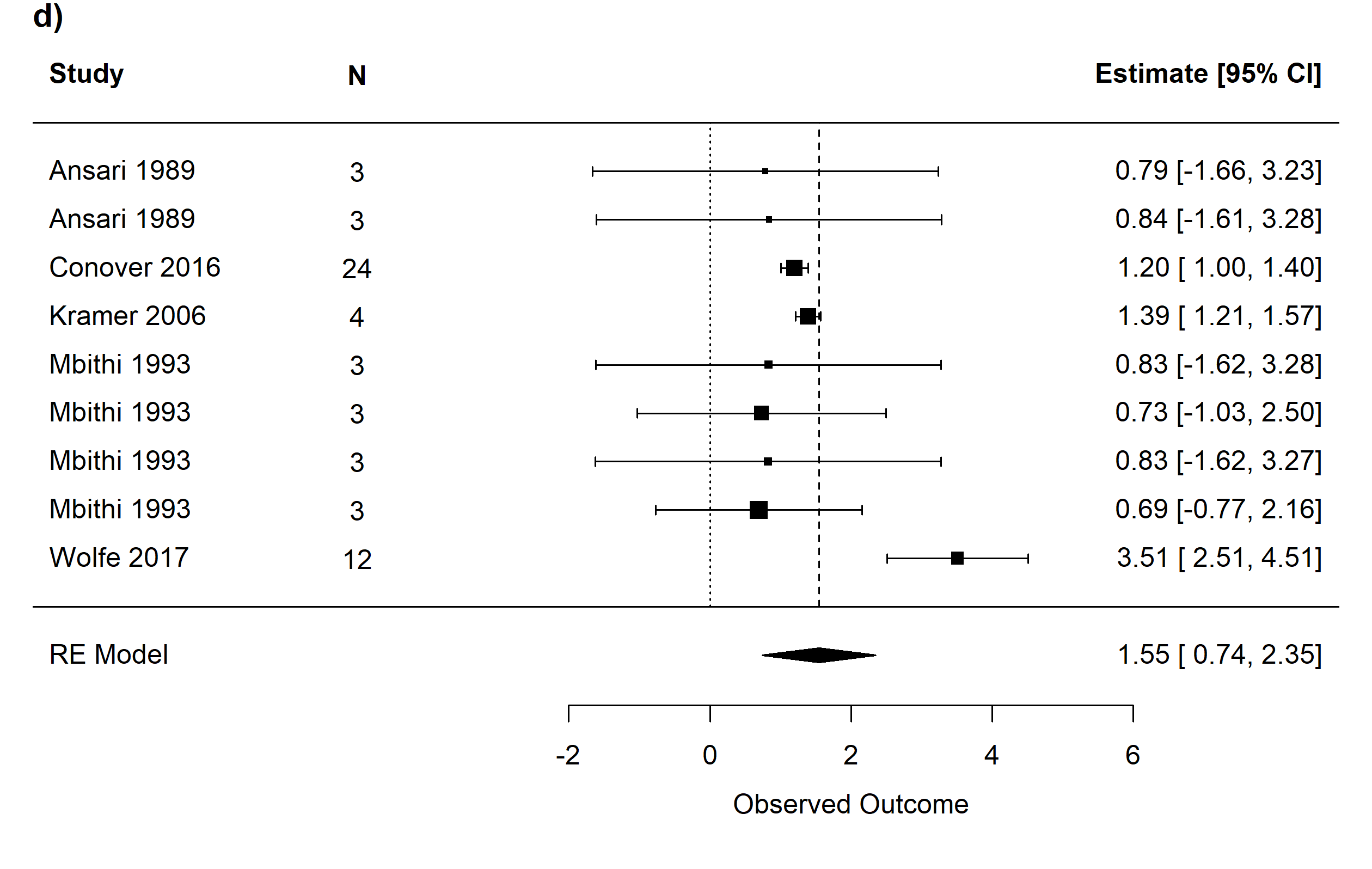


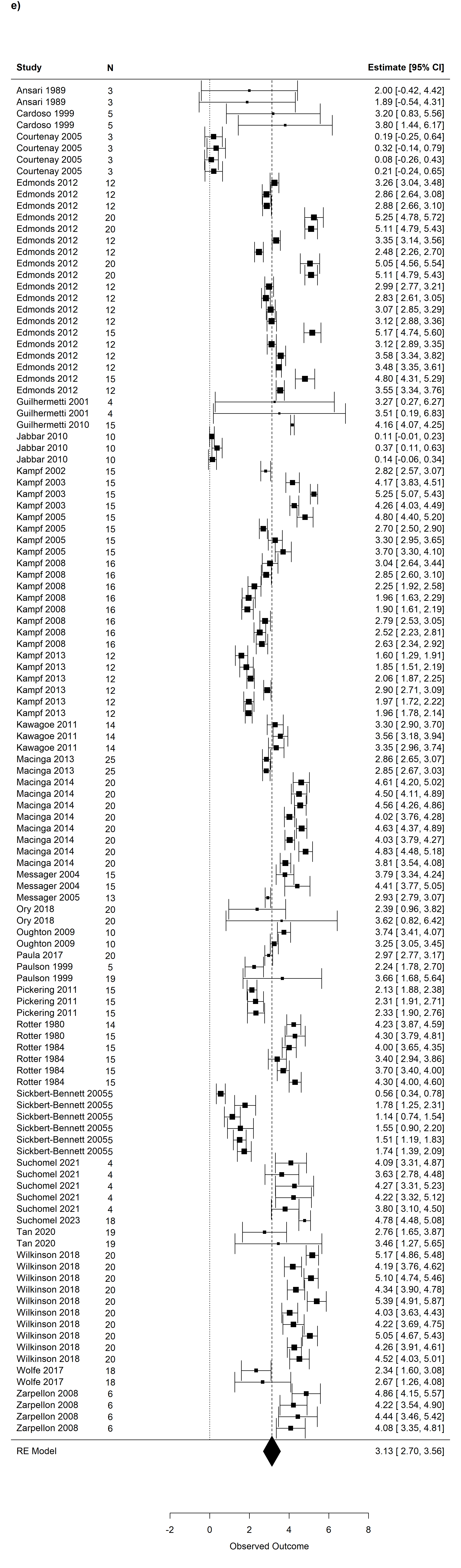


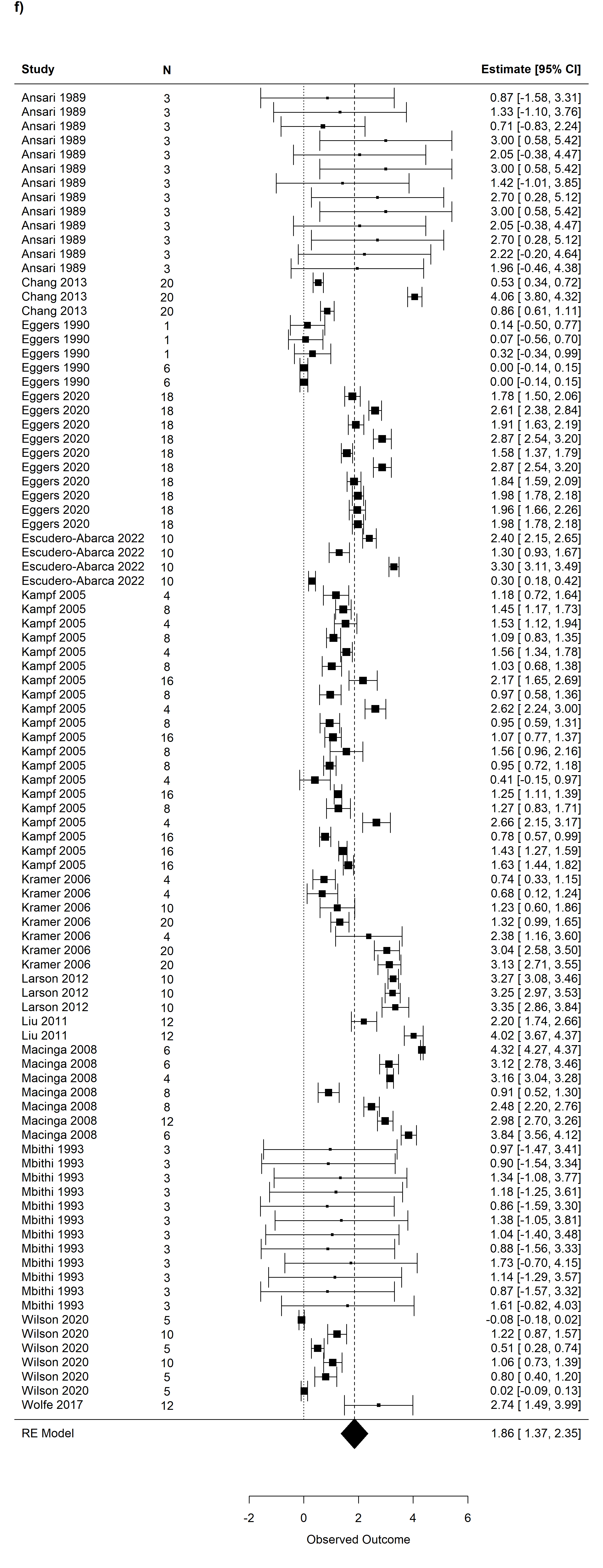


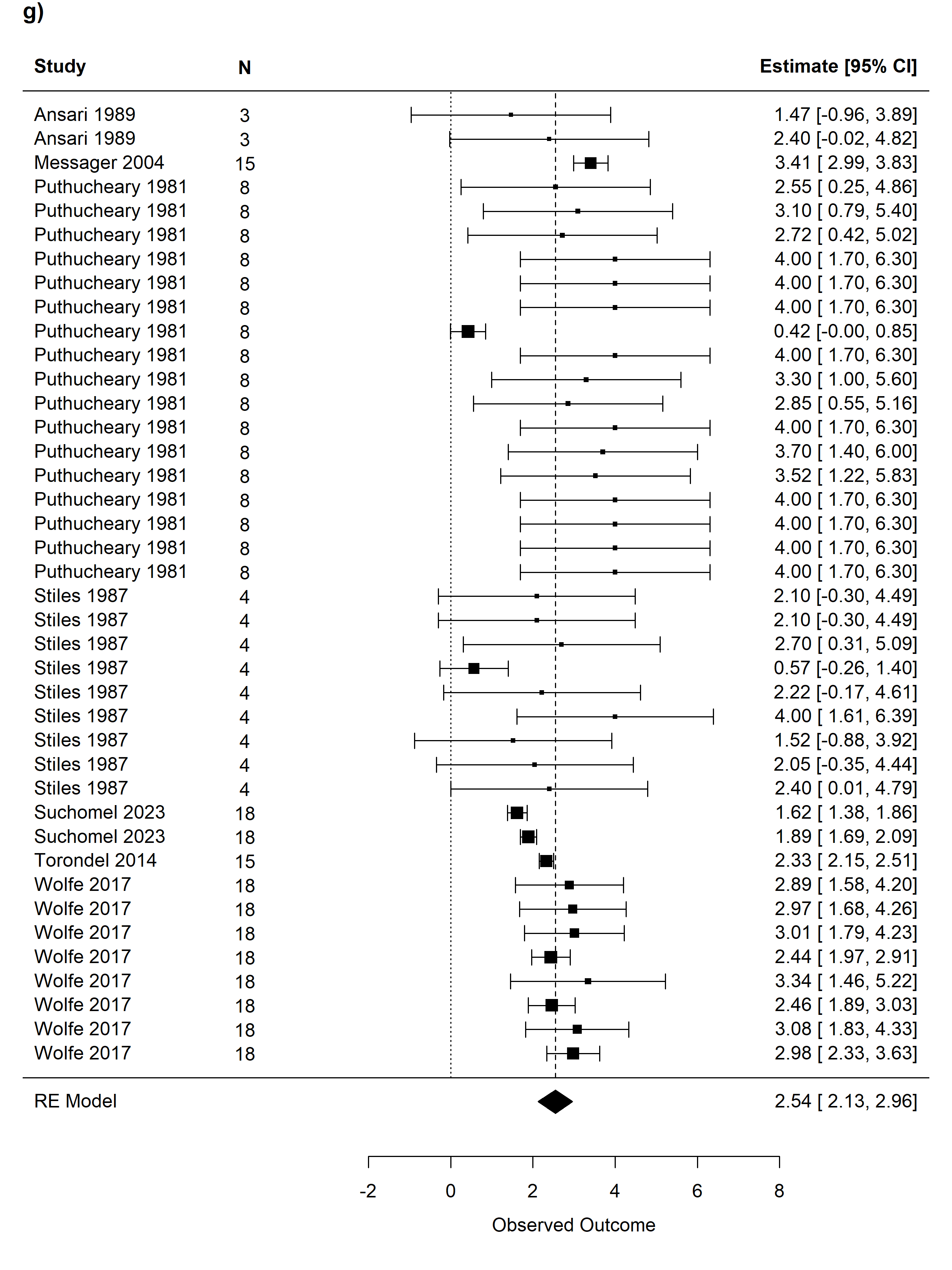


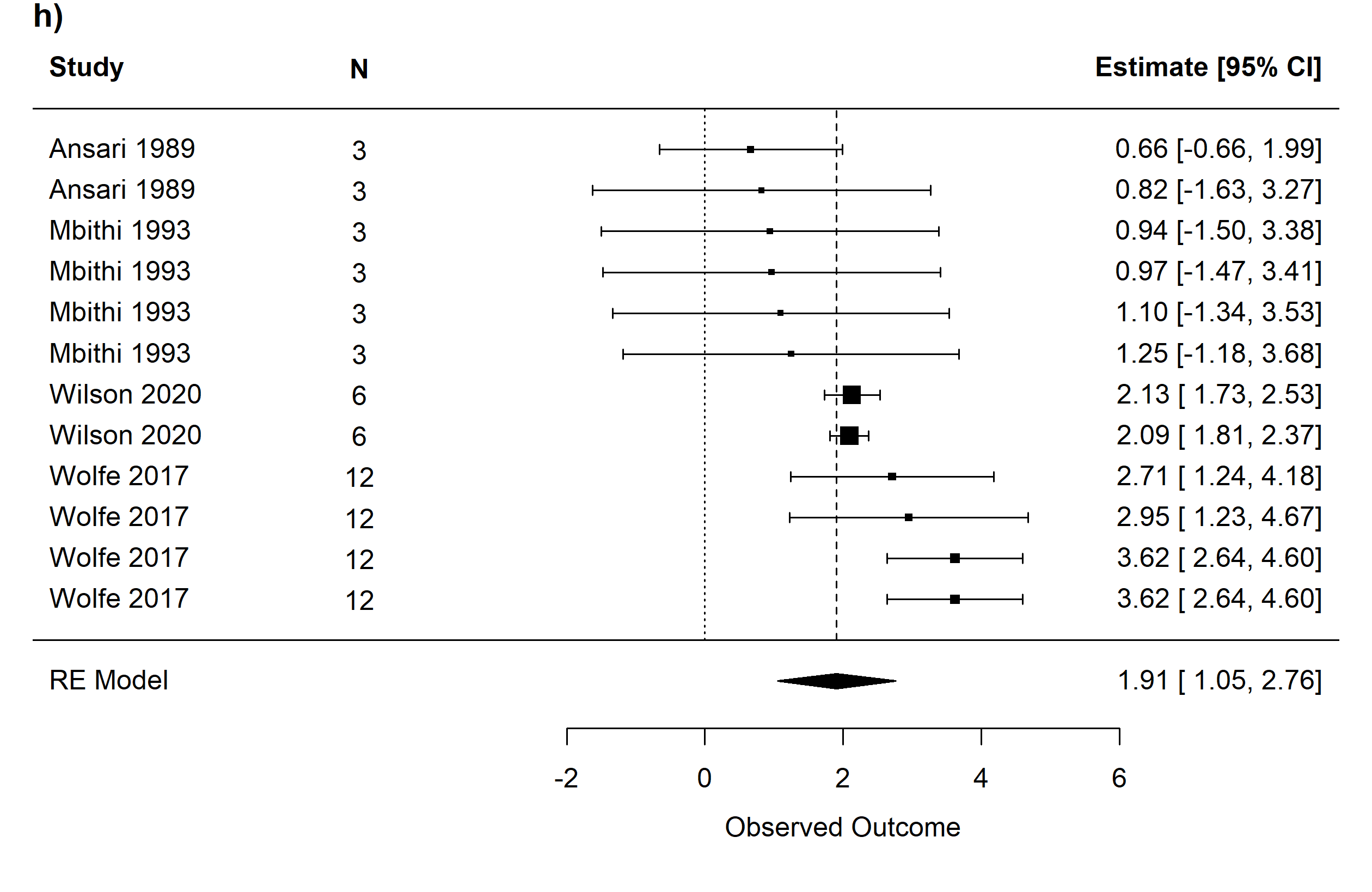


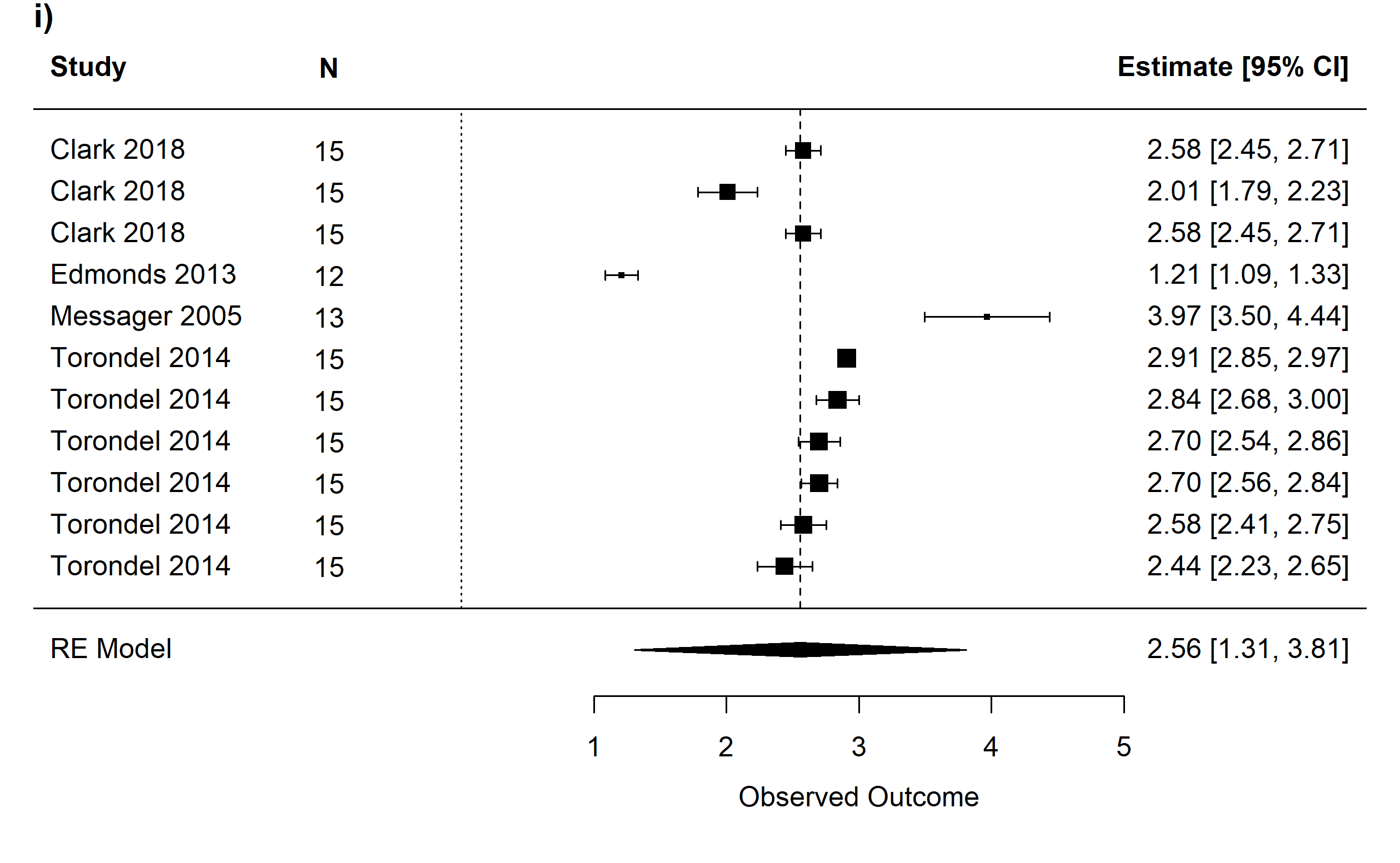


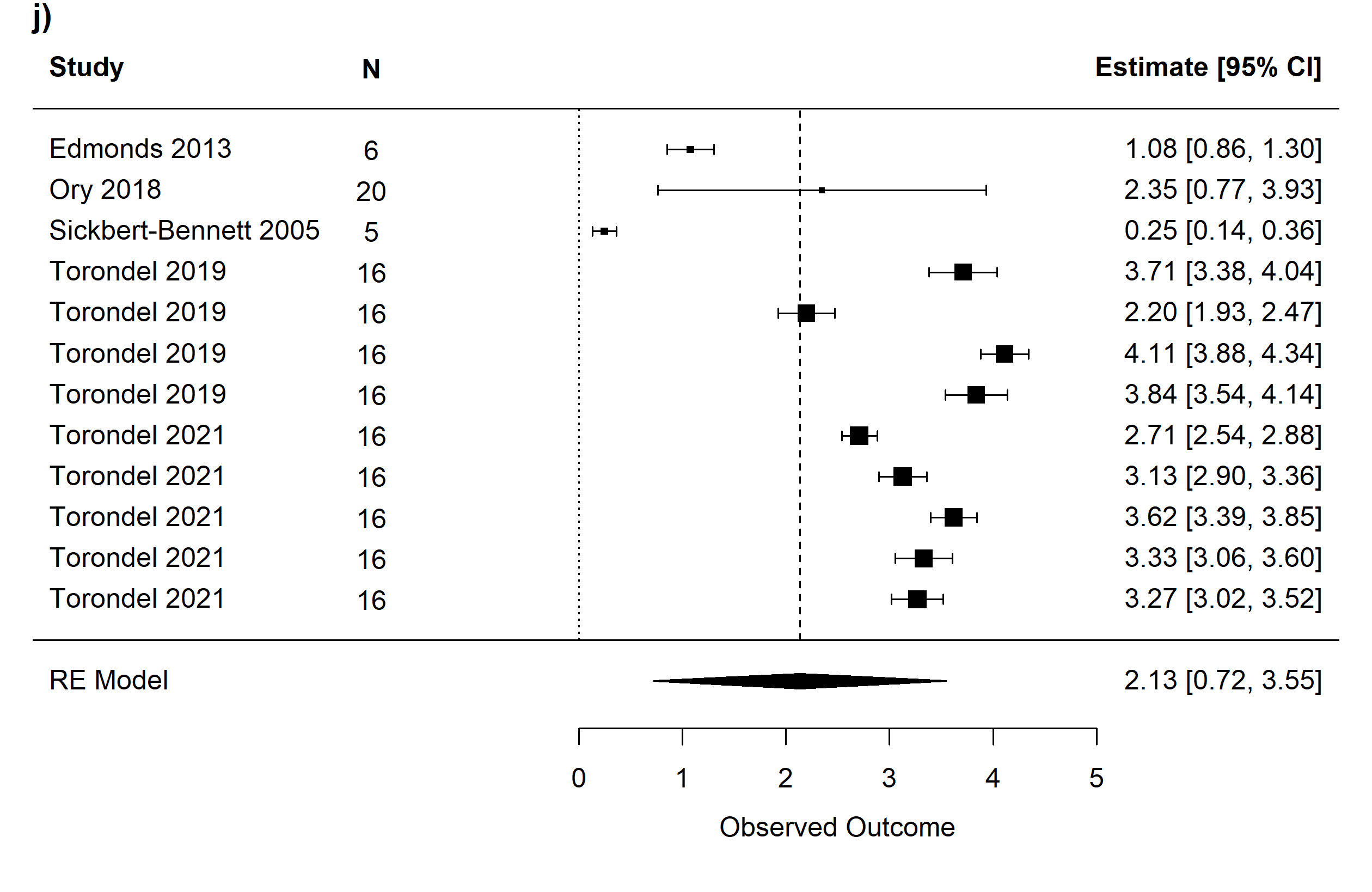

Supplement: online supplemental file 11 [file bmjgh-10-Suppl_7-s011.docx]
